# Association of mitochondrial DNA content, heteroplasmies and inter-generational transmission with autism

**DOI:** 10.1038/s41467-022-30805-7

**Published:** 2022-07-01

**Authors:** Yiqin Wang, Xiaoxian Guo, Xiumei Hong, Guoying Wang, Colleen Pearson, Barry Zuckerman, Andrew G. Clark, Kimberly O. O’Brien, Xiaobin Wang, Zhenglong Gu

**Affiliations:** 1 Division of Nutritional Sciences, Cornell University, Ithaca, NY USA; 2 Center on Early Life Origins of Disease, Department of Population, Family and Reproductive Health, Johns Hopkins University Bloomberg School of Public Health, Baltimore, MD USA; 3 Department of Pediatrics, Boston University School of Medicine and Boston Medical Center, Boston, MA USA; 4 Department of Molecular Biology and Genetics, Cornell University, Ithaca, NY USA; 5 Department of Computational Biology, Cornell University, Ithaca, NY USA; 6 Department of Pediatrics, Johns Hopkins University School of Medicine, Baltimore, MD USA; 7 Present Address: Center for Mitochondrial Genetics and Medicine, Greater Bay Area Institute of Precision Medicine (Guangzhou), Fudan University, Guangzhou, China

**Keywords:** DNA sequencing, Autism spectrum disorders, Mitochondrial genome

## Abstract

Mitochondria are essential for brain development. While previous studies linked dysfunctional mitochondria with autism spectrum disorder (ASD), the role of the mitochondrial genome (mtDNA) in ASD risk is largely unexplored. This study investigates the association of mtDNA heteroplasmies (co-existence of mutated and unmutated mtDNA) and content with ASD, as well as its inter-generational transmission and sex differences among two independent samples: a family-based study (*n* = 1,938 families with parents, probands and sibling controls) and a prospective birth cohort (*n* = 997 mother-child pairs). In both samples, predicted pathogenic (PP) heteroplasmies in children are associated with ASD risk (Meta-OR = 1.56, *P* = 0.00068). Inter-generational transmission of mtDNA reveals attenuated effects of purifying selection on maternal heteroplasmies in children with ASD relative to controls, particularly among males. Among children with ASD and PP heteroplasmies, increased mtDNA content shows benefits for cognition, communication, and behaviors (*P* ≤ 0.02). These results underscore the value of exploring maternal and newborn mtDNA in ASD.

## Abstract (web summary)

Most genetic studies of autism spectrum disorder (ASD) have focused on the nuclear genome. Here, the authors show that variations in mitochondrial DNA, detectable at birth, are also associated with risk of ASD.

## Introduction

Over the past eight years, most genetic studies of autism spectrum disorder (ASD) have focused on the nuclear genome, and have identified associations of common polymorphisms^1^, rare copy number variants, and damaging mutations^2–6^, with the risk of ASD. Collectively, these identified genomic variants may account for about 20% to 40% of ASD risk^1,6^, leaving a large fraction of ASD unexplained. More recently, there is growing evidence pointing to the potentially important role of mitochondria in the etiology of ASD. The 16.6-kb human mitochondrial genome (aka mitochondrial DNA; mtDNA) encodes 13 evolutionarily conserved proteins in four of the five oxidative phosphorylation (OXPHOS) protein complexes and 24 RNA genes essential for translation of mitochondrial proteins^7^. Recent studies indicate that purifying selection (the selective removal of deleterious alleles) act on germline mtDNA protecting against the irreversible accrual of damaging mutations in its inter-generational transmission^8–10^. Notably, mtDNA heteroplasmies (a state in which mutated mtDNA and unmutated mtDNA co-exist), either inherited from the mother or associated with de novo mutations in the maternal germline, were linked with mitochondrial disorders, mostly affecting tissues with heavy reliance on mitochondrial function^11^, such as the brain.

In animal studies, mice with heteroplasmic mtDNA were more susceptible to cognitive and behavioral deficits compared to their counterparts with homoplasmic mtDNA^12^; mitochondrial mutator mice born with inherited mtDNA mutations showed abnormal brain development^13^; transgenic mice carrying a nonsynonymous mtDNA mutation affecting OXPHOS complex I had impaired social interaction and repetitive behavioral defects resembling those of ASD^14^, indicating a potentially causal role of mtDNA variations in the pathogenesis of ASD, and perhaps, in other neurodevelopmental disabilities as well.

In humans, biochemical studies showed altered enzyme activities of varying OXPHOS complexes in brain tissues^15,16^ as well as peripheral tissues and cell lines^17–21^ of patients with ASD. Remarkably, mitochondrial dysfunction was suggested to affect about 30–50% of patients with ASD^18^. About 5% of patients with ASD meet the diagnostic criteria for classic mitochondrial disorders^18^. Previous studies using peripheral blood samples showed associations of a variety of sequence^22–24^ and content^24,25^ variations in mtDNA with ASD. From a clinical and public health perspective, it would not be feasible to obtain brain tissue for risk prediction or diagnostic purposes. Rather, it would be most useful and scalable to identify mtDNA biomarkers from peripheral blood samples.

To this end, in an earlier study, we examined mtDNA variations in relation to ASD by analyzing the whole-exome sequencing data from peripheral blood samples of 903 mother-proband-sibling trios in the Simons Simplex Collection (SSC)^22^. We revealed an elevation in pathogenicity of mtDNA heteroplasmies in OXPHOS genes among probands with ASD relative to their normally developing siblings^22^. However, the previous study was conducted using off-target reads from whole-exome sequencing and cross-sectional data from peripheral blood in only one cohort, raising questions regarding the replicability of the work and causal relationship between mtDNA heteroplasmy and autism onset.

**Fig. 1:**
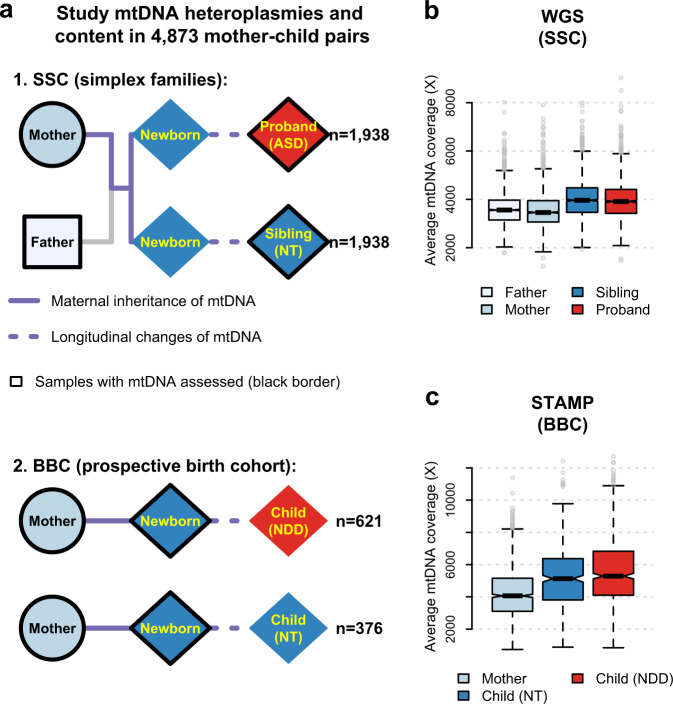
Study design and mtDNA evaluation in the SSC and BBC. **a** A schematic diagram of families (mother, father, sibling, and proband with ASD) in the SSC (*n* = 1938) and mother-child dyads in the BBC (*n* = 997). The square, circle, and diamond represent male, female, and both sexes, respectively. For both the SSC and BBC, the inter-generational transmission of mtDNA is represented by solid lines and the individual child longitudinal change in mtDNA is illustrated by dotted lines. **b**, **c** Box plots for the average mtDNA sequencing coverage among 1938 families in the SSC (as illustrated in **a**) using whole-genome sequencing (WGS) (**b**), and among 997 dyads of mothers and children in the BBC (as illustrated in **a**) using mtDNA-targeted sequencing (STAMP) (**c**). Box plots in **b** and **c** show the median as the center line, 95% confidence interval of the median as the notch, the first (Q1) and third (Q3) quartiles as the boundaries of the box, the values of the largest and smallest data points within the range between Q1–1.5× interquartile range (IQR) and Q3 + 1.5× IQR as the boundaries of the whiskers, and the outliers beyond this range as the gray points. ASD: autism spectrum disorder; NT: neurotypical; NDD: neurodevelopmental disorder.

This current study extends our past investigation in the following important ways. First, in terms of study design, we included two independent and complementary ethnically diverse cohorts (Fig. 1a): (1) SSC: we analyzed mtDNA variations derived from the whole-genome sequencing data of 1938 families consisting of both parents, probands, and sibling controls^26^; and (2) the Boston Birth Cohort (BBC): a prospective birth cohort study consisting of 997 pairs of mothers and children, a subset of the whole BBC, who were enrolled at birth with mother-newborn blood samples obtained at birth and followed prospectively from birth to age 21 years^27–29^. This longitudinal design allows us to evaluate the effect of a temporal relationship between maternal and newborn mtDNA variations at birth on a child’s future risk of developing ASD. Second, in terms of phenotypes, it is well-observed that there is considerable co-morbidity between ASD and other neurodevelopmental disorders (NDD)^28,29^, indicating the possibility of shared risk factors or molecular pathways. Thus, we further examined whether mtDNA variations in maternal and newborn pairs at birth may predispose a child to ASD in particular or NDD in general. Given the well-observed male dominance in ASD^30^ and other NDD^31,32^, we also explored sex differences. Third, methodologically, we performed ultradeep sequencing of mtDNA and systematically evaluated mtDNA variations, encompassing low-fraction mtDNA heteroplasmies, mtDNA content variations, and mtDNA inter-generational transmission and mutation patterns in both the SSC and BBC. The sequencing method (STAMP) that we developed for evaluating mtDNA heteroplasmies and content is reproducible and time- and cost-effective^33^, thus scalable in research, clinical, and public health settings.

Here, by analyzing ultradeep mtDNA sequencing data in two independent and complementary cohorts (SSC and BBC) in relation to ASD, we confirm that predicted pathogenic (PP) heteroplasmies increase the risk of ASD. Our results from the BBC further indicate that preferential transmission of mtDNA heteroplasmies with elevated pathogenicity, especially to male offspring, may be not only observed for ASD, but also for a broader range of NDD, including attention-deficit/hyperactivity disorder (ADHD) and other developmental delay (DD).

## Results

### Ultradeep mtDNA sequencing data

We obtained an average sequencing depth of 3778-fold(X) (interquartile range: 3249X–4225X, Fig. 1b) on mitochondrial genome (mtDNA) from the whole-genome sequencing (WGS) data of the SSC. We sequenced mtDNA in maternal and newborn samples from the BBC by using a targeted method called STAMP^33^ and achieved an average mtDNA sequencing depth of 4868X (interquartile range: 3482X–5879X, Fig. 1c). We identified 5628 mtDNA heteroplasmies in the SSC and 1574 mtDNA heteroplasmies in the BBC.

### Elevated pathogenicity of mtDNA heteroplasmies in ASD

**Fig. 2:**
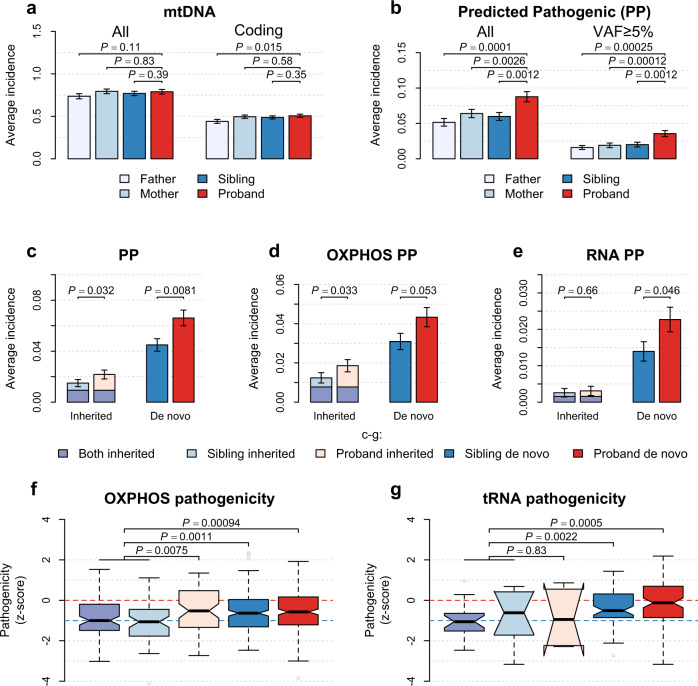
mtDNA heteroplasmies in the SSC. **a**, **b** Bar plots for the average number of heteroplasmies per participant **a** for all mtDNA heteroplasmies and heteroplasmies in the coding region, respectively; and **b** for predicted pathogenic (PP) heteroplasmies. (**c**) Bar plots for the average number of inherited and de novo PP heteroplasmies in probands and siblings. **d**, **e** Bar plots for the average number of PP heteroplasmies in oxidative phosphorylation (OXPHOS) genes (**d**) and in RNA genes (**e**) among probands and siblings. Data in bar plots (**a**–**e**) are mean ± SEM among 1938 families in the SSC as illustrated in Fig. 1a. Distributions of the number of mtDNA heteroplasmies per participant in **a** and **b** are shown in Supplementary Fig. 1. Two-sided *P* values from conditional logistic regression are shown. **f**, **g** Box plots for the distribution of pathogenicity z-scores among nonsynonymous heteroplasmies (from left to right, *n* = 89, 58, 65, 221, and 229) in OXPHOS genes (**f**) and among heteroplasmies (from left to right, *n* = 21, 10, 6, 67, and 102) in tRNA genes (**g**), to illustrate weakened effects of purifying selection on mtDNA heteroplasmies among children with ASD relative to their siblings. Box plots in **f** and **g** show the median as the center line, 95% confidence interval of the median as the notch, the first (Q1) and third (Q3) quartiles as the boundaries of the box, the values of the largest and smallest data points within the range between Q1–1.5× interquartile range (IQR) and Q3 + 1.5× IQR as the boundaries of the whiskers, and the outliers beyond this range as the gray points. *P* values (two-sided) in **f** and **g** are from the unpaired *t* test.

In line with our previous study^22^, we did not find a significant difference in the overall heteroplasmy number between probands and any other family members in the SSC (*P* ≥ 0.11, Fig. 2a and Supplementary Fig. 1); nor did we observe a significant increase in heteroplasmies in oxidative phosphorylation (OXPHOS) genes (*P* ≥ 0.057, Supplementary Table 1) among probands. However, medium-to-high-fraction (variant allele fraction [VAF] ≥ 5%) heteroplasmies in probands were more likely than those in siblings, to be detected at non-polymorphic mtDNA sites in the general population^34,35^ (Fisher’s exact test, odds ratio [OR] = 1.56, *P* = 0.011), which we previously found to be enriched for deleterious changes in OXPHOS genes^22^. In RNA genes, probands carried more heteroplasmies affecting tRNA genes relative to their siblings (*P* = 0.024) and parents (*P* ≤ 8.2 × 10^−6^, Supplementary Table 1). Since there are disproportionally more mtDNA mutations in tRNA genes known to cause mitochondrial disorders^36^ than those in rRNA genes (Fisher’s exact test, OR = 38.8, *P* < 2.2 × 10^−16^) or in OXPHOS genes (OR = 6.6, *P* < 2.2 × 10^−16^), we surmised that mtDNA heteroplasmies in probands might have elevated pathogenicity.

We confirmed that the number of predicted pathogenic (PP) heteroplasmies in mtDNA was increased in probands as compared to their siblings (OR = 1.51, *P* = 0.0012) and parents (OR ≥ 1.51, *P* ≤ 0.0026, Fig. 2b and Supplementary Fig. 1). The observed increase in PP heteroplasmies in probands persisted after we focused on heteroplasmies of medium-to-high fractions (versus siblings: OR = 2.15, *P* = 0.0012; versus parents: OR ≥ 2.20, *P* ≤ 0.00025; Fig. 2b). PP heteroplasmies affecting OXPHOS genes (OR = 1.50, *P* = 0.0083) and RNA genes (OR = 1.62, *P* = 0.041) were both significantly associated with ASD (Fig. 2d, e), extending our previous finding^22^ that not only changes in OXPHOS protein complexes but also defects in the translational machinery within mitochondria may increase ASD risk.

Moreover, PP heteroplasmies in mtDNA may not be modulated by nuclear risk factors for ASD, since neither polygenic risk scores (PRS) of ASD-associated common variants^1^ nor burden of damaging de novo variants in the nuclear genome^4–6^ previously reported in the SSC correlated with PP heteroplasmies among probands (*P* ≥ 0.1, Supplementary Table 2). Remarkably, the effect of PP heteroplasmies on ASD was diminished (OR = 1.13, *P* = 0.62) among families with the proband in the 95th percentile for PRS of ASD^1^ or carrying de novo variants, including likely gene-disrupting mutations^4^, structural coding variants^5^, and excessive damaging noncoding mutations^6^ near ASD risk genes^3^, in the nuclear genome (Supplementary Table 3). The association between PP heteroplasmies and ASD was further strengthened after adjustment for ASD-associated nuclear variants in the SSC (OR = 1.59; *P* = 0.00052, Supplementary Table 3), indicating that PP heteroplasmies may be an independent genetic risk factor for ASD.

### Biased maternal transmission of mtDNA heteroplasmies in ASD

We found that the proportion of maternally inherited heteroplasmies did not differ between probands and siblings in the SSC (chi-squared test, *P* = 0.46). Of PP heteroplasmies, about 25% in probands were inherited from the mother (versus siblings: VAF-adjusted *P* = 0.85). Both inherited and de novo PP heteroplasmies were significantly increased in probands relative to their siblings (OR ≥ 1.43, *P* ≤ 0.032, Fig. 2c).

Overall, nonsynonymous (NS) heteroplasmies in OXPHOS genes and heteroplasmies in tRNA genes transmitted from the mother to the sibling showed average pathogenicity below zero (*P* for selection <10^−5^, Supplementary Table 4), suggesting strong selection against pre-existing deleterious mtDNA heteroplasmies in the maternal lineage. Of note, compared to inherited NS heteroplasmies in siblings, those transmitted solely to probands increased in pathogenicity (*P* = 0.0075, Fig. 2f; VAF-adjusted *P* = 0.007), suggesting biased transmission of NS heteroplasmies with elevated pathogenicity to children with ASD. We also found evidence of the effects of purifying selection on de novo NS heteroplasmies in both siblings and probands (*P* for selection <10^−5^, Supplementary Table 4**)**, indicating that purifying selection acts on new mutations in mtDNA.

In RNA genes, over 86% of PP heteroplasmies were de novo mutations, which differed significantly between probands and siblings (*P* = 0.046, Fig. 2e). Among siblings, de novo tRNA heteroplasmies were subject to purifying selection (one-sample *t* test, *P* = 0.012, Supplementary Table 4), despite an increase in their pathogenicity relative to inherited ones (*P* = 0.0022, Fig. 2g; VAF-adjusted *P* = 0.015). In contrast, average pathogenicity of de novo tRNA heteroplasmies among probands did not significantly deviate from that of random variants in tRNA genes (*P* for selection ≥0.076, Supplementary Table 4), suggesting attenuated effects of purifying selection on mitochondrial tRNA mutations in ASD.

### Maternal age at childbirth affects mtDNA heteroplasmies

**Fig. 3:**
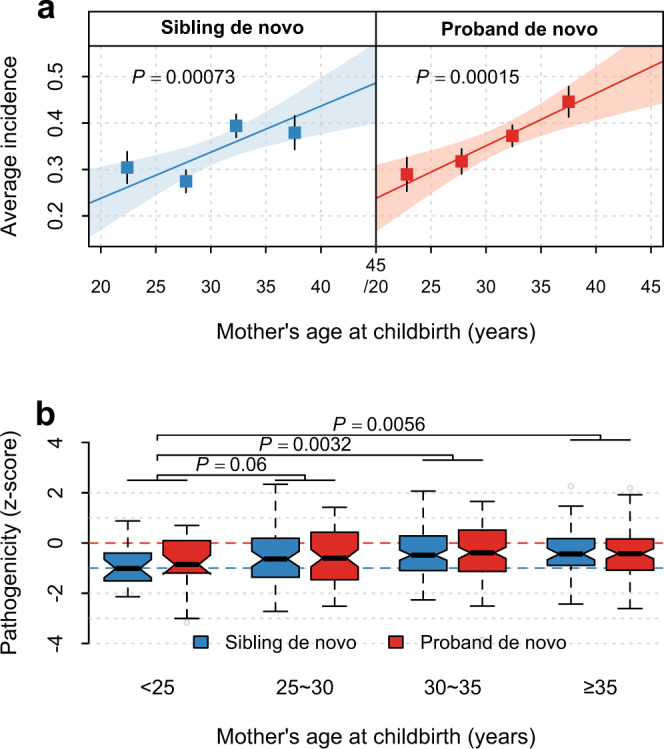
Influence of maternal age at childbirth on de novo heteroplasmies in the SSC. **a** Maternal age-dependent increase in the number of de novo heteroplasmies in siblings and probands. Points and error bars in each subpanel represent mean ± SEM among the four maternal-age groups (from left to right, *n* = 217, 514, 665, and 327 siblings, and n = 197, 542, 725, and 471 probands). 215 siblings and 3 probands with missing maternal age information were excluded from the analyses in (**a**) and (**b**). The *P* value (two-sided) and the shaded area indicate the significance of the slope and the 95% confidence interval of the linear regression line (the solid line), respectively. No significant difference in slope was found between cases and controls, as shown in Supplementary Table 12. **b** Box plots for the distribution of pathogenicity z-scores of de novo heteroplasmies at nonsynonymous sites in oxidative phosphorylation genes or at all sites in tRNA genes stratified by maternal age groups at birth (from left to right, *n* = 23, 63, 113, and 53 in siblings, and *n* = 29, 73, 133, and 93 in probands). Two-sided *P* values are from linear regression. Box plots show the median as the center line, 95% confidence interval of the median as the notch, the first (Q1) and third (Q3) quartiles as the boundaries of the box, the values of the largest and smallest data points within the range between Q1–1.5× interquartile range (IQR) and Q3 + 1.5× IQR as the boundaries of the whiskers, and the outliers beyond this range as the gray points.

We found that mother’s age at childbirth correlated positively with the number of de novo heteroplasmies (*P* ≤ 0.00073, Fig. 3a) in both probands and siblings in the SSC but did not correspond to the number of inherited ones (*P* ≥ 0.12, Supplementary Table 5). The observed correlation was also tested to be significant at NS sites in OXPHOS genes and in tRNA genes (*P* = 0.00044). Further control for paternal age in the analyses did not qualitatively alter the observed effects of maternal age (*P* for maternal age ≤0.019, Supplementary Table 5*; P* for paternal age ≥0.32).

Moreover, children born to mothers aged under 25 years showed significantly decreased pathogenicity among de novo NS and tRNA heteroplasmies as compared to those born to older mothers (*P* ≤ 0.0056, versus maternal-age groups 30–35 years and ≥35 years, Fig. 3b; *P* for trend=0.0069 and =0.023 after control for paternal age at childbirth, Supplementary Table 6*)*. These results indicate that advanced maternal age at childbirth may contribute to an accumulation of mutations and attenuated effects of purifying selection on heteroplasmies in mtDNA.

### mtDNA content decreases in ASD and modifies the impact of mtDNA heteroplasmies

**Fig. 4:**
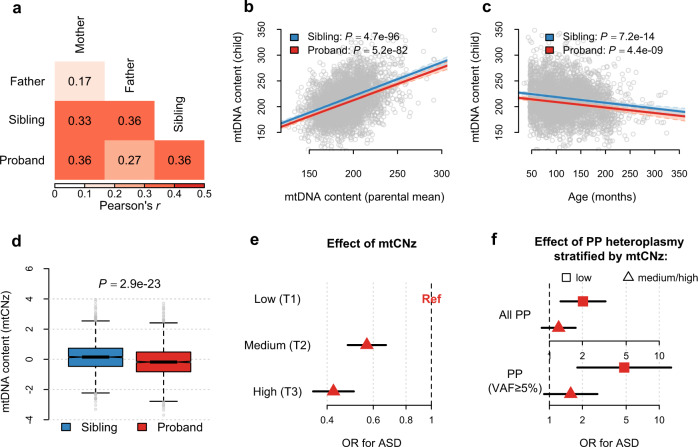
mtDNA content in the SSC. All analyses were performed in 1938 families of the SSC as illustrated in Fig. 1a. **a** Pearson’s correlation coefficients of mtDNA content between the father, mother, sibling and proband. **b** Scatter plots for mtDNA content in siblings and probands in relation to the mean values of mtDNA content in their parents. **c** Scatter plots for mtDNA content in siblings and probands in relation to their age at the time of sample collection. 215 siblings with missing age information were excluded. In **b** and **c**, the solid lines represent the linear regression lines estimated among siblings and probands, respectively, and the *P* value (two-sided) indicates the significance of the slope. **d** Box plots for the distribution of mtDNA content z-scores (mtCNz) in siblings and probands. All box-plot elements are defined in the same way as in Fig. 1. The *P* value (two-sided) was obtained from conditional logistic regression, where ASD is the outcome and mtCNz is the predictor. **e** Forest plots for the effects of mtCNz on ASD risk. The low-tertile group (T1) of mtCNz served as the reference group. **f** Forest plots for the effects of predicted pathogenic (PP) heteroplasmies on ASD risk among children with low mtCNz (the low-tertile group; T1) vs. medium/high mtCNz (the middle- or high-tertile group; T2/T3). The upper subpanel and lower subpanel show the results for all PP heteroplasmies and PP heteroplasmies with variant allele fraction (VAF) ≥ 5%, respectively. Horizontal black lines represent the 95% confidence interval (CI) of the estimated odds ratio (OR). Values on the x axis are shown on a logarithmic scale.

We found moderate correlations of mtDNA content at *r* = 0.27–0.36 between parents and children (*P* ≤ 2.2 × 10^−34^, Fig. 4a) in the SSC. The narrow-sense heritability of mtDNA content in blood was 0.65 (95% confidence interval [CI]: 0.59–0.71) for siblings and was 0.63 (95% CI: 0.57–0.69) for probands (Fig. 4b), close to the heritability identified in a twin study of mtDNA content^37^. Moreover, significant negative correlations were detected between mtDNA content and age of both probands and siblings (*P* ≤ 4.4 × 10^−9^, Fig. 4c). We then computed a z-score (mtCNz) of mtDNA content among children by taking the standardized residuals from regression of the offspring mtDNA content on age and the parental mean of mtDNA content.

We found a mild but statistically significant decrease in mtCNz in probands relative to their siblings (*P* = 2.9 × 10^−23^, Fig. 4d). Decline in mtCNz remained significant among probands after we stratified sibling-proband pairs according to their sex or reported parental races (*P* ≤ 9.6 × 10^−5^, Supplementary Table 7). As compared to children in the low-tertile (T1) group of mtCNz, children in the middle-tertile group (T2) and the high-tertile group (T3) of mtCNz showed a decreased risk of ASD at OR = 0.57 (95% CI: 0.48–0.67) and OR = 0.42 (95% CI: 0.35–0.51), respectively (Fig. 4e).

After incorporating PP heteroplasmies and parental ages at childbirth into the analysis, we found that both PP heteroplasmies and low mtCNz (T1 of mtCNz) were associated with ASD risk (OR ≥ 1.50, *P* ≤ 0.0024). Of note, an interaction effect between PP heteroplasmies and low mtCNz on ASD risk was suggested (*P* for interaction≤0.083, Supplementary Table 8). Among children with low mtCNz (in T1 of mtCNz), carrying PP heteroplasmies led to an increased risk of ASD at OR = 2.02 (*P* = 0.0033), and at OR = 4.80 (*P* = 0.0018) if the heteroplasmy was of medium-to-high fractions (VAF ≥ 5%; Fig. 4f). In contrast, among children with normal mtCNz (in T2/T3 of mtCNz), the ORs of carrying PP heteroplasmies for ASD became smaller and nonsignificant (OR = 1.22, *P* = 0.28; OR = 1.56, *P* = 0.12 for those of VAF ≥ 5%; Fig. 4f), suggesting that increased mtDNA content may partially mitigate the deleterious effects of PP heteroplasmies in ASD.

### mtDNA content and heteroplasmies associated with ASD-related neurological traits

**Fig. 5:**
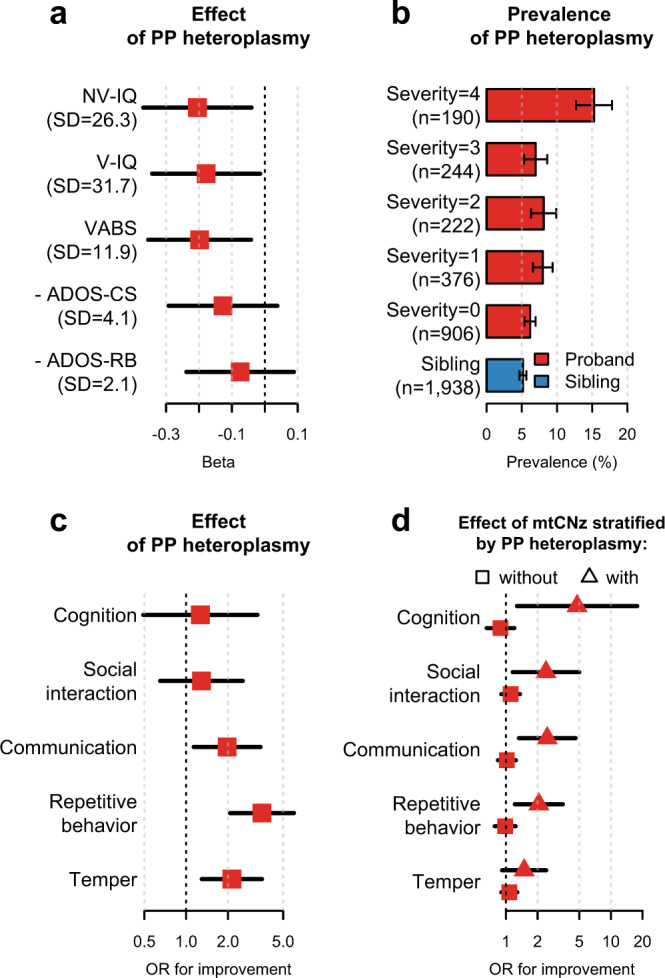
Association of mtDNA heteroplasmies and mtDNA content with neurodevelopmental traits among probands in the SSC. **a** Forest plots for the effects of carrying predicted pathogenic (PP) heteroplasmies on ASD-related neurodevelopmental traits among probands (*n* = 1938 for all traits). Beta refers to the standardized coefficient, and negative values indicate worse functions. The standard deviation (SD) of each trait is indicated in parentheses. **b** Bar plots for the family-adjusted prevalence of PP heteroplasmies among probands and siblings. Probands were grouped according to the severity level computed using the equation: (NV-IQ < 70) + (V-IQ < 70) + (VABS in the bottom 25%) + (- ADOS-CS in the bottom 25%). **c** Forest plots for the odds ratios (ORs) of carrying PP heteroplasmies for improvements in ASD-related symptoms among probands (from top to bottom, *n* = 1502, 1512, 1510, 1512, and 1516). **d** Forest plots for the favorable effects of per standard deviation increase in the mtDNA content z-score (mtCNz) among probands without PP heteroplasmies (from top to bottom, *n* = 1383, 1391, 1389, 1390, and 1395) and among probands with PP heteroplasmies (from top to bottom, *n* = 119, 121, 121, 122, and 121). Horizontal black lines represent the 95% confidence interval of the effect sizes. Error bars in **b** represent bootstrapped SEM. Values on the x axes in **c** and **d** are shown on a logarithmic scale. NV-IQ: non-verbal IQ; V-IQ: verbal IQ; VABS: the individual’s Adaptive Behavior Composite from Vineland Adaptive Behavior Scales (2nd edition); ADOS-CS: the communication and social total from the Autism Diagnostic Observation Schedule (ADOS); ADOS-RB: the restricted and repetitive behavior total (module 1–3) or the stereotyped behaviors and restricted interests total (module 4) from the ADOS. Because the ADOS records scores of abnormalities, the additive inverse of the original value was used.

Among probands with ASD in the SSC, carrying PP heteroplasmies was associated with decreased non-verbal IQ (*P* = 0.015) and verbal IQ (*P* = 0.034, Fig. 5a), and increased risks of intellectual disability (non-verbal IQ < 70: OR = 1.70, *P* = 0.0036; verbal IQ < 70: OR = 1.41, *P* = 0.05; Supplementary Table 9), supporting a previous report that children with ASD and co-occurring intellectual disability had characteristic OXPHOS deficiencies suggesting mitochondrial disorders^19^.

Moreover, probands with PP heteroplasmies were more likely to score in the lower quartile in the assessment of neurodevelopmental functions as measured by the Vineland Adaptive Behavior Scales (VABS, 2nd edition; OR = 1.86, *P* = 0.00079; Supplementary Table 9), as well as communication and social skills as measured by the total score for the communication and social components in the Autism Diagnostic Observation Schedule (ADOS; [-] ADOS-CS: OR = 1.66, *P* = 0.0039; Supplementary Table 9). We then computed a severity score based on non-verbal and verbal IQ (IQ < 70) as well as the VABS and ADOS-CS (in the lower quartile of the respective assessment). Strikingly, the family-adjusted prevalence of PP heteroplasmies exceeded 15% among probands at the highest severity level (Fig. 5b).

However, probands carrying PP heteroplasmies did not show an increase in restricted and repetitive behaviors as measured by the ADOS ([-] ADOS-RB: *P* ≥ 0.31; Fig. 5a and Supplementary Table 9). Instead, they were more likely to report improvements in autism symptoms related to repetitive behaviors (OR = 3.52, *P* = 3.3 × 10^−6^), temper (OR = 2.13, *P* = 0.0029), and communication (OR = 1.97, *P* = 0.016) relative to probands without PP heteroplasmies (Fig. 5c). These results are in line with a recent study showing that mitochondrial OXPHOS only indirectly affects behavioral symptoms of autism^38^. Moreover, none of the observed associations were affected by further adjustment for ASD-related therapies (Supplementary Table 9).

Interestingly, we found that increased mtDNA content conferred protection against ASD-related neurological defects only among probands carrying PP heteroplasmies (Fig. 5d): a one standard deviation increase in mtCNz was associated with about two to five times the odds of reporting an improvement in cognition, social interaction, communication, and repetitive behaviors (*P* ≤ 0.02, Fig. 5d). In contrast, nonsignificant effects of mtCNz on the associated neurological improvements were identified among probands without PP heteroplasmies (OR ≤ 1.11, *P* ≥ 0.33, Fig. 5d; *P* for heterogeneity ≤0.045).

### mtDNA content and heteroplasmies in cord blood of children diagnosed with NDD

As the study in the SSC was designed for cross-sectional research^26^, to establish the temporal relationship between mtDNA variations and autism, we sequenced mtDNA in a total of 1067 pairs of umbilical cord blood and maternal blood samples collected at childbirth in the BBC^27–29^. After quality filtering, we obtained samples from 621 children with at least one diagnosis of NDD at postnatal visits: 82 with ASD; 221 with attention-deficit/hyperactivity disorder (ADHD) without co-occurring ASD; and 318 with developmental delay (DD) or other NDD without co-occurring ASD and ADHD (Supplementary Fig. 2). We relied on a common, neurotypical (NT) group comprising 376 children without any NDD diagnosis as controls in the BBC.

We observed a moderate correlation of mtDNA content at *r* = 0.26 between newborns and their mothers (*P* = 4.9 × 10^−17^; *r* ≥ 0.24 among NT children and children with NDD, separately; Supplementary Fig. 2). mtDNA content in cord blood also showed negative correlations with gestational age in both NT children and children with NDD (*P* ≤ 0.017, Supplementary Fig. 2). We then computed a z-score (mtCNz) of mtDNA content in cord blood with adjustment for mother’s mtDNA content and gestational age.

We found that mtCNz was decreased among children with NDD relative to NT children (*P* = 0.096) and was negatively correlated with the number of NDD conditions (*P* = 0.045, Supplementary Fig. 2). Notably, the decline in mtCNz was significant and more pronounced among children with NDD born preterm (*P* ≤ 0.036), suggesting an interaction effect between low mtDNA content and prematurity on NDD (*P* for interaction ≤ 0.048; likelihood-ratio test for mtCNz and the interaction term, *P* ≤ 0.025). Comparable interaction effects were also observed for each of the three NDD groups in the BBC: ASD, ADHD not ASD, and DD/other only (*P* for interaction ≤ 0.089, Supplementary Table 10). These results imply that the influence of deficient mitochondrial function on neurodevelopment may be more profound if the child is born prematurely^39^.

**Fig. 6:**
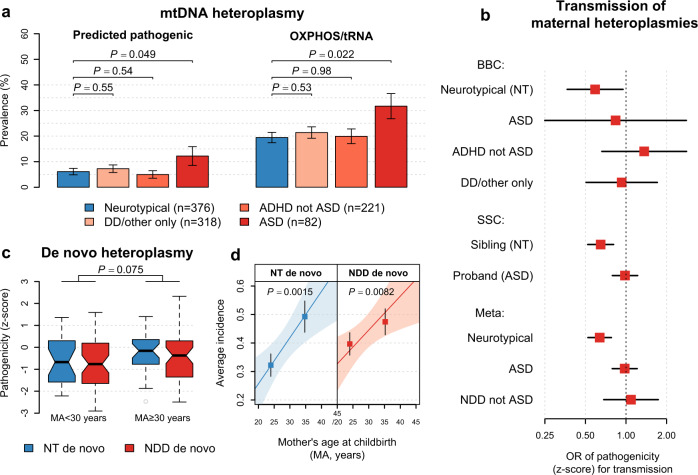
Replication of findings on mtDNA heteroplasmy prevalence and transmission as well as maternal age effect in the BBC. **a** Bar plots for the family-adjusted prevalence of predicted pathogenic (PP) heteroplasmies and heteroplasmies at nonsynonymous sites in oxidative phosphorylation genes or at all sites in tRNA genes (OXPHOS/tRNA), respectively. Error bars represent bootstrapped SEM. Two-sided *P* values are from logistic regression further adjusted for mtDNA content z-score. **b** Forest plots for the effects of per standard deviation increase in pathogenicity z-score on transmission of maternal mtDNA heteroplasmies to the child. Horizontal black lines represent the 95% confidence interval of the odds ratio (OR) among children indicated by the label on the y axis (from top to bottom, BBC: *n* = 376, 82, 221, and 318 children; SSC: *n* = 1938 siblings and 1938 probands; Meta: *n* = 2314, 2020, and 539 children). Combined effects were estimated based on a fixed-effect meta-analysis (Meta) of the results from the SSC and BBC. Values on the x axis are shown on a logarithmic scale. **c** Box plots for the distribution of pathogenicity z-scores among de novo heteroplasmies (from left to right, NT: *n* = 24, 30; NDD: *n* = 52, 50) in children born to mothers aged below and above 30 years. Two-sided *P* values are from linear regression. All box-plot elements are defined in the same way as in Fig. 1. **d** Maternal age-dependent increase in the number of de novo heteroplasmies. Points and error bars represent mean ± SEM among children (from left to right, NT: *n* = 242, 134; NDD: *n* = 368, 253) in the two maternal-age groups in (**c**). The *P* value (two-sided) and the shaded area indicate the significance of the slope and the 95% confidence interval of the linear regression line (the solid line), respectively. ASD: autism spectrum disorder; ADHD: attention-deficit/hyperactivity disorder; DD: developmental delay; NDD: neurodevelopmental disorder.

Supporting our finding in the SSC, PP heteroplasmies showed higher family-adjusted prevalence among children with ASD as compared to NT children (OR = 2.32, *P* = 0.049; Fig. 6a). After performing a fixed-effect meta-analysis to combine results from the SSC and BBC, we estimated an OR of carrying PP heteroplasmies for ASD to be 1.56 (*P* = 0.00068), and 2.28 (*P* = 0.00016) if the heteroplasmy was of medium-to-high fractions. Neither was significantly altered after control for mtDNA content and maternal age at childbirth (*P* ≤ 0.00098; model 2 in Table 1). The population-attributable risk proportion (PAR) of carrying PP heteroplasmies for ASD was estimated to be 2.9% (95% CI: 1.2–4.7%), of which about one third (PAR = 1.0%; 95% CI: 0.35–2.0%) resulted from inherited heteroplasmies and two thirds (PAR = 1.9%; 95% CI: 0.41–3.5%) was due to de novo mtDNA mutations.

**Table 1:** Association of predicted pathogenic heteroplasmies with ASD risk in the SSC and BBC, respectively, and in combined samples of the SSC and BBC.

PP heteroplasmy	Model 1				Model 2			
	SSC	BBC	Meta		SSC	BBC	Meta	
	OR	OR	OR^a^	*P* (het)^b^	OR	OR	OR^a^	*P* (het)^b^
	[95% CI]	[95% CI]	[95% CI]		[95% CI]	[95% CI]	[95% CI]	
All	1.50	2.32	1.56	0.33	1.50	2.20	1.55	0.40
	[1.14–1.96]	[1.00–5.38]	[1.21–2.02]		[1.14–1.97]	[0.94–5.16]	[1.19–2.01]	
VAF ≥ 5%	2.17	4.04	2.28	0.44	2.16	3.96	2.27	0.45
	[1.39–3.38]	[0.89–18.35]	[1.49–3.49]		[1.38–3.39]	[0.87–17.90]	[1.48–3.50]	
Inherited	2.13	2.17	2.13	0.99	2.21	2.57	2.24	0.89
	[1.15–3.92]	[0.32–14.81]	[1.19–3.82]		[1.19–4.10]	[0.36–18.44]	[1.24–4.04]	
De novo	1.40	1.88	1.44	0.54	1.38	1.67	1.41	0.70
	[1.04–1.88]	[0.75–4.68]	[1.08–1.91]		[1.02–1.87]	[0.66–4.25]	[1.05–1.87]	

Model 1: SSC: ~ carrying PP heteroplasmies + mtCNz + strata(family id); BBC: ~ carrying PP heteroplasmies + mtCNz + sex + mtDNA-inferred ancestry; Model 2: SSC: model 1 + maternal age at childbirth + paternal age at childbirth + child age at the time of sample collection; BBC: model 1 + maternal age at childbirth.

^a^The OR (odds ratio) of carrying predicted pathogenic (PP) heteroplasmies for ASD were estimated based on fixed-effect meta-analysis (Meta) of the results from the SSC and BBC; the ORs of carrying inherited PP heteroplasmies and de novo PP heteroplasmies were assessed jointly as: ~ de novo heteroplasmy + inherited heteroplasmy + covariates.

^b^Q statistic *P* values for heterogeneity in the ORs estimated between the SSC and BBC. The null distribution of Q statistic follows a chi-square distribution with 1 degree of freedom.

However, the associations between mtDNA heteroplasmies and the risks of other NDD were not revealed until we focused on heteroplasmies with high fractions in cord blood. For example, the associations of heteroplasmies at NS sites in OXPHOS genes and in tRNA genes with NDD other than ASD grew to be significant after we focused on heteroplasmies of VAF ≥ 20% (OR = 2.39, *P* = 0.035; OR for ADHD = 2.75, *P* = 0.038; Supplementary Table 11).

### Germline selection and maternal age affect mtDNA heteroplasmies in cord blood

Of heteroplasmies detected in cord blood of children, 47% were shared with their mothers, and 23% showed an increase in VAF relative to their mothers’ heteroplasmies (children with NDD versus NT children: chi-squared test, *P* ≥ 0.33). Among NT children, transmission of a heteroplasmy from the mother was negatively associated with its pathogenicity z-score (VAF-adjusted *P* = 0.030). Both inherited and de novo heteroplasmies in cord blood of NT children showed evidence of purifying selection (*P* for selection ≤0.012, Supplementary Table 4), supporting that selective removal of deleterious mtDNA heteroplasmies occurs in the germline or the embryo.

Of note, the effect of purifying selection on mothers’ heteroplasmies was weakened among children with NDD, with pathogenicity of transmitted heteroplasmies indistinguishable from that of untransmitted ones (*P* ≥ 0.40 among children with ASD, ADHD, and DD/other, separately, Fig. 6b). Based on related results from the SSC and BBC (Fig. 6b), we estimated that a one standard deviation increase in pathogenicity z-scores of maternal heteroplasmies was associated with reduced odds of transmission to NT children (Meta-OR = 0.64, *P* = 1.1 × 10^−5^) but did not affect heteroplasmy transmission to children with ASD (Meta-OR = 0.98, *P* = 0.84; versus NT children: *P* for heterogeneity = 0.0041, I^2^ = 88%) and to children with other NDD (Meta-OR = 1.09, *P* = 0.72; versus NT children: *P* for heterogeneity = 0.038, *I*^2^ = 77%).

Moreover, the number of de novo heteroplasmies in cord blood increased with maternal age at childbirth (*P* ≤ 0.0082) at similar rates in NT children and children with NDD (Fig. 6d; *P* for heterogeneity = 0.52). Both rates were comparable to the rates identified in peripheral blood of children in the SSC (*P* for heterogeneity ≥ 0.28, Supplementary Table 12). Accordingly, we estimated mtDNA mutation rate to be 2.4 × 10^−7^ per site per generation (Supplementary Table 13), close to the findings from previous studies (2.7–4.7 × 10^−7^ per site per generation)^10,40^. We further found that de novo heteroplasmies among children born to mothers aged under 30 years showed decreased pathogenicity as compared to those among children born to older mothers (*P* = 0.075, Fig. 6c; *P* = 0.043 for nonsynonymous heteroplasmies in OXPHOS genes). These results thus point to a germline or prenatal origin for these maternal-age-associated mtDNA changes.

### Sex differences in the association of mtDNA heteroplasmies and inter-generational transmission with ASD

Interestingly, the weakened effect of purifying selection on maternal heteroplasmies in children with NDD relative to controls was most prominent among boys (versus controls: *I*^2^ = 89%, *P* for heterogeneity = 0.0021) but not among girls (versus controls: *I*^2^ = 0%, *P* for heterogeneity = 0.46) (Supplementary Table 14). Accordingly, we found that maternally inherited PP heteroplasmies conferred a risk for ASD only among boys (Meta-OR = 3.81, *P* = 0.0057, Supplementary Table 15) in the SSC and BBC. Likewise, in the BBC, we noted a greater impact of PP heteroplasmies on ADHD and/or other DD after focusing on inherited PP heteroplasmies in cord blood of boys (OR ≥ 3.90, Supplementary Table 16). We also noted slightly higher prevalence of PP heteroplasmies among girls with neurotypical development in both the SSC and BBC relative to boys with neurotypical development (chi-squared test, *P* = 0.25; Supplementary Table 17). These results suggest that, compared to males, females may be resilient to deleterious effects of PP heteroplasmies, supporting a female protective effect in ASD and other NDD. But due to smaller numbers of females with ASD/NDD in the current study, future studies are needed to verify our observation.

## Discussion

In the SSC and BBC, the impact of PP heteroplasmies on ASD was found to be similar (*P* for heterogeneity ≥0.33, Table 1 and Supplementary Table 18), despite racial and ethnic differences between these two cohorts. While the PAR of PP heteroplasmies estimated to be 2.9% for ASD is small, it is comparable to the PARs of damaging coding or noncoding variants in the nuclear genome^4–6^. Given that mtDNA is less than one-thousandth of the nuclear exome and five-millionths of the nuclear genome in size, our finding points towards an enrichment of risk variants for ASD in mtDNA, showing that mtDNA-encoded genes may play an important role in the pathogenesis of ASD.

PP heteroplasmies in OXPHOS genes and in RNA genes were of comparable influence on ASD (Supplementary Table 19), whose locations in mtDNA spanned 33 genes and affected all four mtDNA-encoded OXPHOS complexes among children with ASD in the current study (Supplementary Fig. 3). The heterogeneous locations of these heteroplasmies echo the varying deficiencies of OXPHOS complexes previously found in patients with ASD^15–21^.

Some confirmed mitochondrial disorder (MD)-causing mtDNA mutations^36^ were identified among children with ASD (Supplementary Fig. 3). m.3243A>G in *MT-TL1* (*tRNA*^*Leu[UUR]*^), the most frequent pathogenic mutation causing MELAS (mitochondrial encephalopathy, lactic acidosis, and stroke-like episodes)^41^, affected two children with ASD. Previous studies indicated that an intermediate level of m.3243A>G (fraction < 30%) may cause mild mitochondrial dysfunction in ASD^42,43^. Another tRNA mutation, m.8313G>A in *MT-TK* (*tRNA*^*Lys*^), which causes mtDNA deletion syndrome and mitochondrial neurogastrointestinal disorder with progressive mental regression in early childhood^44^, was observed in three children with ASD. Moreover, we identified five MD-causing mutations (m.3460G>A, m.10663T>C, m.11778G>A, m.13042G>A, and m.13513G>A) in genes of OXPHOS complex I among children with ASD. All these mutations are responsible for LHON (Leber hereditary optic neuropathy)^45^, some of which also result in MELAS and/or Leigh disease^46^, a progressive, childhood-onset neurometabolic disorder. A mouse mtDNA model of LHON was recently shown to express impaired social interactions and increased repetitive behaviors^14^. Surprisingly, mitochondrial dysfunction and abnormal electroencephalogram were found to be most pronounced in the cortex and hippocampus of affected mice^14^, suggesting that mtDNA defects may have systemic impact on the brain and on neuropsychiatric functions. Overall, we found that carrying PP heteroplasmies that have been reported to be associated with any disease^36,47^ increased the risk of ASD by 88% (OR = 1.88, *P* = 0.0096), and more than doubled ASD risk (OR = 2.78, *P* = 0.0022) if the associated disease has a neurological manifestation (Supplementary Table 19).

Among children with ASD carrying PP heteroplasmies, over 90% had only one PP heteroplasmy detected (Supplementary Fig. 1), which is contradictory to an age-related burden of low-fraction heteroplasmies in somatic tissues^48^. We also found increased effects of PP heteroplasmies of medium-to-high fractions on ASD (Table 1), reflecting a threshold effect of heteroplasmies. But a clinically significant threshold for heteroplasmies detected in blood of patients with ASD may be lower than the threshold for classic mitochondrial disorders such as MELAS^41^, supporting mild mitochondrial dysfunction in ASD^49^. Because previous studies showed reduced mtDNA heteroplasmic fraction in blood^41^, low-fraction pathogenic heteroplasmies detected in the blood of the child, especially maternally inherited heteroplasmies, may warrant evaluation of the heteroplasmy in non-blood tissues.

In the SSC and BBC, neither mtDNA mutation rate nor the pattern of nucleotide changes among de novo heteroplasmies significantly differed between affected children and controls (Supplementary Tables 12 and 20). A lack of change in mtDNA mutation rate in ASD rules out the possibility of heightened mtDNA mutagenesis due to oxidation of mtDNA polymerase γ^50^. A minimal proportion of transversion changes among de novo heteroplasmies (≤3.1%, Supplementary Table 20) further contradicts the signature of mutations created by misincorporation of bases against mtDNA damages^48^.

Besides increased incidence, de novo mtDNA heteroplasmies among children born to older mothers showed elevated pathogenicity. Recent studies indicated that most de novo germline mutations in mtDNA arise during oocyte meiotic arrest^10,51^. Oocytes from aged mice showed impaired mitochondrial fission affecting embryonic development^52^. In humans, ovarian aging was associated with decreased mtDNA content in unfertilized oocytes^53^. These age-related mitochondrial changes may affect selective removal of deleterious mtDNA mutations^54^ during subsequent maturation of the oocyte and development of the embryo^55,56^.

In the SSC, we observed that high mtDNA content in peripheral blood samples mitigated the deleterious effects of PP heteroplasmies on ASD. Similar modifying effects of high mtDNA content were also observed in MD, leading to incomplete penetrance of MD-causing mtDNA mutations and reduced disease severity^41,57^. A recent study showed that the protective role of increased blood mtDNA content in neurological disorders may be ascribed to its connection with global gene expression beyond the immune system^58^. Functional assessment using fibroblasts and postmortem specimens of patients with LHON and asymptomatic carriers showed that increased mtDNA content in blood was attributed to a greater capacity of activating mitochondrial biogenesis globally, which better ameliorated some pathogenic phenotypes of the causative mtDNA mutation in the optic nerve^57^. In Picard et al.’s study^42^, cybrid cells with an intermediate level of m.3243A>G exhibited dynamic capacity of mtDNA to adjust to mild mitochondrial dysfunction via changes in its content and gene expression. However, none of the ASD risk genes in the nuclear genome^3^ are directly implicated in the pathway of mitochondrial biogenesis. Future studies are needed to identify potential nuclear modifiers for diseases associated with pathogenic mtDNA variants.

Interestingly, the deleterious effect of an mtDNA variant may be modulated by sex hormones such as estrogens^59,60^. Receptors of estrogens (ER), such as Erβ, have been shown to be localized to mitochondria and bind mtDNA^61^. In the brain of children with autism, the levels of Erβ, estrogen synthetase, and ER co-activators were significantly decreased relative to controls^62^. Treatments using estrogens have been shown to have neuroprotective effects against mtDNA defects in LHON^59,60^ and, more recently, against nuclear genetic defects in autism^63^.

From an evolutionary perspective, a female protective effect may dampen purifying selection and cause accumulation of deleterious mtDNA variants in females. They may lead to the associated disease when transmitted to a male offspring, illustrating a contribution of the “mother’s curse” to a male-dominant disease like LHON^64^. Future studies may be needed to examine how estrogens and other sex-related factors impact inter-generational transmission and selection of mtDNA heteroplasmies and their roles in sexual dimorphism in NDD.

Our study has some limitations. First, we used DNA samples from peripheral blood collected in the SSC and cord blood collected in the BBC rather than brain tissue. It is uncertain to what extent blood mtDNA can reflect mtDNA in brain tissue. In Li et al.’s study^65^, among 62 young and middle-aged study participants (age < 55 years) all 26 mtDNA heteroplasmies, detected with VAF ≥ 5% in blood, were shared among more than eight postmortem tissues of the same participant, including the cerebellum, cerebrum, and cortex of the brain. Our findings thus lend support for the utility of blood mtDNA as a biomarker of ASD risk. Second, although our study utilized two large population samples, our analyses may still be underpowered due to the relatively low frequencies of mtDNA heteroplasmies and small numbers of females with ASD in the SSC and BBC. Third, different methods were used to sequence mtDNA in the SSC and BBC. Remarkably, the findings from the two cohorts were consistent. This study thus demonstrates the utility of STAMP^33^ as a cost-effective tool for studying mtDNA heteroplasmies and content in large population samples. Fourth, our study on mtDNA heteroplasmies and content in ADHD and other DD was exploratory among a relatively small number of affected children only from the BBC. The related results suggest that mtDNA variations may have a broader impact on other NDD besides ASD at childhood, which requires further research.

In conclusion, this study analyzed two independent and racially and ethnically diverse cohorts, and revealed consistent findings regarding the role of mitochondrial genome quality (mtDNA heteroplasmies) and quantity (mtDNA content), germline selection, advanced maternal age, and child sex and their inter-relationships with the risk of ASD specifically and NDD in general. In the BBC, we showed that mtDNA variations measured at birth are prospectively associated with a child’s risk of developing ASD in childhood. These findings, if further confirmed, indicate the utility of maternal peripheral blood and cord blood mtDNA biomarkers as identified through ultradeep sequencing for the early risk assessment and prediction of a newborn’s future risk of developing ASD, and perhaps, other NDD, in childhood.

## Methods

### Processing of whole-genome sequencing data in the SSC

The details of the SSC were previously described^26^. We downloaded the files containing whole-genome sequencing (WGS) data (aligned to the complete human genome; genome assembly GRCh38; last access in June 2019) of 7768 participants from 1942 families recruited in the Simons Simplex Collection (SSC) after receiving data access from the SFARI base (https://base.sfari.org/). We used an established method^22^ to analyze mtDNA variations (Supplementary Table 21). We first extracted paired-end reads mapped to the mitochondrial genome (mtDNA) in the alignment files using samtools (v1.6)^66^. We then aligned these reads, using “bwa mem” (v0.7.17)^67^, to a modified mtDNA, which have the final 150-bp of the Revised Cambridge Reference Sequence (rCRS) of human mtDNA copied to the start to allow for a complete alignment of reads in the D-loop region in mtDNA. We further performed local re-alignment with freebayes (v1.1)^68^ and base quality recalibration with samtools (calmd)^66^. The resulting bam files were converted to the corresponding pileup files using “samtools mpileup” for variant calling.

mtDNA variants were called using unique reads identified by “picard MarkDuplicates” (v1.138)^22^ that (i) were mapped in a proper pair to mtDNA except for reads in the circular D-loop region; (ii) had mapping quality (MAPQ) ≥ 20 and base alignment quality (BAQ) ≥ 30; (iii) contained fewer than 5% nucleotide mismatches relative to rCRS (Supplementary Table 21). mtDNA heteroplasmies were retained for analysis if (iv) having >500-fold (X) depth of unique reads with >70% of the bases having BAQ ≥ 30; (v) not in low-complexity regions (m.302-m.316, m.512-m.526, m.16184-m.16193); (vi) having ≥5 minor alleles and log-likelihood quality score >5 computed based on BAQ scores^22^; and (vii) showing nonzero and comparable allele fractions detected among unique reads mapped to the forward strand and to the reverse strand of mtDNA (Fisher’s exact test *P* ≥ 10^−4^) (Supplementary Table 23).

We replicated >99.98% (83,750/83,760) of mtDNA homoplasmies identified using the whole-exome sequencing (WES) data from 810 families from the SSC in our previous study^22^. Of mtDNA heteroplasmies previously reported^22^, 95.1% (545/573) were confirmed using the WGS data from the SSC at comparable variant allele fractions (VAF ≥ 1.5%) (Pearson’s *r* = 0.98 and Spearman’s *ρ* = 0.94, *P* < 2.2 × 10^−16^; Supplementary Fig. 4), among which 93.4% (509/545) were of medium-to-high fractions (VAF ≥ 5%) in both studies. mtDNA haplogroup information was determined using haplogrep2 (v2.1.1)^69^ based on the major mtDNA sequence (with VAF > 50%) of each sample. Supporting the maternal transmission of mtDNA, all major mtDNA sequences of participants in the same maternal lineage were classified into identical mtDNA haplogroups. Among parents in the SSC, 82% had mtDNA belonging to European macro-haplogroups; 12% had Asian or Native American macro-haplogroups; and 5% had African macro-haplogroups. Ancestry information inferred from mtDNA macro-haplogroups also corresponded to self-reported races or ethnicities (Supplementary Fig. 4).

We estimated the number of mtDNA copies in relation to the nuclear DNA by using the ratio of sequencing depth between mtDNA and nuclear DNA (Supplementary Table 21). In brief, we calculated sequencing depth at each site of the reference nuclear genome by using the “samtools depth -a” command^66^ after filtering out read duplicates, reads not in a proper pair, reads with low MAPQ scores (<20), and read bases with low BAQ scores (<16). To avoid regions in nuclear DNA with large deletions or ambiguous alignments, we computed average sequencing depth in sliding windows of 100-kb with step size of 50-kb. Average read coverage on each chromosome was estimated based on windows with >80% sites covered with reads. mtDNA content was then computed as two times the average ratio of read coverage between mtDNA and each of the 22 autosomal chromosomes.

At the individual level, we removed families with participants having (i) excessive (*n* > 15) de novo heteroplasmies (*n* = 2); (ii) mismatched mtDNA haplogroup information between the mother and child (*n* = 0); and (iii) medium sequencing coverage on mtDNA <500X (*n* = 0). Two families were excluded from analysis per request to withdraw from the SSC.

Phenotypic data, including age, sex, reported ancestry of all participants, as well as verbal IQ, non-verbal IQ, scores of the VABS and ADOS, and medical records of the probands were extracted from the SFARI phenotype dataset (version 15; https://base.sfari.org/) and the related datasets on NDAR (collections: 2042, 2068; https://nda.nih.gov/). Nuclear risk factors for ASD reported using the SSC, including polygenic risk scores of ASD-associated common variants^1,70^, de novo coding mutations^4^, de novo mutations in the noncoding regions of the nuclear genome^6^, and de novo structural variants^5^, were obtained from previous studies of the SSC. The related methods were detailed in Supplementary Table 2.

Our research using only pre-existing de-identified data in the SSC was exempted from Institutional Review Board (IRB) review (Protocol ID# 1703007002) for human subject research by the IRB office at Cornell University prior to the current study.

### Processing of mitochondrial genome sequencing data in the BBC

The details of the study design, recruitment and follow-up of study participants, and ascertainment of diseases in the BBC have been published^28,29,71^ and were described in Supplementary Notes. We followed a previous protocol^33,72^ in preparing STAMP sequencing libraries and processing paired-end reads for BBC samples. Briefly, we captured the entire 16.6-kb mtDNA sequence with 46 pairs of oligonucleotide probes targeting mtDNA and five pairs of probes targeting independent, single-copy regions on five autosomal chromosomes (Integrated DNA Technologies; probe sequences are in Supplementary Table 24)^33^. We performed the hybridization reaction on 50 ng genomic DNA with 4 µl STAMP probe mix and 1× Ampligase buffer (Epicentre, catalog #A1905B) in a 10 µl volume. Thermal conditions were 10 min at 95 °C for denaturation, followed by a decrease of 1 °C per min to 55 °C and 20 h at 55 °C for hybridization. We then added 6 µl gap-filling mix containing 0.1 mM dNTPs, 0.6 M Betaine, 0.1 M (NH_4_)_2_SO_4_, 0.5 units of *Tsp* DNA polymerase (Invitrogen, catalog #11448024), and 0.5 units of Ampligase (Epicentre, catalog #A3202K) in 1× Ampligase buffer and incubated the mix at 55 °C for another 20 h for gap filling. We amplified 1.5 µl of the capture product in a 50 µl PCR reaction with 0.5 µM of p5i5 and p7i7 indexing primers containing sample barcodes (Supplementary Table 24), 0.2 mM dNTP, 1 × Phusion HF buffer, and 1 unit of Phusion Hot-Start II DNA polymerase (Thermo Scientific, catalog #F549L). PCR thermal conditions were 30 s at 98 °C for initial denaturation, followed by 25 cycles of 10 s at 98 °C, 15 s at 65 °C, and 15 s at 72 °C. We purified STAMP sequencing libraries with AMPure XP magnetic beads (Beckman Coulter, catalog #A63881) and sequenced them on HiSeq 2500 (Illumina) with 2 × 250-bp reads.

Paired-end reads obtained were analyzed using “stamp toolkit”^33^ (Supplementary Table 22). We first sorted paired-end reads into clusters of capture products according to the probe arm sequences and sample barcodes identified. We then trimmed molecular barcode and probe sequences from each read pair before alignment. Following a similar mapping strategy used for the WGS data from the SSC, we aligned the resulting reads, using “bwa mem” (v0.7.17)^67^, to the complete human genome (genome assembly GRCh38) and subsequently to a modified mtDNA sequence based on rCRS. Paired-end reads aligned to the target region as specified by their arm sequences were locally realigned using freebayes (v1.1.0)^68^; and their base qualities were recalibrated using samtools (calmd)^66^. For paired-end reads with the same molecular barcode, we employed a Bayesian approach to merge base information at each alignment site and generated a consensus read representing the captured DNA product^33^.

mtDNA variants were identified using consensus reads aligned to mtDNA that (i) had MAPQ ≥ 20 and BAQ ≥ 30; (ii) did not contain an excess of nucleotide mismatches (>5 in the coding region and >8 in the D-loop region) relative to the major mtDNA sequence; and (iii) were not marked as nuclear mitochondrial segments^72^ (Supplementary Table 22). We determined heteroplasmies based on similar quality filters used for the WGS data: (iii) >500X depth of coverage with >70% of the bases having BAQ ≥ 30; and (iv) not in low-complexity regions; (v) ≥5 minor alleles and log-likelihood, BAQ-based quality score >5; and two other filters based on consensus read information in STAMP^33^: (vi) VAF among consensus reads constructed from multiple paired-end reads comparable to that among consensus reads constructed from single paired-end reads (Fisher’s exact test *P* ≥ 10^−4^ and decrease in VAF < 5-fold); and (vii) not at low-quality sites defined as those having >50% of variants failed in quality filter vi (Supplementary Table 23).

We estimated, based on 102 pairs of technical replicates, that the true positive rate was 100% (5,003/5,003) for detecting homoplasmies and was 98.6% (70/71) for detecting heteroplasmies (correlation in VAF: *r* > 0.99 and *ρ* = 0.96, *P* < 2.2 × 10^−16^; Supplementary Fig. 4); the average coefficient of variation in repeated measures of VAF was 7.6% among heteroplasmies of VAF ≥ 5% and was 19.2% among heteroplasmies of VAF between 1.5% and 5%. In the BBC, 69% of participants showed mtDNA macro-haplogroups of African ancestry, while Asian or Native American macro-haplogroups (19%) and European macro-haplogroups (12%) were minorities, in line with self-reported races or ethnicities (Supplementary Fig. 4).

We used a residual method to compute mtDNA content from the STAMP data^33,72^ based on the number of consensus reads from mtDNA and the number of consensus reads from nuclear DNA (Supplementary Table 22). To avoid variation in capture efficiency due to mtDNA polymorphisms in probe-annealing regions^33,72^, consensus reads used were from six target regions of probes whose arm sequences did not overlap with mtDNA polymorphisms having the minor allele detected in ≥5 (frequency > 0.2%) BBC samples. Consensus reads representing nuclear DNA were from probe-target regions as previously described^33,72^. We found a correlation of mtDNA content at *r* = 0.83 and *ρ* = 0.82 (*P* < 2.2 × 10^−16^) among 102 pairs of technical replicates.

In total, we sequenced mtDNA in samples from 1067 mother-child pairs in the BBC. We found that 21 (2%) showed a discordant mtDNA haplogroup with the major allele differing at ≥5 mtDNA sites between the mother and child. Given the known cross-generational mutation rate of mtDNA, we attributed these mismatches to potential mislabeling errors. Accordingly, at the individual level, we removed mother-child pairs with participants having (i) mislabeling errors (*n* = 21); (ii) excessive (*n* > 15) de novo heteroplasmies (*n* = 6); (iii) median coverage of consensus reads on mtDNA <500X (*n* = 36); (iv) no reads from nuclear DNA to compute mtDNA content (*n* = 3); and (iv) incomplete data on sex or disease phenotypes (*n* = 4). As a result, we retained 997 mother-child pairs (93.4%) for studying mtDNA heteroplasmies and content in the BBC. Demographic data were collected using a standardized questionnaire in the BBC. Clinical information on the children was extracted from their medical records containing the International Classification of Diseases (ICD) codes (last access in December 2020), which were derived from physicians’ primary and secondary diagnoses at the Boston Medical Center.

### Detection power of mtDNA heteroplasmies

Among mtDNA reads retained for heteroplasmy detection, variant alleles occurred at a rate of about 0.02% per base, which is well below the probability of errors (0.1% per base) specificized by the minimum threshold of BAQ (≥30). Assuming an extreme scenario in which all variant alleles result from sequencing or alignment errors, we estimated that 2,000 unique reads from WGS or STAMP would still guarantee >99.9% power to discriminate real heteroplasmies of VAF = 1% from errors at all sites of mtDNA (alpha = 0.05/16569). When applied to one specific mtDNA site (alpha = 0.05), the ultradeep mtDNA sequencing data used in the current study would ensure 90% power to identify true heteroplasmies at VAF as low as 0.2% (Supplementary Fig. 5). Low-fraction heteroplasmies might be contaminated by variants from nuclear mitochondrial segments (NUMTS). In the STAMP data, these variants were filtered out using both alignment and consensus read methods as previously described^33,72^. In the WGS data, we assessed fraction distributions of variants associated with polymorphic NUMTS as well as inferred from mtDNA content (Supplementary Notes). We found that using a minimum VAF at 1.5% to define mtDNA heteroplasmies could remove >99% spurious variants associated with NUMTS (Supplementary Fig. 6). We further provided the results from analyses using only medium-to-fraction heteroplasmies (VAF ≥ 5%) as sensitivity testing.

We determined that a heteroplasmy is shared in a pair of samples (e.g., mother-child pair) if the variant allele can be detected in both samples with fraction ≥0.2% among >500 unique reads and the probability of observing more errors than the variant allele is <10^−3^ (Supplementary Table 23). By using this criterion to assess heteroplasmy transmission in mother-child pairs, we found clear separation of variant quality scores between de novo and inherited heteroplasmies, as well as between transmitted and untransmitted heteroplasmies (Supplementary Fig. 5). The probability of heteroplasmy sharing among mother-child pairs was at least 15 times higher than that among father-child pairs and among father-mother pairs (<3.4%; Supplementary Fig. 5). None of the predicted pathogenic heteroplasmies identified in probands, siblings and/or mothers were shared with the father from the same family in the SSC (Supplementary Data 1).

In total, we identified 7202 heteroplasmies, including 724 secondary heteroplasmies detected based on heteroplasmy sharing among family members in the SSC or mother-child pairs in the BBC (Supplementary Table 23). We defined mtDNA heteroplasmies with VAF ≥ 1.5% in children that were not shared with participants in the same maternal lineage as de novo heteroplasmies.

### Bioinformatics analysis of mtDNA variation

We annotated mtDNA variants’ function by using the ANNOVAR pipeline^73^. Consistent with our previous study of the SSC^22^, we relied on multiple pathogenicity predictors to assess the functional impact of nonsynonymous heteroplasmies in OXPHOS genes, including (i) CADD^74^ Phred score (version 1.3) >15, (ii) PolyPhen-2 of “possibly or probably damaging”^75^, and (iii) MutPred^76^ score >0.6. Moreover, we used (iv) MitoTIP^77^ raw score >12.66 as recommended to predict the pathogenic potential of nucleotide changes in mtDNA-encoded tRNAs. We furthermore evaluated pathogenicity and population frequency of all mtDNA variants in OXPHOS genes and tRNA genes (Supplementary Notes and Supplementary Fig. 7). Predicted pathogenic (PP) variants were then defined as those that (1) are predicted to be pathogenic in pathogenicity assessments i–iv, or (2) are confirmed as mitochondrial disorder (MD)-causing mutations in the MITOMAP database^36^ but are not marked as “benign” in the ClinVar database^47^ (last access in April 2021), and (3) have a population frequency of the variant allele ≤0.05% among apparently healthy participants in HmtDB^34^ (the Human Mitochondrial Database, 2020 update) and among reference populations in gnomAD^35^ (the Genome Aggregation Database, version 3.1.1) (Supplementary Table 23).

Moreover, we converted CADD^74^ and MitoTIP^77^ raw scores, by using rank-based inverse normal transformation, to normally distributed z-scores that represent the pathogenicity rank of each variant among all possible nucleotide changes in OXPHOS genes and tRNA genes, respectively. We then used two methods to test the existence of selection of mtDNA heteroplasmies. First, we used a one-sample *t* test to assess whether the mean pathogenicity z-score of a set of heteroplasmies is significantly deviated from the medium pathogenicity. Second, we employed a bootstrapping-based test that compares the mean pathogenicity z-score to those from 10^5^ random resamples of nucleotide changes that match the number of changes in OXPHOS genes and tRNA genes as well as the number of transition and transversion changes among the set of heteroplasmies observed. The resulting *P* values are denoted as “*P* for selection”.

### Statistical methods

We assessed mitochondrial exposures for NDD, including the number of mtDNA heteroplasmies (heteroplasmy incidence), a binary variable representing carrying PP heteroplasmies, and the standardized mtDNA content z-score (mtCNz). We considered a child carrying PP heteroplasmies in the SSC if the fraction of the heteroplasmy was higher than that in the sibling. As paired sibling data was unavailable in the BBC, we considered a child carrying PP heteroplasmies if the heteroplasmy increased in fraction relative to that of the mother. We then computed the family-adjusted prevalence of PP heteroplasmies accordingly. We standardized mtDNA content in peripheral blood from children in the SSC with adjustment for age at the time of sample collection and the parental mean of mtDNA content, and standardized mtDNA content in cord blood from children in the BBC with adjustment for gestational age and maternal mtDNA content. Among children whose age information was unavailable (215 siblings in the SSC), we adjusted mtDNA content with the mean age of the respective group.

In the SSC, we used conditional logistic regression to assess the association of ASD with mitochondrial exposures among matched probands and siblings within SSC families:

1 $${{{{{\rm{logit}}}}}}({{{{{\rm{ASD}}}}}})\sim {{{{{\rm{mitochondrial}}}}}}\,{{{{{\rm{exposure}}}}}}({{{{{\rm{s}}}}}})+{{{{{\rm{strata}}}}}}({{{{{\rm{family}}}}}}\,{{{{{\rm{id}}}}}})$$

In the BBC, we employed logistic regression to assess the association of NDD with mitochondrial exposures among unrelated children:

2 $${{{{{\rm{logit}}}}}}({{{{{\rm{NDD}}}}}})\sim {{{{{\rm{mitochondrial}}}}}}\,{{{{{\rm{exposure}}}}}}({{{{{\rm{s}}}}}})+{{{{{\rm{sex}}}}}}+{{{{{\rm{mtDNA}}}}}}\,{{{{{\rm{inferred}}}}}}\,{{{{{\rm{ancestry}}}}}}$$

We also performed sex- or race-stratified analyses of mitochondrial exposures in relation to ASD or NDD in the SSC and BBC. The population-attributable risk proportion (PAR)^78^ was computed based on the family-adjusted prevalence of PP heteroplasmies among controls and the combined effect estimated from the SSC and BBC using fixed-effect meta-analysis^79^. The 95% confidence interval of PAR was estimated based on 1000 bootstrap resamples of families from the SSC as well as mother-child pairs from the BBC. Transmission of mtDNA heteroplasmies in relation to pathogenicity of maternal heteroplasmies was assessed in logistic regression as:

3 $${{{{{\rm{logit}}}}}}({{{{{\rm{transmitted}}}}}})\sim {{{{{\rm{pathogenicity}}}}}}\,{{{{{\rm{z}}}}}}\,{{{{{\rm{score}}}}}}	+{{{{{\rm{RNA}}}}}}\,{{{{{\rm{or}}}}}}\,{{{{{\rm{OXPHOS}}}}}}\\ 	+{{{{{\rm{maternal}}}}}}\,{{{{{\rm{VAF}}}}}}+{{{{{\rm{sex}}}}}}$$

Age-dependent changes in pathogenicity of de novo mtDNA heteroplasmies in children was examined in linear regression as:

4 $$\begin{array}{c}{{{{{\rm{pathogenicity}}}}}}\,{{{{{\rm{z}}}}}}\,{{{{{\rm{score}}}}}}\sim {{{{{\rm{maternal}}}}}}\,{{{{{\rm{age}}}}}}\,{{{{{\rm{groups}}}}}}+{{{{{\rm{RNA}}}}}}\,{{{{{\rm{or}}}}}}\,{{{{{\rm{OXPHOS}}}}}}\\ \,+\,{{{{{\rm{VAF}}}}}}+{{{{{\rm{sex}}}}}}+({{{{{\rm{disease}}}}}}\,{{{{{\rm{status}}}}}}\,{{{{{\rm{if}}}}}}\,{{{{{\rm{tested}}}}}}\,{{{{{\rm{using}}}}}}\,{{{{{\rm{all}}}}}}\,{{{{{\rm{samples}}}}}})\end{array}$$

Among children with ASD in the SSC, we examined the associations of carrying PP heteroplasmies with ASD-related traits by using logistic or linear regression with control for age at the time of sample collection, sex, and mtDNA-inferred ancestry. Paternal and maternal ages at childbirth were further considered as additional covariates in the tests of disease status and ASD-related traits (i.e., model 2 in Table 1 and Supplementary Tables 8 and 9). Among children whose paternal or maternal age information was unavailable (5 probands and 215 siblings in the SSC), we imputed data with the mean value of the respective group for covariate adjustment.

Other statistical methods and models used are indicated in the main text. All tests were performed using R (version 3.5.0). The R package survival (version 2.41-3) was used for conditional logistic regression (the clogit function). Box plots were generated using the box-plot function in R. Nominal *P* values from two-sided tests are reported.

### Reporting summary

Further information on research design is available in the Nature Research Reporting Summary linked to this article.

## Supplementary information

Supplementary Information

Description of Additional Supplementary Files

Supplementary Data 1

Reporting Summary

## Data Availability

The data on mtDNA heteroplasmies of the SSC generated in this study are provided in the Supplementary data file (Supplementary Data 1). The raw whole-genome sequencing data and phenotype data of the SSC used in this study are available in the SFARI Base (https://base.sfari.org/) under Resource: SSC Whole-genome 2 and Simons Simplex Collection. The related phenotype data of the SSC are also available in NADR database under collections 2042 (https://nda.nih.gov/edit_collection.html?id=2042) and 2068 (https://nda.nih.gov/edit_collection.html?id=2068). The data of the BBC are only available under restricted access due to the informed consent and Institutional Review Board (IRB) guidance of the BBC which is an ongoing birth cohort study with study participants still being under follow-up. All the study participants signed informed consent form; and the study protocol and its data and analyses are under the oversight of two IRBs: Johns Hopkins University and Boston Medical Center. Access to the BBC data (including the raw mtDNA sequencing data, the phenotype data used, and the data on mtDNA generated in this study) can be obtained from the corresponding author, Dr. Xiaobin Wang (email: xwang82@jhu.edu), after the data access request and a study proposal related to mtDNA and neurodevelopmental disorders are reviewed and approved by the two IRBs. A download link and access for the BBC data will be provided shortly after approval.

## Code availability

The computational pipeline developed to process mtDNA reads and call mtDNA variations in the SSC and BBC are available on GitHub under the repository “stamp toolkit” (https://github.com/mtstamp/stamp).
