# Supplementary material for: Association of mitochondrial DNA content, heteroplasmies and inter-generational transmission with autism
Source: Nat Commun. 2022 Jul 1;13:3790. doi: 10.1038/s41467-022-30805-7 (PMC9249801; doi:10.1038/s41467-022-30805-7)
Supplement: Supplementary file 1 — Supplementary Information [file 41467_2022_30805_MOESM1_ESM.pdf]

**Association of Mitochondrial DNA Content, Heteroplasmies and Inter-generational Transmission with Autism**

Yiqin Wang[1], Xiaoxian Guo[1], Xiumei Hong[2], Guoying Wang[2], Colleen Pearson[3], Barry Zuckerman[3], Andrew G Clark[4,5], Kimberly O O'Brien[1], Xiaobin Wang[2, 6]*, Zhenglong Gu[1,7]*

**Affiliations:**

1. Division of Nutritional Sciences, Cornell University, Ithaca, New York, USA.
2. Center on Early Life Origins of Disease, Department of Population, Family and Reproductive Health, Johns Hopkins University Bloomberg School of Public Health, Baltimore, Maryland, USA.
3. Department of Pediatrics, Boston University School of Medicine and Boston Medical Center, Boston, Massachusetts, USA.
4. Department of Molecular Biology and Genetics, Cornell University, Ithaca, New York, USA.
5. Department of Computational Biology, Cornell University, Ithaca, New York, USA.
6. Department of Pediatrics, Johns Hopkins University School of Medicine, Baltimore, Maryland, USA.
7. The author is now affiliated with Center for Mitochondrial Genetics and Medicine, Greater Bay Area Institute of Precision Medicine (Guangzhou), Fudan University, Guangzhou, China.

*These authors are co-corresponding authors.

# Supplemental information

## Contents

**Supplementary Notes** ..... 2

**1. Boston Birth Cohort** ..... 2

**2. Complications of variants from nuclear mitochondrial segments** ..... 3

**3. mtDNA variant pathogenicity and frequency in the general population** ..... 4

**Supplementary Figures** ..... 8

**Supplementary Tables** ..... 19

**Supplementary References** ..... 38

# Supplementary Notes

## 1. Boston Birth Cohort

The Boston Birth Cohort (BBC)[1–3] is a predominantly urban low-income, population of mostly African Americans and Latino Americans, recruited at the Boston Medical Center (BMC), in Boston, MA. After giving written informed consent, mothers were asked to answer a standardized questionnaire on socio-demographics, lifestyles, and medical history. Birth outcomes and other clinical information on the mother and the infant were obtained from the medical records. Pregnancies due to multiple gestations or in vitro fertilization, maternal trauma related to childbirth, and infants with major birth defects or chromosomal aneuploidy were excluded from the study. The study aim of the BBC at the enrollment stage was to identify risk factors for preterm (<37 weeks) and low birthweight (<2,500 g) births. Children receiving postnatal care at BMC were followed. The study was approved by the Institutional Review Boards of Boston Medical Center (IRB Number: H-23237) and Johns Hopkins Bloomberg School of Public Health (IRB Number: 3966/CR513).

Disease status was defined according to the children's electronic medical records containing International Classification of Diseases (ICD) codes, which were derived from physicians' primary and secondary diagnoses from all visits at BMC. The group with autism spectrum disorder (ASD) consisted of children who were ever diagnosed with autism (ICD-9 code: 299.00), Asperger syndrome (299.80), and/or pervasive developmental disorder not otherwise specified (299.90). The ASD diagnoses were mostly obtained from pediatricians and relevant specialists, and all were then reviewed by highly trained staff at BMC's autism evaluation program. The attention-deficit/hyperactivity disorder (ADHD) group consisted of children ever diagnosed with ADHD (314.0–314.9). The developmental delay (DD) and other neurodevelopmental disorders (NDD) group was comprised of children who were ever diagnosed with developmental delay (DD), including language delay, coordination disorders, or learning disorders (315.0–315.5), developmental delay not elsewhere classified (315.8), tuberous sclerosis with developmental delay (316 and 759.5), intellectual disability (317, 318.0–318.2, and 319), and any other mental, behavioral, and neurodevelopmental disorders (290-319) apart from ASD, ADHD, and DD. Children who were not assigned to any of the aforementioned groups of NDD cases constituted the neurotypical (NT) group.

Genomic DNA was exacted from newborn cord blood and maternal peripheral blood collected 24-72 hours postpartum. Quantification of genomic DNA was previously described in a study of the BBC using the Illumina human genotyping array[4]. A total of 1067 mother-child pairs, a subset of the BBC, were included in the current study. After performing quality filtering on the mitochondrial genome (mtDNA) sequencing data, we retained 997 mother-child pairs for the association analyses of mtDNA heteroplasmies and content with NDD. These 997 children included 621 children in the NDD groups and 376 children in the NT group. Among the 621

children with NDD, 82 had ASD, 259 had ADHD, and 542 had DD or other NDD (**Supplementary Figure 2a**). We defined three mutually exclusive NDD groups as the case groups in the BBC: the 82 children with ASD constituted the "ASD" group; 221 children with ADHD without ASD constituted the "ADHD not ASD" group; the remaining 318 children with NDD constituted the "DD/other only" group (**Supplementary Figure 2b**). The median length of postnatal follow-up of children in the current study was 9.9 years (interquartile range: 6.9-12.9 years) and was 10.4 years (interquartile range: 7.5-13.3 years) among children diagnosed with NDD. Assuming a 6% event rate of predicted pathogenic (PP) mtDNA heteroplasmies among NT children, we estimated that the sample sizes in the NDD groups and the NT group guarantee 80% power to identify an increase in PP heteroplasmies (one-sided alpha=0.05) at an OR (odds ratio) =1.8 for any NDD, OR=2.7 for ASD, OR=2.1 for ADHD not ASD, and OR=2.0 for DD/other only.

## 2. Complications of variants from nuclear mitochondrial segments

Besides sequencing errors, alternate alleles among reads mapped to mtDNA may arise from variants in nuclear mitochondrial segments (NUMTS) that are not correctly annotated in the reference human genome, such as those found to be polymorphic in the general population[5]. Since NUMTS segregate with nuclear DNA instead of mtDNA, sequences of NUMTS would be associated with certain nuclear DNA haplotypes. If such haplotypes are captured by mtDNA probes designed in STAMP, they would result in spurious mtDNA heteroplasmies whose minor alleles are shared among samples with different mtDNA haplogroups. We followed a similar strategy to identify and filter out spurious heteroplasmies from polymorphic NUMTS captured by mtDNA probes in STAMP, as in our previous study on 362 blood samples from individuals of European ancestry[6]. In the BBC, we found 47 common minor-allele-containing haplotypes (>1% of BBC samples [n≥20]; **Supplementary Figure 6a**) captured by mtDNA probes. Among them, 33 corresponded to known NUMTS sequences already annotated in the reference nuclear genome (genome assembly GRCh38), and 12 haplotypes overlapped with reported polymorphic NUMTS sequences or those identified in our previous study using STAMP[5–7]. The remaining two potential haplotypes of NUMTS (frequency = 2.4%) showed six variant alleles (m.3399A>G, m.3594C>T, m.3915G>A, m.3918G>A, m.4002C>T, and m.4096C>T) and were captured by probes "A9" or "A10" in STAMP. All these variant alleles affect gene *ND1* but do not change its protein sequence (synonymous variants). We then added the sequences of these 47 haplotypes to the list of NUMTS sequences to annotate consensus reads of BBC samples. We found that the proportion of consensus reads marked as NUMTS was negatively correlated with mtDNA content estimated using the STAMP data of the BBC (Pearson's $r$=-0.42; Spearman's $\rho$=-0.47; $P<2.2\times10^{-16}$; **Supplementary Figure 6b**), supporting the nuclear DNA origin of these reads.

Among common minor-allele-containing haplotypes that have not been annotated in the reference nuclear genome (genome assembly GRCh38), the most abundant one (frequency=60%; **Supplementary Figure 6a**) was captured by probe "C11", which contains two discriminating variant alleles: m.12684G>A and m.12705C>T (synonymous variants of *ND5*). These two alleles were also found to be prevalent among paired-end reads aligned to mtDNA in the whole-genome sequencing (WGS) data of the Simons Simplex Collection (SSC). As expected, the observed allele fractions of heteroplasmies at these two mtDNA sites were in inverse proportion to mtDNA content computed using the WGS data from the SSC ($r \leq -0.22$; $\rho \leq -0.18$; $P < 2.2 \times 10^{-16}$; **Supplementary Figure 6c**). The distributions of the fractions of these two alleles span a large range with the bottom 30% falling below the detection threshold at variant allele fraction (VAF) of 0.1% (**Supplementary Figure 6d**), suggesting that at least 30% of participants in the SSC may not carry this NUMTS.

Moreover, we found that the 99$^{th}$ percentile of the fraction distribution of m.12684G>A and m.12705C>T, estimated to be about 1.5%, was in concordance with that of the relative ratio of sequencing depth between nuclear DNA and mtDNA (=2/mtDNA content) (**Supplementary Figure 6e**), indicating that sequencing reads from polymorphic NUMTS mostly contaminate mtDNA heteroplasmies of low fractions in the SSC. Accordingly, we excluded all heteroplasmies identified at m.12684G>A and m.12705C>T from analysis, and chose 1.5% as the minimum cutoff for VAF as well as the maximum likelihood estimation of the minor allele fraction from the BAQ (base alignment quality)-based test[8] to determine heteroplasmic mtDNA sites among participants from the same family in the current study (**Supplementary Table 23**). We also presented the results based on a more rigid criterion that retains only mtDNA heteroplasmies of medium-to-high fractions (VAF≥5%).

We found that all remaining heteroplasmies in the coding region of mtDNA were of rare frequencies in the SSC (frequency ≤0.2% among 7,752 participants). We also observed minimum proportions of mtDNA heteroplasmies shared between the father and child as well as between the father and mother from the same family in the SSC (**Supplementary Figure 5 g** and **h**). These results indicate that the influence of contamination from NUMTS sequences on heteroplasmy detection would be limited after we filtered out heteroplasmic sites with VAF<1.5%.

### 3. mtDNA variant pathogenicity and frequency in the general population

We relied on CADD[9] and MitoTIP[10] to assess pathogenicity of mtDNA variants in oxidative phosphorylation (OXPHOS) genes and in tRNA genes, respectively. Both CADD and MitoTIP utilize an agglomerate of features of DNA and its encoded products to improve prediction accuracy. Distinct from MitoTIP[10], which is specifically designed for mtDNA-encoded tRNAs, CADD is a genome-wide pathogenicity predictor developed for all genes and functional

elements in the human genome[9]. Its recommended threshold for "likely pathogenic" at a CADD Phred score of 15 represents the top 3.2% ($=10^{15/10}$) in pathogenicity among all genome-wide variants[9]. In our previous study using the whole-exome sequencing (WES) data from the SSC[8], we found that CADD[9] Phred scores of missense variants in OXPHOS genes were strongly correlated with metrics of other commonly used genome-wide pathogenicity predictors, such as PolyPhen-2[11] and MutPred[12] ($\rho$>0.55, $P$<2.2×10$^{-16}$). Among missense variants in OXPHOS genes, 55% are consistently marked as being likely pathogenic by all three pathogenicity predictors (criteria: [**i**] CADD Phred score [version 1.3] >15; [**ii**] PolyPhen-2 of "possibly or probably damaging"; [**iii**] MutPred score >0.6), and 45% are annotated as being nonpathogenic by at least one of the predictors (**Supplementary Figure 7a**). The proportion of missense variants with a consensus prediction of "likely pathogenic" in OXPHOS genes is in line with the recommendation of MitoTIP that uses the medium pathogenicity to define "likely pathogenic" variants in mtDNA-encoded tRNA genes[10].

Therefore, we defined (**i**) missense variants that are consistently predicted to be pathogenic by CADD, PolyPhen-2, and MutPred, or nonsense variants (all with CADD Phred score>21), in OXPHOS genes, or (**ii**) variants predicted to be pathogenic (MitoTIP raw score>12.66) in tRNA genes, as predicted pathogenic (PP) variants in mtDNA. We found that this set of criteria would successfully classify 84% (=72/86) of confirmed mitochondrial disorder (MD)-causing mtDNA point mutations as PP variants according to the MITOMAP database[13] (last access in April 2021). Meanwhile, it would identify 92% (=789/862) of mtDNA variants with an annotation of "benign" in the ClinVar database[14] (last access in April 2021) as being nonpathogenic. To further increase sensitivity, we added all MD-causing point mutations[13] (n=86) to the list of PP variants.

Next, we investigated the frequencies of PP variants in the general population, including apparently healthy participants in HmtDB[15] (the Human Mitochondrial Database, 2020 update) and participants in the reference populations of gnomAD[16] (the Genome Aggregation Database, version 3.1.1), with a total of over 100,000 full-length mtDNA sequences. There was a high correlation of frequencies of mtDNA variants between HmtDB and gnomAD ($r$=0.99 and $\rho$=0.82). Because participants eligible for the reference populations of gnomAD were only filtered to exclude individuals diagnosed with a severe pediatric disorder and their first-degree relatives[16], we relied on the data from apparently healthy participants in HmtDB[15] for the study of frequencies of mtDNA variants in the general population described below.

We found that, of nonpathogenic variants in both OXPHOS genes and tRNA genes, about 9%-13% are polymorphic with a frequency >0.01% in the general population, while only less than 1% are polymorphic among PP variants (Fisher's exact test, $P$<2.2×10$^{-16}$; **Supplementary Figure 7b**). After focusing on variants that result from transition changes to reflect the predominant nucleotide substitution pattern in human mtDNA, we found that polymorphisms (frequency>0.01%) in the general population comprise up to 35% of nonpathogenic variants in

OXPHOS genes and tRNA genes but still account for less than 3% of variants predicted to be pathogenic (Fisher's exact test, $P<2.2\times10^{-16}$; **Supplementary Figure 7c**). These results suggest that PP variants in mtDNA are subject to strong purifying selection in humans. Accordingly, we further filtered PP variants by considering that the predicted, damaging effect of a variant allele should not be tolerated, at an appreciable frequency, among healthy participants.

To determine a proper threshold of population frequency for PP variants, we then examined the relationship between pathogenicity z-scores (inverse normalized CADD[9] and MitoTIP[10] raw scores) and population frequency among all variants in OXPHOS genes and tRNA genes. As expected, the pathogenicity z-score of a variant decreases as its frequency increases in the general population (**Supplementary Figure 7d**). However, a decrease in pathogenicity z-scores was found to be milder and nonsignificant after we raised the population frequency to be over 0.05% ($P\geq0.13$ between groups of variants with a population frequency >0.05%, **Supplementary Figure 7d**). We subsequently fit the relationship between pathogenicity z-scores and frequency of mtDNA variants in the general population to a nonlinear model by using spline regression with knots at frequencies of 0.01%, 0.02%, 0.05% 0.1%, 0.5% and 1%. As a result, we found that the fitted pathogenicity z-scores exhibit a steep decline from 0 to -1.1 at a population frequency of 0.05%, decrease smoothly thereafter, and plateau at -1.3 at a population frequency of 1% (**Supplementary Figure 7e**). Such a nonlinear change in pathogenicity z-scores was also revealed among variants that result from transition changes (**Supplementary Figure 7e**). The upper bound of the confidence interval of the fitted pathogenicity z-scores at a population frequency of 0.05% is at about one standard deviation below the median pathogenicity in mtDNA, illustrating that mtDNA variants above this threshold (population frequency>0.05%) are strongly shaped by purifying selection. As such, we required that PP heteroplasmies have a population frequency no more than 0.05% in the general population (including frequencies from both HmtDB and gnomAD). This additional criterion would increase specificity in determining "benign" variants in the ClinVar database[14] from 92% to 98% (=841/862). We further removed the 21 (=862-841) "benign" variants showing false positive predictions of pathogenicity[14] from the list of PP variants.

Moreover, mtDNA heteroplasmies with VAF between 10% and 95% were examined in the latest release of gnomAD (version 3.1.1)[16]. Their frequencies were summarized by age groups among 24,575 participants whose age information was available (data extracted from the "INFO" column in the VCF file gnomad.genomes.v3.1.sites.chrM.vcf)[16]. Among 13,613 participants aged under 60 years, there were 156 events of PP heteroplasmies recorded in gnomAD. In the SSC and BBC, we identified 127 PP heteroplasmies of VAF between 10% and 95%: 45 from 2,559 children with ASD or NDD, and 82 from 7,187 control participants and parents. Thus, the event rate of PP heteroplasmies was about 18 per one thousand participants affected by ASD or NDD (**Supplementary Figure 7f**), which was significantly higher than the corresponding rates among young and middle-aged participants in the reference populations of

gnomAD (Fisher's exact test, *P*=0.015; Exact binomial test, *P*=0.0067), as well as among unaffected participants in the current study (Fisher's exact test, *P*=0.025; Exact binomial test, *P*=0.0051).

Lastly, we assessed how robust our findings regarding the contribution of PP heteroplasmies to ASD are to changes in the criteria used to define PP heteroplasmies. First, we tested the influence of the choice of the maximum population frequency at 0.05% applied to PP variants in mtDNA. We found that the odds ratios of PP heteroplasmies for ASD were all significant and comparable when we used varying maximum population frequencies (from 0.01% to 1%) to filter PP heteroplasmies (OR≥1.49, *P*≤0.0036; **Supplementary Figure 7g**). PP heteroplasmies with a population frequency under 0.01% accounted for 2.4% of ASD risk, which was about 80% of that obtained using PP heteroplasmies with a population frequency under 1% (**Supplementary Figure 7h**). Therefore, the majority of ASD-associated PP heteroplasmies identified in the current study are of extremely rare frequencies in the general population. Second, we required PP heteroplasmies be further classified by at least one of the pathogenicity predictors as being probably pathogenic (criteria: [**i**] CADD[9] Phred score [version 1.3] >23, [**ii**] PolyPhen-2[11] of "probably damaging", [**iii**] MutPred[12] score >0.7, and [**iv**] MitoTIP[10] raw score >16.25). As a result, we found that using more rigid criteria to define probably pathogenic heteroplasmies led to more significant and greater effects of PP heteroplasmies on ASD risk (OR≥1.62, *P*≤0.0015; **Supplementary Figure 7g**). The contribution of probably pathogenic heteroplasmies to ASD constituted over 95% of the population-attributable risk proportion of all PP heteroplasmies for ASD (**Supplementary Figure 7h**), suggesting that the pathogenicity of most PP heteroplasmies identified in the current study are of relatively high confidence.

## Supplementary Figures

In all figures, the nominal *P* values are shown.

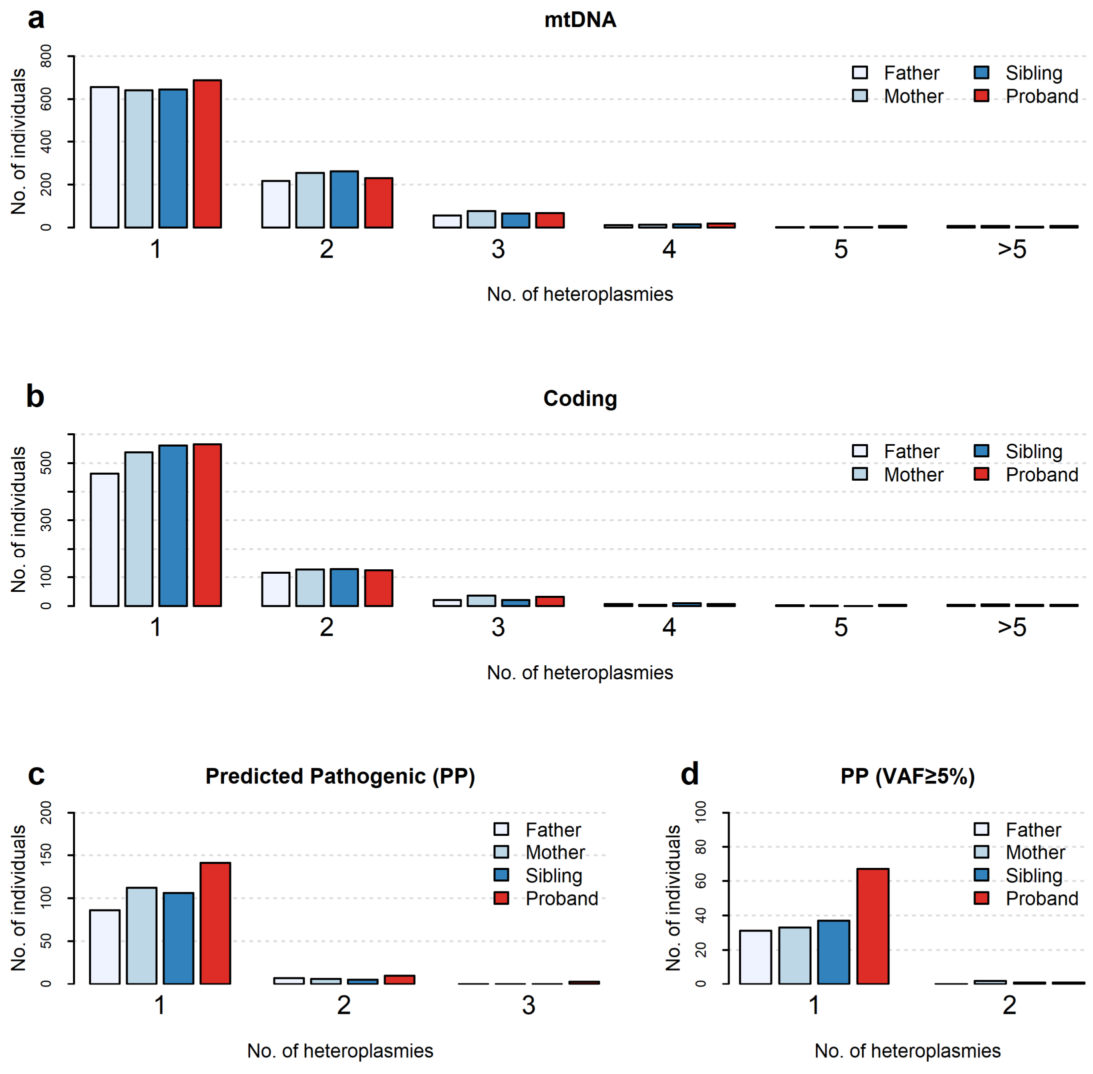


**Supplementary Figure 1. Distribution of the number of mtDNA heteroplasmies detected in each participant of the SSC.** Results were obtained from 1938 families (mother, father, sibling and proband with ASD) in the SSC as illustrated in Fig. 1a. (**a**) All mtDNA heteroplasmies; (**b**) heteroplasmies in the coding region; (**c**) predicted pathogenic heteroplasmies; (**d**) predicted pathogenic (PP) heteroplasmies with variant allele fraction (VAF) ≥5%.

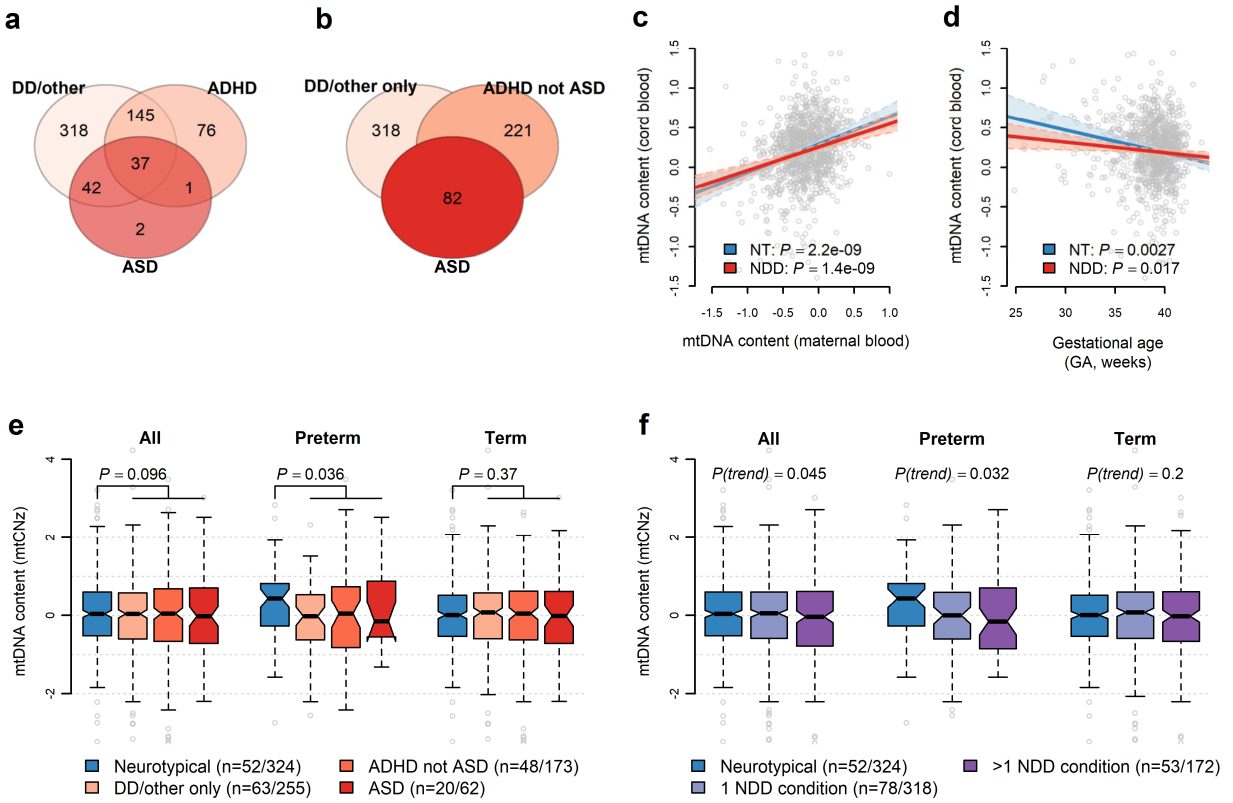


**Supplementary Figure 2. Associations of mtDNA content with NDD risks in the BBC. (a)** Venn diagram for children with autism spectrum disorder (ASD), attention-deficit/hyperactivity disorder (ADHD), and/or other developmental delay (DD) in the BBC. **(b)** Venn diagram for the three nonoverlapping neurodevelopmental disorder (NDD) groups in the BBC: ASD, ADHD not ASD, and DD/other only. (**c**) Scatter plots for mtDNA content in cord blood of children in relation to that in maternal peripheral blood collected at childbirth**.** (**d**) Scatter plots for mtDNA content in cord blood of children in relation to their gestational age**.** In **c** and **d**, the *P* value (two-sided) and the shaded area indicate the significance of the slope and the 95% confidence interval of the linear regression line (the solid line), respectively. (**e**, **f**) Box plots for the distribution of mtDNA content z-scores (mtCNz) in cord blood of children with neurotypical development and children in the three NDD groups (DD/other only, ADHD not ASD, and ASD as shown in **b**) (**e**) and in cord blood of children diagnosed with one NDD or more than one NDD (**f**). We depicted the distribution of mtCNz among children born preterm (gestational age at birth [GA]<37 weeks) in the middle subpanel of **e** and **f**, and the distribution of mtCNz among children born at term (GA≥37 weeks) in the right subpanel of **e** and **f**, to illustrate the interaction effect of low mtDNA content and prematurity on the risks of NDD. Sample sizes are shown in **e** and **f** as n=the number of children born preterm/the number of children born at term following the corresponding group label. Two-sided *P* values are from logistic regression in **e** and from linear regression in **f**. Box plots in **e** and **f** show the median as the center line, 95% confidence interval of the median as the

notch, the first (Q1) and third (Q3) quartiles as the boundaries of the box, the values of the largest and smallest data points within the range between Q1-1.5×interquartile range (IQR) and Q3+1.5×IQR as the boundaries of the whiskers, and the outliers beyond this range as the grey points.

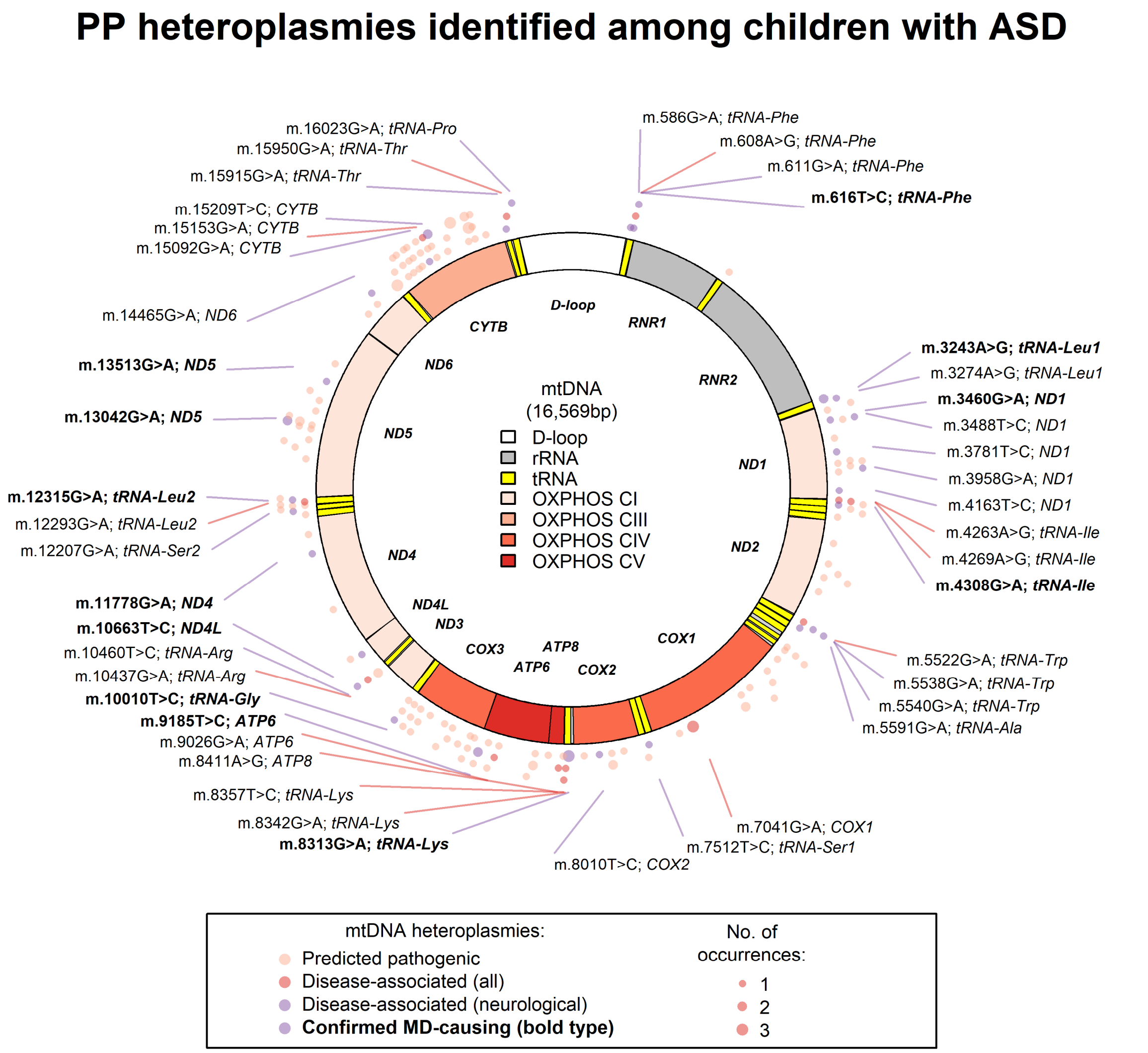


**Supplementary Figure 3. Predicted pathogenic heteroplasmies identified among children with ASD in the SSC and BBC.** The locations of predicted pathogenic (PP) heteroplasmies in mtDNA are shown with dots next to the circular, mitochondrial genome. The size of the dot indicates the number of occurrences (n=1, 2, 3) of this heteroplasmy detected among 2,020 children with ASD in the SSC and BBC. The distance of the dot to the mitochondrial genome

was added through jittering to minimize overlapping of adjacent dots. PP heteroplasmies that were reported to be associated with a disease (marked as “reported” or “confirmed”) in the MITOMAP database or were marked as “likely pathogenic” or “pathogenic” in the ClinVar database are depicted with red dots, or purple dots if the associated disease has a neurological manifestation. Other PP heteroplasmies are depicted with pink dots. The variant allele and affected gene of all disease-associated PP heteroplasmies are indicated. PP heteroplasmies confirmed to cause mitochondrial disorders (MD) in the MITOMAP database are shown in bold type. Diseases with a neurological manifestation were determined through searching the associated disease annotation in MITOMAP and ClinVar by keywords: “neuropathy”, “encephalopathy”, “encephalomyopathy”, “encephalocardiomyopathy”, “autism”, “cognitive decline”, “developmental delay”, “intellectual disability”, “mental deterioration”, “neuropsychiatric”, “psychosis”, “dementia”, “epilepsy”, “seizure”, “stroke”, “cerebellar ataxia”, “striatal necrosis”, “blindness”, “deafness”, “Leigh”, “Alzheimer”, “Parkinson”, “Prader-Willi”, “Angelman syndrome”, “Kearns Sayre Syndrome”, “Pearson syndrome”, and “Rett Syndrome”; and the abbreviations of the diseases listed on the MITOMAP website (https://www.mitomap.org/MITOMAP/DiseaseList): “AD”, “ADPD”, “AMDF”, “DEAF”, “DEMCHO”, “ESOC”, “FBSN”, “KSS”, “LDYT”, “LHON”, “MEc”, “MELAS”, “MEm”, “MEPR”, “MERME”, “MERRF”, “MILS”, “MNGIE”, “NAION”, “NARP”, “PEG”, “PEM”, “PME”, “POAG”, “RTT”, and “SNHL”.

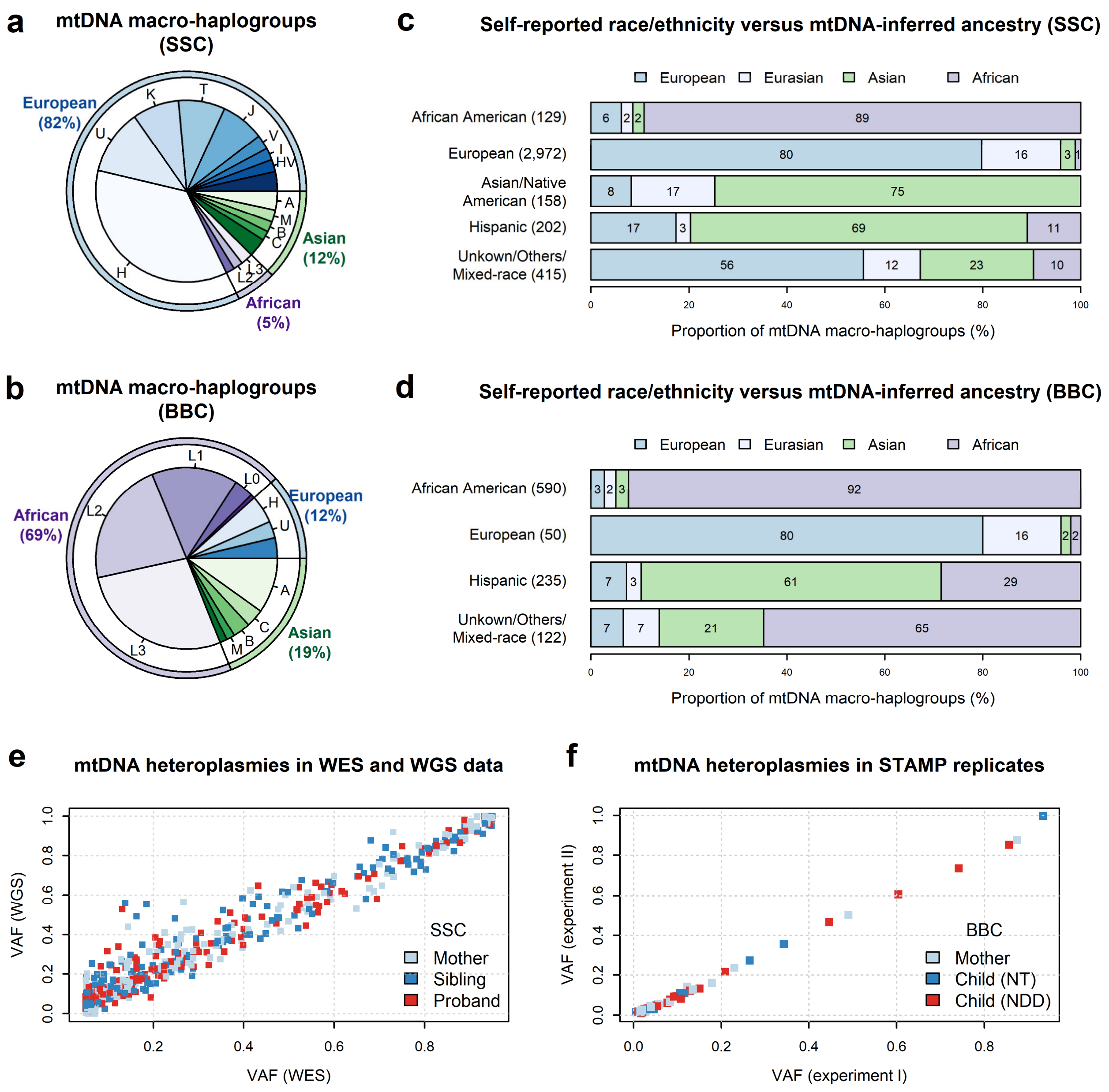


**Supplementary Figure 4. Comparison of mtDNA-inferred ancestry with self-reported race as well as comparison of heteroplasmies between experimental replicates.** (**a**, **b**) Pie plots for the proportions of mtDNA macro-haplogroups among mothers and fathers of the SSC (**a**) and children of the BBC (**b**). The three slices without an alphabetical label represent all other macro-haplogroups in the respective racial group. (**c**, **d**) The bar plots illustrate the ancestry proportion inferred from mtDNA macro-haplogroups for mothers and fathers in the SSC (**c**) and children in the BBC (**d**), stratified by self-reported race or ethnicity. The value of the ancestry proportion is shown within the respective bar, which was rounded to the nearest integer. The total number of participants in each racial or ethnic group is indicated in parentheses following the ancestry label. mtDNA haplogroup information was determined using haplogrep2 (v2.1.1)[17] based on the

major mtDNA sequence (with variant allele fraction [VAF] >50%) of each sample. mtDNA macro-haplogroups were categorized into European, Asian, or African types according to the phylogenetic tree of human mtDNA. The four mtDNA macro-haplogroups (i.e., "U", "N", "R", and "X") were considered as Eurasian type. **(e)** Scatter plots for VAF of mtDNA heteroplasmies identified using the whole-exome sequencing (WES) data and the whole-genome sequencing (WGS) data of the SSC. mtDNA heteroplasmies that were used for analysis in our previous study using the WES data of the SSC are shown[8]. (**f**) Scatter plots for VAF of mtDNA heteroplasmies in 102 pairs of technical replicates detected using STAMP in the BBC. In both **e** and **f**, the correlations in VAF were estimated to be Pearson's $r \geq 0.98$ and Spearman's $\rho \geq 0.94$ ($P<2.2\times10^{-16}$). NT: neurotypical; NDD: neurodevelopmental disorder.

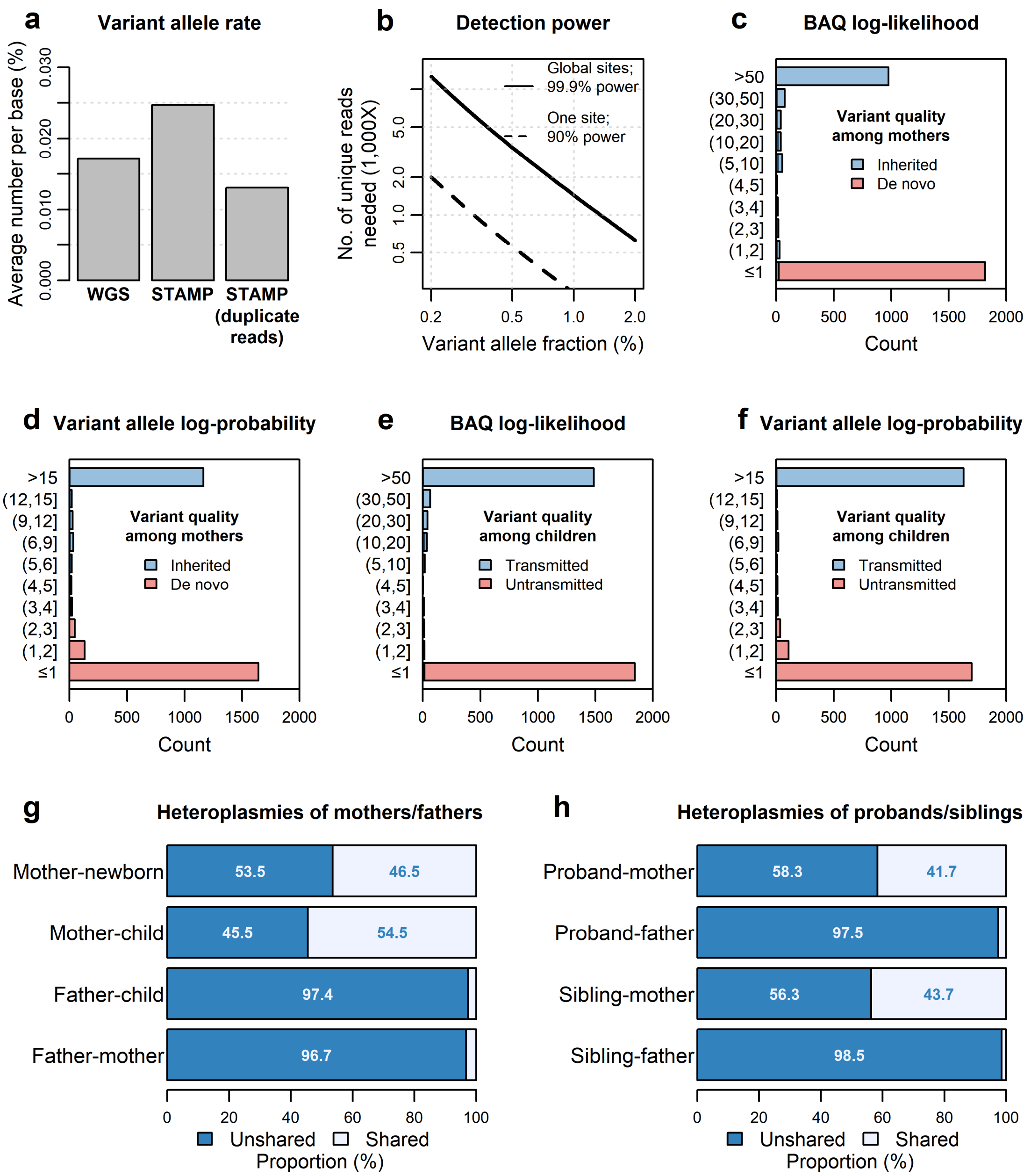


**Supplementary Figure 5. Detection power and quality of mtDNA heteroplasmies as well as proportions of mtDNA heteroplasmies shared between family members.** (**a**) Bar plots for the average proportions of variant alleles in the unique reads retained for heteroplasmy detection in the whole-genome sequencing (WGS) data from the SSC and the mtDNA-targeted sequencing (STAMP) data from the BBC. The right bar represents the result computed using consensus reads constructed with multiple paired-end reads of the same mtDNA fragment in STAMP. (**b**) Line plots for the number of unique reads needed to discriminate real variants from errors (error rate, ε=0.02% per base) at all mtDNA sites (99.9% power at alpha=0.05/16569; solid line) as well as at one mtDNA site (90% power at alpha=0.05; dashed line). Results were obtained from

a one-tailed power calculation for one sample proportion. Values on both axes are on a log scale. (**c-f**) Bar plots for the distribution of quality scores of the variant alleles of children's inherited heteroplasmies and *de novo* heteroplasmies in mothers (**c**, **d**) as well as mothers' transmitted heteroplasmies and untransmitted heteroplasmies in children (**e**, **f**). BAQ stands for base alignment quality. The quality score in **d** and **f** represents the minus log-probability from an exact *Poisson* test for the observed number of variant alleles against the expected number of errors (error rate, $\varepsilon$=0.02% per base). For both quality metrics, a score of 5 represents a false positive rate of $10^{-5}$. (**g**, **h**) Bar plots for the proportion of shared heteroplasmies in the samples of family members in the SSC as well as in the maternal and newborn samples in the BBC. mtDNA heteroplasmies present (with variant allele fraction ≥0.2%) in all samples from a family in the SSC or at mtDNA sites with the major allele different between the mother and the father were excluded.

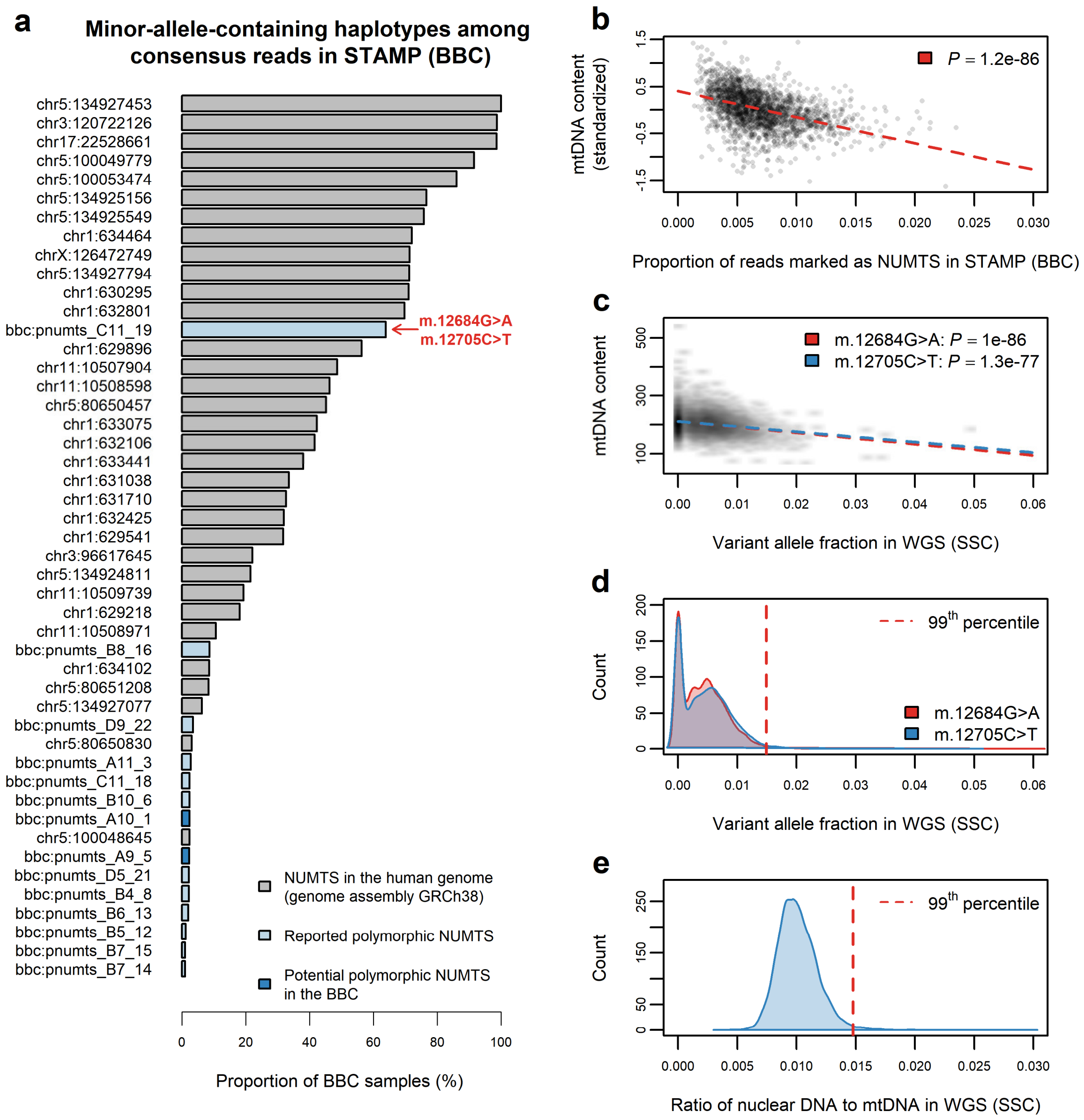


**Supplementary Figure 6. Characteristics of variants associated with nuclear mitochondrial segments.** (**a**) Bar plots for the frequencies of the 47 haplotypes that contain minor alleles among consensus reads identified in BBC samples. The red arrow marks the most abundant haplotype of nuclear mitochondrial segments (NUMTS) that have not been annotated in the reference nuclear genome, which contains two variant alleles at m.12684G>A and m.12705C>T. (**b**) Scatter plots for the proportion of consensus reads marked as NUMTS in relation to mtDNA content obtained from the STAMP data from the BBC. (**c**) Scatter plots with smoothed color density representation for the allele fraction of m.12684G>A and m.12705C>T in relation to mtDNA content estimated using the whole-genome sequencing (WGS) data of the SSC. In **b** and **c**, the dashed line illustrates the fitted values from linear regression and the *P* value (two-sided) indicates the significance of the slope. (**d**) Density plots for the fraction of m.12684G>A and m.12705C>T in the WGS data of the SSC. (**e**) Density plot for the ratio of nuclear DNA to

mtDNA in the WGS data of the SSC, which is equal to two divided by the value of mtDNA content. The 99th percentile of the distribution is indicated by the red vertical dashed line.

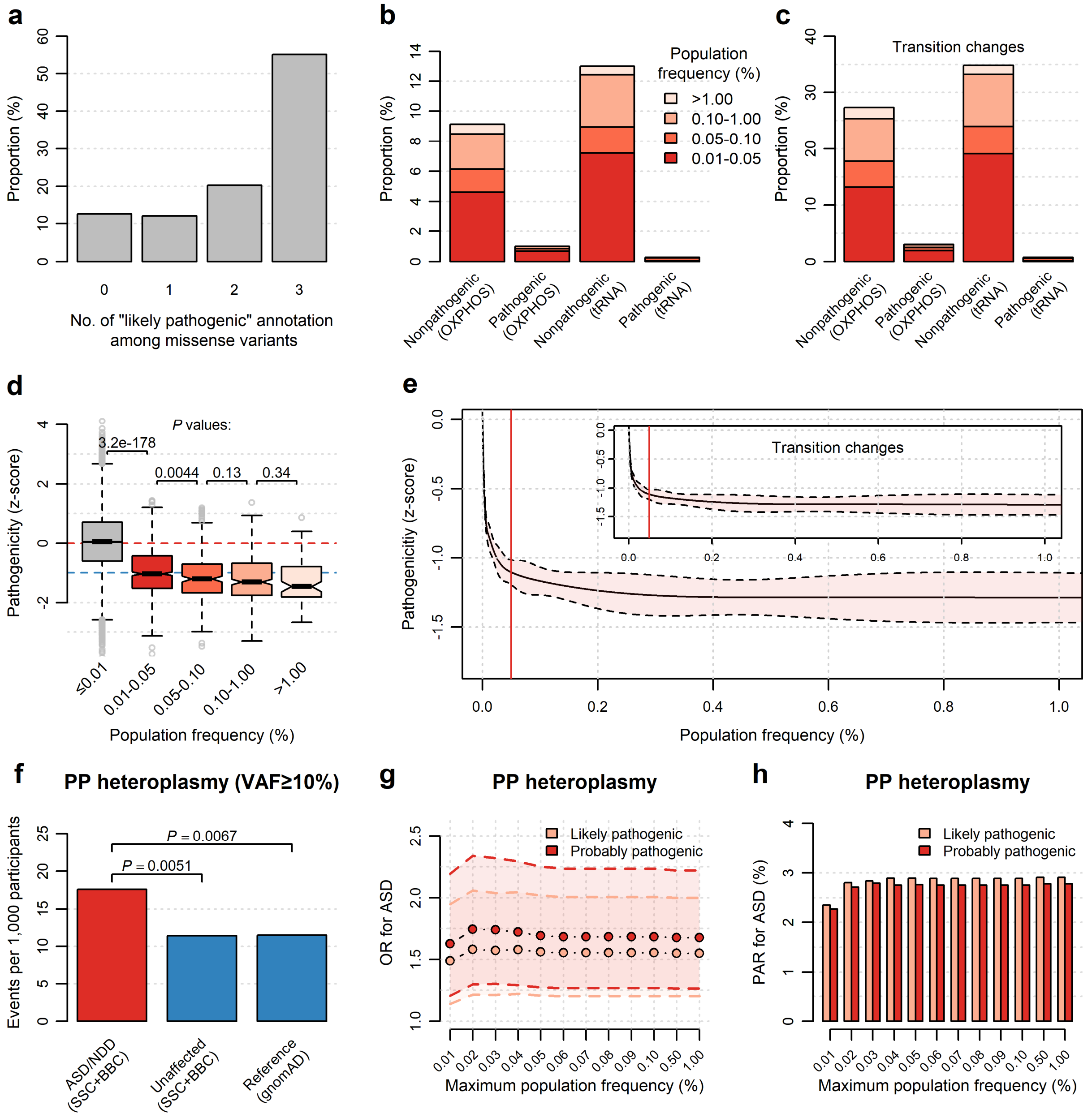


**Supplementary Figure 7. mtDNA variant pathogenicity and frequency in the general population and sensitivity analysis of the definition of predicted pathogenic heteroplasmy.** **(a)** Histogram for the number of "likely pathogenic" annotation from CADD, PolyPhen-2 and MutPred among missense variants in oxidative phosphorylation (OXPHOS) genes. (**b, c**) Bar plots for the respective proportions of nonsynonymous variants in OXPHOS genes and variants

in tRNA genes that are found to be polymorphic with a frequency >0.01% in the general population. We also show the results computed using mtDNA variants that result from transition changes (**c**) to reflect the predominant nucleotide substitution pattern in human mtDNA. (**d**) Box plots for the distribution of pathogenicity z-scores of mtDNA variants stratified by the frequency groups. Box plots show the median as the center line, 95% confidence interval of the median as the notch, the first (Q1) and third (Q3) quartiles as the boundaries of the box, the values of the largest and smallest data points within the range between Q1-1.5×interquartile range (IQR) and Q3+1.5×IQR as the boundaries of the whiskers, and the outliers beyond this range as the grey points. Two-sided *P* values were obtained from linear regression adjusted for the variant's location in mtDNA (OXPHOS/RNA genes). (**e**) Line plots for the fitted pathogenicity z-scores from spline regression. The solid line represents the fitted value, and the dashed lines represent the 95% confidence interval. The red vertical line indicates a population frequency of 0.05%. (**f**) Bar plots for the event rate of predicted pathogenic (PP) heteroplasmies with variant allele fraction (VAF) between 10% and 95% among 2,559 children with autism spectrum disorder (ASD) or neurodevelopmental disorder (NDD) and 7,187 control participants and parents in the current study, as well as among 13,613 young and middle-aged participants in the reference populations of gnomAD. Two-sided *P* values were obtained from an Exact binomial test. (**g**) Scatter plots for the odds ratios (ORs) for ASD of PP heteroplasmies defined using varying criteria. The dashed lines represent the 95% confidence interval of the estimated ORs. (**h**) Bar plots for the population-attributable risk proportions (PARs) for ASD of PP heteroplasmies defined using varying criteria.

# Supplementary Tables

In all tables, the E notation (mE-n) indicates a value of $m \times 10^{-n}$; the nominal P values are reported.

## Supplementary Table 1. Association of mtDNA heteroplasmy incidence with ASD in the SSC.

| Group* | Proband versus Father | | Proband versus Mother | | Proband versus Sibling | |
|---|---|---|---|---|---|---|
| | **OR [95% CI]** | ***P*** | **OR [95% CI]** | ***P*** | **OR [95% CI]** | ***P*** |
| All | 1.05 [0.99-1.13] | 0.11 | 0.99 [0.91-1.08] | 0.83 | 1.04 [0.95-1.13] | 0.39 |
| Synonymous | 1.09 [0.95-1.25] | 0.20 | 0.87 [0.71-1.07] | 0.19 | 1.01 [0.84-1.22] | 0.92 |
| Nonsynonymous | 1.15 [1.00-1.32] | 0.057 | 1.01 [0.85-1.20] | 0.93 | 1.05 [0.89-1.24] | 0.53 |
| rRNA | 0.77 [0.59-1.02] | 0.065 | 0.83 [0.60-1.14] | 0.25 | 0.88 [0.65-1.19] | 0.40 |
| tRNA | 1.99 [1.47-2.69] | 8.2E-06 | 2.25 [1.59-3.20] | 5.9E-06 | 1.40 [1.04-1.87] | 0.024 |

The odds ratios (ORs) of the number of mtDNA heteroplasmies for ASD and the *P* values (two-sided) were obtained from conditional logistic regression by comparing the heteroplasmy incidence in probands to that in fathers, mothers, and siblings, respectively. mtDNA heteroplasmies with variant allele fraction (VAF)>95% detected at heteroplasmic mtDNA sites in participants from the same family were also used to count heteroplasmy incidence for conditional logistic regression analysis using matched samples. For comparisons between probands and fathers, we only considered secondary heteroplasmies detected at heteroplasmic mtDNA sites in mother-proband-sibling trios.

*Analyses were performed using all heteroplasmies and heteroplasmies in genes encoding oxidative phosphorylation protein complexes (at synonymous sites and nonsynonymous sites, respectively), genes encoding rRNAs, and genes encoding tRNAs, respectively.

**Supplementary Table 2. Correlations of PP heteroplasmies with nuclear risk factors of ASD in the SSC.**

| Nuclear risk factor* | Description | Variable | Kendall's τ | *P*† |
|---|---|---|---|---|
| ASD-associated common variants (n=1,872) | The polygenic risk score (PRS) for ASD was obtained from Supplementary Table 21 of Wilfert et al. It was computed using all SNPs associated with ASD at $P$<0.01 in the genome-wide association study summary statistics from Grove et al. SNPs were thinned based on linkage disequilibrium and weighted with the SNPs' OR for computation of PRS. The polygenic transmission disequilibrium test (pTDT) scores were computed as ($PRS_{child}$-($PRS_{mother}$+$PRS_{father}$)/2) divided by the standard deviation of ($PRS_{mother}$+$PRS_{father}$)/2. | pTDT score in the $95^{th}$ percentile | -0.03 | 0.18 (0.21) |
| | | pTDT score in the $75^{th}$ percentile | -0.02 | 0.35 (0.34) |
| | | Continuous pTDT score | -0.02 | 0.30 (0.29) |
| *De novo* coding mutations (n=1,846) | Information on *de novo* coding mutations and their functional annotation were extracted from Supplementary Table 1 of Iossifov et al. *De novo* mutations (DNM) were detected using the whole-exome sequencing data of the SSC. Likely gene-disrupting (LGD) mutations included nonsense, frameshift and splice site mutations. | No. of LGD DNM in recurrent genes among probands | 0.03 | 0.15 (0.33) |
| | | No. of LGD DNM | 0.01 | 0.61 (0.86) |
| | | No. of LGD and missense DNM | -0.01 | 0.54 (0.38) |
| *De novo* noncoding mutations (n=1,786) | Information on *de novo* noncoding mutations and their functional annotation (including predicted disease impact scores [DIS]) were extracted from Supplementary Table 1 of Zhou et al. We considered 69,328 variants near all genes for DNA and 4,871 variants near alternatively spliced exons for RNA (Figure 1 of Zhou et al.) in the analysis for all genes. Predicted ASD risk genes (TADA FDR≤0.1) were obtained from Table S2 of Satterstrom et al. | No. of damaging DNM (DIS >0) near ASD risk genes | -0.04 | 0.10 (0.11) |
| | | No. of damaging DNM (DIS >0) near all genes | 0.01 | 0.59 (0.69) |
| *De novo* structural variants (n=1,737) | Information on *de novo* structural variants in the coding region of the nuclear genome were extracted from Supplementary Table 13 of Krumm et al. Structural variants were identified using the whole-exome sequencing data of the SSC and were validated using the SNP microarray data. | No. of *de novo* structural variants | -0.03 | 0.29 (0.19) |

*N indicates the number of overlapping SSC families between the previous study and the current study.

†*P* values (two-sided) for the Kendall's τ test for the correlation of the number of PP heteroplasmies with the number/score of the nuclear risk factor, as well as for the Mann-Whitney test (shown in parentheses) for the difference in the nuclear risk factor between probands with and without PP heteroplasmies.

pTDT: polygenic transmission disequilibrium test.

LGD: likely gene-disrupting.

DNM: *de novo* mutation.

DIS: predicted disease impact score.

**Supplementary Table 3. Effects of PP heteroplasmies on ASD stratified by nuclear risk factors identified in children with ASD in the SSC.**

| Nuclear risk factor | Group based on the nuclear risk factors in probands | N* | Effect in subpopulations | | Nuclear-adjusted effect in all families | |
|---|---|---|---|---|---|---|
| | | | OR [95% CI] | *P* | OR [95% CI] | *P* |
| ASD-associated common variants | (a) pTDT score in the 95th percentile | 94 | 1.00 [0.20-4.95] | 1.00 | 1.53 [1.19-1.96] | 0.00090 |
| | pTDT score in the 75th percentile | 468 | 1.44 [0.86-2.42] | 0.16 | | |
| | Other† | 1470 | 1.53 [1.15-2.03] | 0.0033 | | |
| *De novo* coding mutations | LGD DNM in recurrent genes among probands | 54 | 1.20 [0.36-3.96] | 0.76 | 1.54 [1.19-1.98] | 0.00098 |
| | (b) LGD DNM | 282 | 1.42 [0.75-2.66] | 0.28 | | |
| | LGD and missense DNM | 1013 | 1.40 [1.01-1.94] | 0.045 | | |
| | Other† | 925 | 1.67 [1.14-2.43] | 0.0082 | | |
| *De novo* noncoding mutations | (c) Excessive damaging DNM (DIS>0) near ASD risk genes‡ | 168 | 1.00 [0.35-2.85] | 1.00 | 1.52 [1.19-1.96] | 0.00098 |
| | Excessive damaging DNM (DIS>0) near all genes‡ | 896 | 1.44 [0.98-2.11] | 0.061 | | |
| | Other† | 1042 | 1.56 [1.12-2.16] | 0.0076 | | |
| *De novo* structural variants | (d) *De novo* structural variants | 127 | 1.20 [0.37-3.93] | 0.76 | 1.53 [1.19-1.96] | 0.00089 |
| | Other† | 1811 | 1.52 [1.18-1.96] | 0.0012 | | |
| All risk factors tested | Either of (a), (b), (c) and (d) | 588 | 1.13 [0.70-1.83] | 0.62 | 1.59 [1.22-2.06] | 0.00052 |
| | Other† | 1350 | 1.67 [1.25-2.23] | 0.00060 | | |

Detailed explanations on the four types of nuclear risk factors for ASD and the related variables used in each test are provided in Supplementary Table 2. The nuclear-adjusted effect in all families represents the effect estimated using conditional logistic regression with adjustment for the variable(s) representing either one of the four types of nuclear risk factors or all four types of nuclear risk factors tested. *P* values (two-sided) are from conditional logistic regression.

*Number of families.

†All other families from the SSC in the current study.

‡More *de novo* mutations were detected in the proband as compared to the sibling.

pTDT: polygenic transmission disequilibrium test.

LGD: likely gene-disrupting.

DNM: *de novo* mutation.

DIS: predicted disease impact score.

## Supplementary Table 4. Analysis of the effects of purifying selection on inherited heteroplasmies and *de novo* heteroplasmies in mtDNA.

| Study | Group | OXPHOS* | | tRNA* | | All* | |
|---|---|---|---|---|---|---|---|
| | | Patho-genicity† | *P* (selection)‡ | Patho-genicity† | *P* (selection)‡ | Patho-genicity† | *P* (selection)‡ |
| SSC | Both inherited | -1.00 (-0.83) | 1.9E-11 (<1.0E-05) | -1.06 (-1.07) | 3.6E-05 (<1.0E-05) | -1.03 (-0.88) | 3.3E-15 (<1.0E-05) |
| | Sibling inherited only | -1.06 (-1.05) | 1.2E-10 (<1.0E-05) | -0.62 (-0.82) | 0.074 (0.012) | -1.05 (-1.02) | 2.9E-11 (<1.0E-05) |
| | Both/sibling inherited | -1.04 (-0.92) | 1.3E-20 (<1.0E-05) | -1.01 (-0.99) | 1.0E-05 (<1.0E-05) | -1.04 (-0.93) | 4.0E-25 (<1.0E-05) |
| | Proband inherited only | -0.52 (-0.49) | 0.00036 (0.00085) | -0.95 (-0.83) | 0.24 (0.033) | -0.52 (-0.52) | 0.00015 (0.00027) |
| | Sibling *de novo* | -0.64 (-0.57) | 3.4E-15 (<1.0E-05) | -0.51 (-0.29) | 0.012 (0.080) | -0.61 (-0.50) | 3.0E-16 (<1.0E-05) |
| | Proband *de novo* | -0.57 (-0.56) | 2.4E-15 (<1.0E-05) | -0.12 (-0.19) | 0.076 (0.27) | -0.48 (-0.45) | 5.5E-14 (<1.0E-05) |
| BBC§ | Child (NT) inherited | -1.18 (-0.85) | 2.4E-05 (<1.0E-05) | -0.85 (-0.87) | 0.030 (0.062) | -1.11 (-0.85) | 4.0E-06 (<1.0E-05) |
| | Child (NDD) inherited | -0.97 (-0.96) | 1.3E-10 (<1.0E-05) | -1.06 (-0.81) | 0.0043 (0.0035) | -0.99 (-0.93) | 1.4E-12 (<1.0E-05) |
| | Child (ASD) inherited | -0.90 (-1.07) | 0.00017 (0.00070) | -0.57 (-0.57) | NA (0.30) | -0.75 (-1.03) | 0.00010 (0.00077) |
| | Child (NT) *de novo* | -0.45 (-0.46) | 0.0078 (0.011) | -0.07 (-0.25) | 0.28 (0.33) | -0.32 (-0.41) | 0.0038 (0.012) |
| | Child (NDD) *de novo* | -0.73 (-0.61) | 6E-06 (<1.0E-05) | -0.25 (-0.35) | 0.11 (0.14) | -0.56 (-0.55) | 1.6E-06 (<1.0E-05) |
| | Child (ASD) *de novo* | -0.03 (-0.38) | 0.25 (0.12) | -0.19 (-0.14) | 0.85 (0.48) | -0.03 (-0.34) | 0.25 (0.14) |

*Analyses were performed using heteroplasmies at nonsynonymous sites in genes encoding oxidative phosphorylation protein complexes (OXPHOS) and heteroplasmies in genes encoding tRNAs (tRNA), separately, and OXPHOS heteroplasmies and tRNA heteroplasmies combined (All).

†Data are median (mean) of pathogenicity z-scores.

‡*P* values are from a one-sample t-test with the null hypothesis assuming an average pathogenicity z-score of zero; *P* values shown in parentheses are from a bootstrapping-based test, representing the proportion of the mean pathogenicity z-scores, from $10^5$ random resamples of nucleotide changes in mtDNA, that are lower than the observed value.

§Results are shown for children with a diagnosis of any neurodevelopmental disorder (NDD), children with autism spectrum disorder (ASD) and children with neurotypical development (NT), respectively.

NA: not applicable, as only one heteroplasmy was identified.

**Supplementary Table 5. Influence of maternal age at childbirth on the number of inherited heteroplasmies and *de novo* heteroplasmies in the SSC.**

| Model | Group | mtDNA (16569)* | | OXPHOS & tRNA (10286)* | |
|---|---|---|---|---|---|
| | | Beta (SE) | *P* | Beta (SE) | *P* |
| No. of inherited heteroplasmies ~ maternal age | Sibling | 0.0073 (0.0047) | 0.12 | 0.0014 (0.0016) | 0.37 |
| | Proband | 0.0025 (0.0043) | 0.57 | 0.0017 (0.0016) | 0.27 |
| | All† | 0.0046 (0.0032) | 0.14 | 0.0016 (0.0011) | 0.16 |
| No. of *de novo* heteroplasmies ~ maternal age | Sibling | 0.0099 (0.0029) | 0.00073 | 0.0048 (0.0019) | 0.013 |
| | Proband | 0.011 (0.0030) | 0.00015 | 0.0050 (0.0020) | 0.013 |
| | All† | 0.011 (0.0021) | 3.9E-07 | 0.0049 (0.0014) | 0.00044 |
| No. of *de novo* heteroplasmies ~ maternal age + paternal age | Sibling | 0.0089 (0.0042) | 0.035 | 0.0051 (0.0028) | 0.066 |
| | Proband | 0.0081 (0.0043) | 0.058 | 0.0042 (0.0029) | 0.14 |
| | All† | 0.0085 (0.0030) | 0.0046 | 0.0047 (0.0020) | 0.019 |

The effects (linear regression coefficient, beta) and the two-sided *P* values are shown for maternal age at childbirth in all models.

*Analyses were performed using heteroplasmies at all 16,569 mtDNA sites (mtDNA) and heteroplasmies at nonsynonymous sites in oxidative phosphorylation genes or at all sites in tRNA genes (OXPHOS & tRNA); the number of sites shown in parentheses were averaged over all possible nucleotide changes.

†Results from fixed-effect meta-analysis.

**Supplementary Table 6. Influence of maternal age at childbirth on pathogenicity of *de novo* heteroplasmies in the SSC.**

| Group | <25 years * | | 25-30 years * | | 30-35 years * | | ≥35 years * | | *P* trend † |
|---|---|---|---|---|---|---|---|---|---|
| | Patho-genicity | *P* (selection) | Patho-genicity | *P* (selection) | Patho-genicity | *P* (selection) | Patho-genicity | *P* (selection) | |
| Sibling | -1.02 (-0.85) | 6.8E-05 (0.00027) | -0.64 (-0.54) | 0.00024 (0.00031) | -0.48 (-0.40) | 2.5E-05 (0.00051) | -0.44 (-0.34) | 0.013 (0.044) | 0.040 (0.086) |
| Proband | -0.86 (-0.86) | 0.00018 (2.0E-05) | -0.60 (-0.51) | 0.00014 (0.00029) | -0.39 (-0.35) | 0.00019 (0.0021) | -0.42 (-0.41) | 5.9E-05 (0.0017) | 0.078 (0.14) |
| All | -0.91 (-0.86) | 4.9E-08 (<1E-05) | -0.62 (-0.52) | 1.1E-07 (<1E-05) | -0.48 (-0.38) | 2.1E-08 (3.0E-05) | -0.43 (-0.38) | 2.4E-06 (0.00044) | 0.0069 (0.023) |

*Data are median (mean) of pathogenicity z-scores of *de novo* heteroplasmies among children in the four groups based on their mothers' age at birth; *P* values (*P* for selection) are from a one-sample t-test with the null hypothesis assuming an average pathogenicity z-score of zero and the *P* values shown in parentheses are from a bootstrapping-based test, representing the proportion of the mean pathogenicity z-scores, from $10^5$ random resamples of nucleotide changes in mtDNA, that are lower than the observed value.

†*P* for trend (two-sided) was obtained from linear regression of pathogenicity z-scores on the four maternal-age groups (as integers from 1 to 4), adjusted for the location in mtDNA (OXPHOS or tRNA), the variant allele fraction of *de novo* heteroplasmies, child sex and disease status (in the analysis with combined samples [All]). The *P* values from analyses with additional adjustment for paternal age at childbirth are indicated in parentheses.

**Supplementary Table 7. Association of mtDNA content with ASD stratified by child sex or parental race in the SSC.**

| Stratification | | N* | OR [95% CI] | *P* |
|---|---|---|---|---|
| By proband sex: | Female | 256 | 0.64 [0.52-0.80] | 9.6E-05 |
| | Male | 1682 | 0.68 [0.63-0.74] | 5.7E-20 |
| By sibling sex: | Female | 1023 | 0.75 [0.67-0.83] | 4.9E-08 |
| | Male | 915 | 0.60 [0.53-0.67] | 1.9E-17 |
| By maternal race: | European | 1450 | 0.69 [0.63-0.76] | 6.7E-16 |
| | Others | 488 | 0.63 [0.54-0.73] | 5.5E-09 |
| By paternal race: | European | 1522 | 0.68 [0.62-0.74] | 8.5E-18 |
| | Others | 416 | 0.65 [0.55-0.77] | 5.7E-07 |

The odds ratios (ORs) of per standard deviation increase in the mtDNA content z-score (mtCNz) for ASD and the two-sided *P* values were obtained using conditional logistic regression among families in the SSC stratified by child (proband or sibling) sex or parental (maternal or paternal) race.

*The number of families used in each test.

**Supplementary Table 8. Interaction effects of PP heteroplasmies and mtDNA content on ASD risk in the SSC.**

| Group | I (Reference) | II | III | IV | *P* (interaction)* |
|---|---|---|---|---|---|
| mtCNz | medium/high | medium/high | low | low | |
| PP heteroplasmy | without | with | without | with | |
| All (model 1) | 1 | 1.21 [0.86-1.70] | 1.94 [1.66-2.26] | 3.67 [2.32-5.80] | 0.12 |
| VAF≥5% (model 1) | 1 | 1.74 [1.01-2.98] | 1.98 [1.70-2.30] | 7.58 [2.88-19.93] | 0.15 |
| All (model 2)† | 1 | 1.22 [0.85-1.73] | 1.96 [1.67-2.31] | 3.98 [2.49-6.35] | 0.083 |
| VAF≥5% (model 2)† | 1 | 1.56 [0.90-2.73] | 2.00 [1.71-2.34] | 9.60 [3.59-25.67] | 0.044 |

The estimated odds ratio and its 95% confidence interval are shown in relation to group I (the reference group) representing those having medium/high mtCNz (in the middle-tertile or high-tertile group of mtDNA content z-scores) and no predicted pathogenic (PP) heteroplasmies.

**P*(interaction) indicates the *P* value (two-sided) of the interaction effect between low mtCNz (in the low-tertile group of mtCNz) and carrying PP heteroplasmies estimated using conditional logistic regression.

†Maternal age at childbirth, paternal age at childbirth and age of children at the time of sample collection were further adjusted in model 2 in relation to model 1 using conditional logistic regression.

**Supplementary Table 9. Associations of PP heteroplasmies and mtDNA content with neurological traits among children with ASD in the SSC.**

| Trait* | N† | PP heteroplasmy‡ | | | mtCNz‡ | | |
|---|---|---|---|---|---|---|---|
| | | OR [95% CI] | *P* (model 1) | *P* (model 2) | OR† [95% CI] | *P* (model 1) | *P* (model 2) |
| NV-IQ < 70 | 1938 | 1.70 [1.19-2.43] | 0.0036 | 0.0038 | 1.01 [0.91-1.11] | 0.92 | 0.58 |
| V-IQ < 70 | 1938 | 1.41 [1.00-1.99] | 0.050 | 0.030 | 1.02 [0.93-1.12] | 0.66 | 0.94 |
| VABS in the bottom 25% | 1938 | 1.86 [1.29-2.66] | 0.00079 | 0.00073 | 1.03 [0.93-1.14] | 0.59 | 0.94 |
| (-) ADOS-CS in the bottom 25% | 1938 | 1.66 [1.18-2.34] | 0.0039 | 0.0022 | 1.05 [0.95-1.15] | 0.34 | 0.44 |
| (-) ADOS-RB in the bottom 25% | 1938 | 1.19 [0.85-1.68] | 0.31 | 0.26 | 1.04 [0.95-1.13] | 0.45 | 0.54 |
| Improvements in: | | | | | | | |
| Cognition | 1502 | 1.27 [0.49-3.26] | 0.63 | 0.56 | 1.01 [0.76-1.34] | 0.96 | 0.94 |
| Social interaction | 1512 | 1.29 [0.65-2.56] | 0.47 | 0.52 | 1.19 [0.98-1.44] | 0.084 | 0.13 |
| Communication | 1510 | 1.97 [1.13-3.43] | 0.016 | 0.031 | 1.12 [0.93-1.34] | 0.22 | 0.39 |
| Repetitive behavior | 1512 | 3.52 [2.07-5.97] | 3.3E-06 | 1.0E-05 | 1.11 [0.90-1.35] | 0.33 | 0.51 |
| Temper | 1516 | 2.13 [1.30-3.52] | 0.0029 | 0.0025 | 1.11 [0.94-1.31] | 0.20 | 0.25 |

*The associations of carrying PP heteroplasmies and the mtDNA content z-score (mtCNz) with ASD-related neurodevelopmental traits, as well as improvements in ASD-related symptoms according to the medical records were assessed using logistic regression. Two-sided *P* values are shown. NV-IQ: non-verbal IQ; V-IQ: verbal IQ; VABS: the individual's Adaptive Behavior Composite from Vineland Adaptive Behavior Scales (2nd edition); ADOS-CS: the communication and social total from the Autism Diagnostic Observation Schedule (ADOS); ADOS-RB: the restricted and repetitive behavior total (module 1-3) or the stereotyped behaviors and restricted interests total (module 4) from the ADOS. Because the ADOS records scores of abnormalities, the additive inverse of the original value was used.

†The number of probands with related data available for the association analysis of each trait.

‡In addition to adjustment for age, sex, and mtDNA-inferred ancestry in model 1, maternal age at childbirth, paternal age at childbirth, and the total number of hours and weeks of biomedical therapies, intensive therapies, occupational therapies, and speech therapies that each participant had received before the time of sample collection were further adjusted in model 2. The odds ratios (ORs) of carrying predicted pathogenic (PP) heteroplasmies and mtCNz (mtDNA content z-score) were assessed jointly in model 1 and model 2. The ORs provided are from model 1.

**Supplementary Table 10. Associations of mtDNA content and gestational age with NDD risks in the BBC.**

| Variable | GA, weeks | | mtCNz | | mtCNz × GA | | *P* (LRT) † |
|---|---|---|---|---|---|---|---|
| | Beta (SE) | *P* | Beta (SE) | *P* | Beta (SE) | *P* | |
| DD/other only (n=318*) | -0.12 (0.036) | 0.00075 | -0.20 (0.10) | 0.048 | 0.060 (0.036) | 0.089 | 0.095 |
| ADHD but not ASD (n=221*) | -0.12 (0.038) | 0.0014 | -0.24 (0.10) | 0.019 | 0.069 (0.035) | 0.050 | 0.034 |
| ASD (n=82*) | -0.14 (0.055) | 0.014 | -0.30 (0.16) | 0.058 | 0.090 (0.052) | 0.081 | 0.087 |
| Any NDD (n=621*) | -0.12 (0.030) | 0.00011 | -0.20 (0.081) | 0.012 | 0.060 (0.028) | 0.032 | 0.025 |
| NDD (number of conditions) | -0.034 (0.0092) | 0.00020 | -0.069 (0.026) | 0.0078 | 0.017 (0.0085) | 0.048 | 0.019 |

The effects (regression coefficient, beta) of gestational age (GA), mtDNA content z-score (mtCNz), their interaction (mtCNz × GA) on the risk of each of the three NDDs and any NDD from logistic regression, as well as on the number of NDD conditions from linear regression are shown. Two-sided *P* values are shown. The effect of mtCNz represents the effect estimated under the assumption that the gestational age at birth is 37 weeks.

*The number of cases used in each test.

†*P* values from the likelihood-ratio test (LRT) comparing the models with and without mtCNz and the interaction term. The null distribution of LRT statistic follows a chi-square distribution with 2 degrees of freedom.

## Supplementary Table 11. Associations of mtDNA heteroplasmies with NDD risks in the BBC.

| Group | min VAF | DD/other only (n=318) | | ADHD but not ASD (n=221) | | NDD but not ASD (n=539) | | Co-occurring DD & ADHD but not ASD (n=145) | |
|---|---|---|---|---|---|---|---|---|---|
| | | OR [95% CI] | *P* | OR [95% CI] | *P* | OR [95% CI] | *P* | OR [95% CI] | *P* |
| Predicted pathogenic | 1.5% | 1.20 [0.66-2.19] | 0.55 | 0.79 [0.37-1.70] | 0.54 | 0.99 [0.57-1.71] | 0.96 | 0.93 [0.40-2.19] | 0.87 |
| | 5% | 1.78 [0.62-5.06] | 0.28 | 1.20 [0.32-4.49] | 0.78 | 1.42 [0.53-3.79] | 0.48 | 1.93 [0.51-7.26] | 0.33 |
| | 10% | 4.13 [0.85-20.07] | 0.079 | 2.64 [0.41-17.16] | 0.31 | 3.19 [0.69-14.75] | 0.14 | 4.19 [0.64-27.51] | 0.14 |
| | 20% | 5.76 [0.67-49.80] | 0.11 | 4.58 [0.44-47.39] | 0.20 | 4.93 [0.61-39.79] | 0.13 | 7.21 [0.69-75.27] | 0.099 |
| OXPHOS & tRNA* | 1.5% | 1.13 [0.78-1.63] | 0.53 | 1.00 [0.65-1.55] | 0.98 | 1.08 [0.78-1.51] | 0.64 | 1.30 [0.80-2.09] | 0.29 |
| | 5% | 1.02 [0.59-1.77] | 0.95 | 1.19 [0.66-2.15] | 0.57 | 1.10 [0.68-1.78] | 0.68 | 1.61 [0.85-3.03] | 0.14 |
| | 10% | 1.55 [0.77-3.10] | 0.22 | 2.09 [1.00-4.38] | 0.049 | 1.78 [0.96-3.31] | 0.069 | 2.71 [1.23-5.96] | 0.014 |
| | 20% | 2.14 [0.88-5.18] | 0.092 | 2.75 [1.06-7.13] | 0.038 | 2.39 [1.06-5.36] | 0.035 | 3.95 [1.45-10.77] | 0.0074 |
| Others* | 1.5% | 1.07 [0.77-1.48] | 0.68 | 1.12 [0.77-1.62] | 0.56 | 1.07 [0.81-1.43] | 0.62 | 1.09 [0.71-1.68] | 0.69 |
| | 5% | 1.28 [0.82-1.99] | 0.28 | 1.52 [0.92-2.51] | 0.10 | 1.36 [0.91-2.02] | 0.13 | 1.34 [0.75-2.41] | 0.32 |
| | 10% | 1.14 [0.69-1.88] | 0.61 | 1.47 [0.84-2.57] | 0.18 | 1.24 [0.79-1.93] | 0.35 | 1.46 [0.77-2.78] | 0.24 |
| | 20% | 1.10 [0.60-2.00] | 0.76 | 1.00 [0.49-2.07] | 0.99 | 1.04 [0.61-1.79] | 0.88 | 0.97 [0.41-2.29] | 0.95 |

The four case groups are composed of children with a diagnosis of DD or other NDD only (n=318), ADHD but not ASD (n=221), any NDD but not ASD (n=539), or NDD with co-occurring DD and ADHD but not ASD (n=145), respectively. The odds ratios of carrying PP heteroplasmies for NDD were obtained from logistic regression in relation to 376 children with neurotypical development after adjustment for mtDNA content z-score (mtCNz). Participants carrying mtDNA heteroplasmies were determined based on the criterion of the minimum variant allele fraction (VAF) of the heteroplasmy at 1.5%, 5%, 10% and 20%. Two-sided *P* values are shown.

*Analyses were performed using mtDNA heteroplasmies at nonsynonymous sites (NS) in oxidative phosphorylation (OXPHOS) genes or at all sites in tRNA genes (OXPHOS & tRNA), and all other heteroplasmies in mtDNA (Others), respectively.

**Supplementary Table 12. Maternal-age-dependent accumulation of *de novo* heteroplasmies in mtDNA.**

| Study | Group | mtDNA (16569)* | | | OXPHOS & tRNA (10286)* | | |
|---|---|---|---|---|---|---|---|
| | | **Beta (SE)** | ***P*** | ***P* (het)†** | **Beta (SE)** | ***P*** | ***P* (het)†** |
| SSC | Sibling | 0.0099 (0.0029) | 0.00073 | - | 0.0048 (0.0019) | 0.013 | - |
| | Proband with ASD | 0.011 (0.0030) | 0.00015 | 0.74 | 0.0050 (0.0020) | 0.013 | 0.97 |
| BBC | Neurotypical (NT) child | 0.016 (0.0051) | 0.0015 | - | 0.011 (0.0030) | 0.00026 | - |
| | Child with NDD | 0.012 (0.0045) | 0.0082 | 0.52 | 0.0068 (0.0025) | 0.0069 | 0.29 |
| Meta‡ | Control (sibling & NT child) | 0.012 (0.0025) | 6.0E-06 | 0.28 | 0.0067 (0.0016) | 4.0E-05 | 0.086 |
| | Case (proband with ASD & child with NDD) | 0.011 (0.0025) | 3.6E-06 | 0.91 | 0.0057 (0.0016) | 0.00028 | 0.57 |
| | All (control & case) | 0.011 (0.0018) | 9.3E-11 | 0.76 | 0.0062 (0.0011) | 4.6E-08 | 0.33 |

*The effects (regression coefficient, beta) of maternal age at childbirth (years) on the number of *de novo* mtDNA heteroplasmies found in children and the *P* values (two-sided) were estimated using linear regression. Analyses were performed using *de novo* heteroplasmies at all mtDNA sites (mtDNA), as well as at nonsynonymous sites in oxidative phosphorylation genes or at all sites in tRNA genes (OXPHOS & tRNA). The number of sites shown in parentheses were averaged over all possible nucleotide changes.

†Q statistic *P* values for heterogeneity in the estimated regression coefficient (beta) between SSC and BBC samples or between control and case samples. The null distribution of Q statistic follows a chi-square distribution with 3 degrees of freedom in the group "All" and with 1 degree of freedom in other groups.

‡Results from fixed-effect meta-analysis.

**Supplementary Table 13. Germline mtDNA mutation rate estimated based on the observed number of *de novo* heteroplasmies in mtDNA.**

| | All mtDNA sites | OXPHOS & tRNA |
|---|---|---|
| No. of sites | 16569 | 10234* |
| Observed annual increase (No. of mutations) | 0.012 [0.0065 - 0.016] | 0.0067 [0.0035 - 0.0099] |
| Observed annual increase (mutations per site) | 6.9E-07 [3.9E-07 - 1.0E-06] | 6.6E-07 [3.5E-07 - 9.8E-07] |
| Mutation rate (per site per year) | 8.1E-09 [4.6E-09 - 1.2E-08] | 7.7E-09 [4.0E-09 - 1.1E-08] |
| Mutation rate (per site per generation) | 2.4E-07 [1.4E-07 - 3.5E-07] | 2.3E-07 [1.2E-07 - 3.4E-07] |

The annual mutation rate per site of mtDNA was estimated using the equation $u/s/(2 \times n \times \ln(1/f-1))$ from Rebolledo-Jaramillo *et al.*'s study[18]. *u* is the speed at which *de novo* heteroplasmies accumulate in mtDNA in the female germline for which we used the estimates obtained using controls from the SSC and BBC (Supplementary Table 12). *s* refers to the number of mtDNA sites; *n* is the number of segregating mtDNA units in the female germline for which we used the estimate of 10.3 from Zaidi *et al.*'s study[19]. *f* represents the minimum variant allele fraction of heteroplasmy which was 0.015 in the current study. We also used the average generation time of 30.2 years among control participants in the SSC and BBC to compute the mtDNA mutation rate per generation. The 95% confidence interval of the estimate is shown in brackets.

*The mtDNA mutation rate was estimated using all heteroplasmies, and heteroplasmies at nonsynonymous sites in oxidative phosphorylation genes or at all sites in tRNA genes (OXPHOS & tRNA), respectively. The number of sites shown were averaged over all possible nucleotide changes.

**Supplementary Table 14. Influence of pathogenicity on the inter-generational transmission of maternal mtDNA heteroplasmies among males and females.**

| Group | Female | | | Male | | |
|---|---|---|---|---|---|---|
| | N* | OR [95% CI] | *P* (het)† | N* | OR [95% CI] | *P* (het)† |
| Neurotypical‡ | 339 | 0.58 [0.35-0.94] | - | 972 | 0.58 [0.42-0.79] | - |
| ASD‡ | 154 | 0.18 [0.02-1.45] | 0.28 | 863 | 1.10 [0.80-1.52] | 0.0051 |
| ADHD but not ASD | 63 | 1.31 [0.38-4.57] | 0.23 | 158 | 1.39 [0.54-3.58] | 0.085 |
| DD/other only | 167 | 0.81 [0.31-2.17] | 0.54 | 151 | 1.15 [0.50-2.67] | 0.13 |
| Any NDD‡ | 384 | 0.80 [0.39-1.64] | 0.46 | 1172 | 1.13 [0.85-1.50] | 0.0021 |

The effects (regression coefficient, beta) of per standard deviation increase in the pathogenicity z-score on the inter-generation transmission of maternal heteroplasmies were estimated using logistic regression after adjustment for the location in mtDNA (OXPHOS or tRNA) and variant allele fraction of maternal heteroplasmies. Analyses were performed among females and males, separately. For the SSC, only data from families with the proband and the sibling of identical sex were used.

*The number of children used in each test.

†Q statistic *P* values for heterogeneity in the estimated OR of pathogenicity for heteroplasmy transmission among children with NDD relative to that among neurotypical children (as shown in the first row of the table). The null distribution of Q statistic follows a chi-square distribution with 1 degree of freedom.

‡Results from fixed-effect meta-analysis of the results from the SSC and BBC.

## Supplementary Table 15. Effects of PP heteroplasmies on ASD risk among males and females.

| Family (SSC) | Mother-child pair (BBC) | N* | Inherited PP heteroplasmy | | | PP heteroplasmy | | |
|---|---|---|---|---|---|---|---|---|
| | | | OR [95% CI] | *P* | *P* (het)† | OR [95% CI] | *P* | *P* (het)† |
| Female proband | Female child | 272/ 457 | 0.47 [0.07-3.14] | 0.43 | - | 0.97 [0.50-1.87] | 0.93 | - |
| Female proband & female sibling | Female child | 154/ 339 | NA | NA | - | 0.93 [0.42-2.06] | 0.86 | - |
| Male proband | Male child | 1748/ 1857 | 2.66 [1.41-5.04] | 0.0027 | 0.090 | 1.71 [1.29-2.26] | 0.00020 | 0.12 |
| Male proband & male sibling | Male child | 863/ 972 | 3.81 [1.48-9.85] | 0.0057 | 0.053 | 1.66 [1.13-2.44] | 0.0098 | 0.17 |

The odds ratios (ORs) of carrying PP heteroplasmies for ASD and the *P* values (two-sided) were obtained from fixed-effect meta-analysis of the results from the SSC and BBC with adjustment for mtDNA content z-score (mtCNz). Data from the families of the SSC and mother-child pairs from the BBC used in each test are indicated in the first two columns, respectively.

*Data are the number of cases/the number of controls from the SSC and BBC combined.

†Q statistic *P* values for heterogeneity in the estimated OR for ASD relative to that among female probands from the SSC and female newborns from the BBC (as shown in the first row of the table). The null distribution of Q statistic follows a chi-square distribution with 1 degree of freedom.

NA: not applicable, as the family-adjusted prevalence of PP heteroplasmies was zero among children with ASD.

## Supplementary Table 16. Effects of PP heteroplasmies on NDD risks among males and females in the BBC.

| NDD | Sex | N* | Inherited PP heteroplasmy | | | PP heteroplasmy (VAF≥5%) | | |
|---|---|---|---|---|---|---|---|---|
| | | | OR [95% CI] | *P* | *P* (het)† | OR [95% CI] | *P* | *P* (het)† |
| DD/other only | Female | 167 | 0.38 [0.04-3.74] | 0.41 | 0.063 | 0.22 [0.02-1.88] | 0.17 | 0.012 |
| | Male | 151 | 7.40 [0.88-62.44] | 0.066 | | 10.19 [1.25-82.76] | 0.030 | |
| ADHD but not ASD | Female | 63 | 1.15 [0.12-11.38] | 0.90 | 0.46 | NA | NA | NA |
| | Male | 158 | 3.90 [0.39-38.84] | 0.25 | | 5.08 [0.55-46.47] | 0.15 | |
| NDD but not ASD | Female | 230 | 0.57 [0.09-3.47] | 0.54 | 0.11 | 0.16 [0.02-1.37] | 0.095 | 0.011 |
| | Male | 309 | 5.46 [0.68-43.69] | 0.11 | | 7.55 [0.97-58.73] | 0.053 | |
| Co-occurring DD & ADHD but not ASD | Female | 40 | 1.91 [0.19-19.10] | 0.58 | 0.45 | NA | NA | NA |
| | Male | 105 | 6.72 [0.66-68.54] | 0.11 | | 8.68 [0.93-81.16] | 0.058 | |

The four case groups shown are composed of children with a diagnosis of DD or other NDD only, ADHD but not ASD, any NDD but not ASD, or NDD with co-occurring DD and ADHD but not ASD, respectively. The odds ratios (ORs) of carrying PP heteroplasmies for NDD and the *P* values (two-sided) were obtained from logistic regression in relation to 175 boys and 201 girls with neurotypical development after adjustment for mtDNA content z-score (mtCNz).

*The number of cases used in each test.

†Q statistic *P* values for heterogeneity in the estimated odds ratios between males and females. The null distribution of Q statistic follows a chi-square distribution with 1 degree of freedom.

NA: not applicable, as the family-adjusted prevalence of PP heteroplasmies was zero among girls with ADHD.

## Supplementary Table 17. Prevalence of PP heteroplasmies among males and females.

| Study | Group | N* | Among all participants† | | Among heteroplasmy carriers† | |
|---|---|---|---|---|---|---|
| | | | Case | Control | Case | Control |
| Family (SSC) | Female proband & female sibling | 138/ 138 | 5.80% | 8.70% | 12.90% | 19.05% |
| | Female proband & male sibling | 118/ 118 | 7.63% | 5.08% | 16.98% | 10.91% |
| | Male proband & male sibling | 797/ 797 | 8.66% | 5.40% | 18.65% | 12.32% |
| Mother-child pair (BBC) | Female child | 246/ 201 | 4.07% | 6.47% | 9.35% | 14.77% |
| | Male child | 375/ 175 | 9.07% | 5.71% | 18.58% | 13.70% |

*Data are the number of cases/the number of controls. Cases refer to probands with ASD in the SSC or children with any NDD in the BBC. Controls refer to siblings in the SSC or children with neurotypical development in the BBC.
†Data are the family-adjusted prevalence of predicted pathogenic (PP) heteroplasmies among cases and controls and among cases and controls carrying mtDNA heteroplasmies.

## Supplementary Table 18. Effects of PP heteroplasmies on ASD risk among European and non-European participants.

| Ethnicity/race* | N† | PP heteroplasmy | | |
|---|---|---|---|---|
| | | OR [95% CI] | *P* | *P* (het)‡ |
| European | 1391/1396 | 1.49 [1.08-2.04] | 0.014 | - |
| Others: | 629/918 | 1.68 [1.08-2.62] | 0.022 | 0.66 |
| African American | 94/279 | 2.47 [0.83-7.37] | 0.10 | 0.38 |
| Latino, Asian, Native American, Mixed-race, etc. | 535/639 | 1.61 [0.99-2.64] | 0.057 | 0.79 |

The odds ratios (ORs) of carrying PP heteroplasmies for ASD and the *P* values (two-sided) were obtained from fixed-effect meta-analysis of the results from the SSC and BBC after adjustment for mtDNA content z-score (mtCNz).

*Analyses were performed using a subset of SSC and BBC samples: (1) families with both parents in the SSC or the child in the BBC reporting European ancestry (European); (2) families with either parent in the SSC or the child in the BBC not of European ancestry (Others); (3) families with both parents in the SSC or the child in the BBC reporting African ancestry (African American); and (4) families with parents reporting different races/ethnicities, or being of Latino, Asian, or Native American ancestry, or being mixed race in the SSC, or the child of Latino or Native American ancestry or being mixed race in the BBC (Latino, Asian, Native American, Mixed-race, etc.).

†Data are the number of cases/the number of controls from the SSC and BBC combined.

‡Q statistic *P* values for heterogeneity in the estimated OR for ASD as compared to that among European participants. The null distribution of Q statistic follows a chi-square distribution with 1 degree of freedom.

**Supplementary Table 19. Effects of PP heteroplasmies in OXPHOS genes and RNA genes, respectively, as well as disease-associated PP heteroplasmies on ASD risk.**

| Group | PP heteroplasmy | | PP heteroplasmy (VAF≥5%) | |
|---|---|---|---|---|
| | **OR [95% CI]** | ***P*** | **OR [95% CI]** | ***P*** |
| OXPHOS | 1.56 [1.16-2.11] | 0.0037 | 2.16 [1.34-3.48] | 0.0016 |
| RNA | 1.61 [1.02-2.53] | 0.040 | 2.83 [1.07-7.46] | 0.036 |
| Disease-associated (all)* | 1.88 [1.17-3.05] | 0.0096 | 2.67 [1.04-6.90] | 0.042 |
| Disease-associated (neurological)* | 2.78 [1.44-5.34] | 0.0022 | 3.62 [1.00-13.05] | 0.049 |

The odds ratios (ORs) of carrying predicted pathogenic (PP) heteroplasmies for ASD and the *P* values (two-sided) were obtained from fixed-effect meta-analysis of the results from the SSC and BBC after adjustment for mtDNA content z-score (mtCNz).

*PP heteroplasmies that were reported to be associated with a disease (marked as "reported" or "confirmed") in the MITOMAP database or were marked as "likely pathogenic" or "pathogenic" in the ClinVar database. Diseases that have a neurological manifestation were determined as described in Supplementary Figure 3.

**Supplementary Table 20. Characteristics of nucleotide changes among *de novo* heteroplasmies in children.**

| Nucleotide change | | SSC | | | BBC | | |
|---|---|---|---|---|---|---|---|
| | | **Sibling*** | **Proband*** | ***P*†** | **Child (NT)*** | **Child (NDD)*** | ***P*†** |
| Transition (Ti) | All Ti | 97.0 | 96.9 | 1.00 | 97.2 | 98.1 | 0.73 |
| | A>G | 24.7 | 22.8 | 0.39 | 23.1 | 23.0 | 1.00 |
| | G>A | 27.7 | 29.5 | 0.45 | 28.7 | 24.5 | 0.41 |
| | C>T | 12.4 | 11.0 | 0.41 | 12.6 | 13.2 | 1.00 |
| | T>C | 32.2 | 33.6 | 0.58 | 32.9 | 37.4 | 0.39 |
| Transversion (Tv) | All Tv | 3.0 | 3.1 | 1.00 | 2.8 | 1.9 | 0.73 |
| | A>C | 0.8 | 0.4 | 0.33 | 0.7 | 0.4 | 1.00 |
| | C>A | 0.6 | 0.7 | 1.00 | 0.7 | 0.8 | 1.00 |
| | A>T | 0.7 | 0.5 | 0.75 | 0.7 | 0.0 | 0.35 |
| | T>A | 0.1 | 0.8 | 0.12 | 0.0 | 0.0 | 1.00 |
| | C>G | 0.0 | 0.0 | 1.00 | 0.0 | 0.4 | 1.00 |
| | G>C | 0.1 | 0.5 | 0.37 | 0.7 | 0.0 | 0.35 |
| | G>T | 0.1 | 0.0 | 0.49 | 0.0 | 0.4 | 1.00 |
| | T>G | 0.4 | 0.1 | 0.36 | 0.0 | 0.0 | 1.00 |

*Data are the percentage (%) of each type of nucleotide changes (from the reference allele to [>] the variant allele as indicated by the labels in the second column) among *de novo* mtDNA heteroplasmies detected in siblings and probands in the SSC as well as neurotypical children and children with NDD in the BBC, respectively. The value shown is rounded to the nearest tenth. The percentages of all types of nucleotide changes may not add up to 100.

†*P* values (two-sided) from the Fisher's exact test.

## Supplementary Table 21. Flow chart of whole-genome sequencing data analysis in the SSC.

| | Step | Description* |
|---|---|---|
| 1 | **Parallel sequencing** | Download alignment files generated from whole-genome sequencing (WGS) for 7,768 participants in the Simons Simplex Collection (SSC). Paired-end reads in the alignment files are `bwa mem` aligned to the complete human genome (genome assembly GRCh38). |
| 2 | **Complete genome alignment** | Extract read pairs mapped to mtDNA or known nuclear mitochondrial DNA segments in the downloaded alignment files.<br>Steps 1 & 2:<br>**pulldata.py alignment_file_link -r rCRS_chrM_numts.hg38.cord** |
| 3 | **Mitochondrial genome alignment** | Map extracted reads that are aligned to mtDNA with mapping quality (MAPQ) ≥20 to a modified mtDNA with the final 150-bp of Revised Cambridge Reference Sequence (rCRS) of human mtDNA copied to the start:<br>**bwa mem -M rCRS_chrM_16419-1-16569.fa** |
| 4 | **Read calibration** | Identify read duplicates using Picard:<br>**java -jar picard.jar MarkDuplicates**<br>Perform local re-alignment and base quality recalibration:<br>**bamleftalign -f \| samtools calmd -EArb** |
| 5 | **Read filtering** | Retain only primary unique read pairs mapped to the modified mtDNA.<br>Reads are in a proper pair if not in the circular D-loop region.<br>Reads contain less than 5% nucleotide mismatches relative to rCRS. |
| 6 | **Base summarization** | Pile up retained reads against the reference mtDNA:<br>**samtools mpileup -q 20 -Q 0 -B -d 500000 -f rCRS_chrM_16419-1-16569.fa**<br>Restore mtDNA coordinates to the original coordinates of rCRS.<br>Steps 3-6:<br>**stamp.py wgs-align --mtdna rCRS_chrM_16419-1-16569.fa --mtdna-offset 150 **<br>**--realign-recal** |
| 7 | **Quality assessment** | Summarize read depth and base information for all sites of mtDNA and compute variant allele fractions and quality for sites having variant allele fraction≥0.001 among unique reads with base alignment quality (BAQ) ≥30 and at least three variant alleles detected. The variant allele is also required to be detectable on both strands of mtDNA.<br>**stamp.py scan --min-qual 30 --minh 3 --minh-fwd 1 --minh-rev 1 --min-het 0.001 **<br>**--all-sites** |
| 8 | **mtDNA content assessment** | Count the number of unique and properly paired reads in 100-kb sliding windows across the nuclear genome with step size of 50-kb, and then compute the average depth of read coverage on each chromosome based on windows with >80% sites covered with reads.<br>**stamp.py wgs-depth --proper-pair --qual-filter ' -q16 -Q20 '**<br>**-r GRCh38_full_analysis_set_plus_decoy_hla.fa **<br>**--win-size 100000 --sliding-size 50000 --min-sites 0.8**<br>Compute mtDNA content as two times the average read depth in the coding region of mtDNA (m.577-m.16023) divided by the average read depth in each of the 22 autosomal chromosomes:<br>$\frac{2}{22}\sum_{i=1}^{22}\frac{average\ read\ depth\ in\ the\ coding\ region\ of\ mtDNA}{\frac{1}{no.\ of\ windows}\sum average\ read\ depth\ in\ 100kb\ sliding\ windows\ across\ chromosome\ i}$ |

*Command and parameters of the software or the computational pipeline used are shown in bold type.

**Supplementary Table 22. Flow chart of mtDNA-targeted sequencing data analysis in the BBC.**

| Step | | Description* |
|---|---|---|
| 1 | **Parallel sequencing** | Make mtDNA-enriched sequencing libraries from genomic DNA samples collected in the Boston Birth Cohort (BBC) and then sequence STAMP libraries on Illumina HiSeq 2500 with 2×250-bp reads. |
| 2 | **Complete genome alignment** | Sort paired-end reads based on the sequences of probe and sample barcode; trim molecular barcode and probe sequences from each read pair before complete genome alignment:<br>**bwa mem -L 100, 5 -M GRCh38_full_analysis_set_plus_decoy_hla.fa**<br>"-L 100,5" disables soft clips following the trimmed probe arm sequences.<br>Mark reads that contain mtDNA probe sequences but are aligned to nuclear DNA with mapping quality (MAPQ) ≥10 as nuclear mitochondrial DNA segment (NUMTS). |
| 3 | **Mitochondrial genome alignment** | Map all reads again to a modified mtDNA with the final 120-bp of Revised Cambridge Reference Sequence (rCRS) of human mtDNA copied to the start:<br>**bwa mem -L 100, 5 -M rCRS_chrM_16449-1-16569.fa**<br>Remove reads that have incorrect alignment locations and strand information. |
| 4 | **Read calibration** | Perform local re-alignment and base quality recalibration:<br>**bamleftalign -f \| samtools calmd -EArb**<br>Generate a consensus read from paired-end reads containing the same molecular barcode.<br>Steps 2-4:<br>**stamp.py align -p probe.txt --mtdna rCRS_chrM_16449-1-16569.fa **<br>**--mtdna-offset 120 --genome GRCh38_full_analysis_set_plus_decoy_hla.fa** |
| 5 | **Read filtering** | Filter consensus reads according to a list of NUMTS sequences and then remove consensus reads with an excess of nucleotide mismatches (>5 in the coding region and >8 in the D-loop region) relative to the major mtDNA sequence of each sample. |
| 6 | **Base summarization** | Pile up consensus reads retained against the reference mtDNA:<br>**samtools mpileup -q 20 -Q 0 -B -d 500000 -f rCRS_chrM_16449-1-16569.fa**<br>Restore mtDNA coordinates to the original coordinates of rCRS.<br>Steps 5 & 6:<br>**stamp.py pileup -p probe.txt --mtdna rCRS_chrM_16449-1-16569.fa **<br>**--mtdna-offset 120 --nm-max 5 --nm-max-dloop 8 --numts-excl numts_list.rCRS.hsd**<br>Also summarize base information in consensus reads that are constructed with single paired-end reads:<br>**stamp.py pileup … --fs-max 1**<br>or multiple paired-end reads:<br>**stamp.py pileup … --fs-min 2** |
| 7 | **Quality assessment** | Summarize read depth and base information for all sites of mtDNA and compute variant allele fractions and quality for sites having variant allele fraction ≥0.001 among consensus reads with base alignment quality (BAQ) ≥30 and at least three variant alleles detected:<br>**stamp.py scan --min-qual 30 --minh 3 --min-het 0.001 --all-sites**<br>Compare variant allele fractions estimated from consensus reads constructed with single paired-end reads and from consensus reads constructed with multiple paired-end reads:<br>**Rscript summarize_variation_freq.R all.var single.var multiple.var** |
| 8 | **mtDNA content assessment** | Count the number of consensus reads from the three target single-copy nuclear DNA (nDNA) regions on chromosomes 8, 14, and 19; count the number of consensus reads aligned to the target regions of probes (“A2”, “A3”, “A7”, “C9”, “D1” and “D5”) whose annealing sites in mtDNA do not overlap with polymorphisms with frequency >0.2% among BBC samples:<br>**summarize_reads.py**<br>Estimate mtDNA content using a residual method in a linear model:<br>$\log_2$(no. of mtDNA consensus reads) ~ $\log_2$(no. of nDNA consensus reads) + sample_plate |

*Command and parameters of the software or the computational pipeline used are shown in bold type.

**Supplementary Table 23. Flow chart of mtDNA heteroplasmy detection in the SSC and BBC.**

| Step | | Description* |
|---|---|---|
| 1 | **Filtering based on variant quality** | **All mtDNA variants identified are further filtered according to criteria i-vii:**<br>**(i)** >500-fold (X) unique/consensus reads with base alignment quality (BAQ) ≥30.<br>**(ii)** >70% of the unique/consensus reads having BAQ≥30.<br>**(iii)** not in low-complexity regions of mtDNA (i.e., m.302-m.316, m.512-m.526, m.16814-m.16193) or at sites associated with the most prevalent polymorphic NUMTS (i.e., m.12684, m.12705).<br>**(iv)** ≥5 minor alleles (BAQ≥30) and log-likelihood BAQ-based quality score >5.<br>**(v)** SSC: nonzero and comparable allele fractions among unique reads mapped to the forward strand and to the reverse strand of mtDNA (Fisher's exact test $P \geq 10^{-4}$).<br>**(vi)** BBC: variant allele fractions (VAF) among consensus reads constructed from multiple paired-end reads comparable to that among reads constructed from single paired-end reads (Fisher's exact test $P \geq 10^{-4}$ and decrease in VAF <5-fold).<br>**(vii)** BBC: not at low-quality sites defined as those having >50% of variants (with VAF≥1%) failed in quality filter **vi**. |
| 2 | **Filtering based on variant fraction** | **Determine heteroplasmic sites among participants from the same family.**<br>Sites of medium-to-high-fraction heteroplasmies: with VAF between 0.05 and 0.95.<br>Sites of low-to-medium-fraction heteroplasmies: with VAF as well as the maximum likelihood estimation of the minor allele fraction from the BAQ-based quality test ≥0.015. |
| 3 | **Assessing variant sharing among sample pairs** | **Search for variant alleles in all participants from the same family, and determine secondary heteroplasmies at heteroplasmic sites according to:**<br>**(i)** >500X unique/consensus reads (BAQ≥30).<br>**(ii)** fraction of the variant allele ≥0.2%.<br>**(iii)** probability of observing more errors than the variant allele $<10^{-3}$ (*Poisson* exact test; error rate, ε=0.2%).<br>**(iv)** SSC: the variant allele detected on both strands of mtDNA. |
| **Number of mtDNA heteroplasmies Identified** | | SSC: 5,628 mtDNA heteroplasmies in 1,938 families (consisting of both parents, probands and siblings), including 615 secondary heteroplasmies.<br>BBC: 1,574 mtDNA heteroplasmies in 997 mother-child pairs, including 109 secondary heteroplasmies. |
| 4 | **Evaluating pathogenicity of mtDNA heteroplasmies** | **Inclusion and exclusion criteria for predicted pathogenic (PP) heteroplasmies:**<br>**Inclusion criteria (i-iii)**:<br>**(i)** Nonsynonymous variants that are consistently predicted to be pathogenic by CADD (Phred score>15), PolyPhen-2 (possibly or probably damaging), and MutPred (score>0.6) in OXPHOS genes.<br>**(ii)** variants predicted to be pathogenic (MitoTIP raw score>12.66) in tRNA genes.<br>**(iii)** confirmed mitochondrial disorder (MD)-causing point mutations listed in the MITOMAP database.<br>**Exclusion criteria (iv-v)**:<br>**(iv)** variants with an annotation of "benign" listed in the ClinVar database.<br>**(v)** variants with a frequency >0.05% among apparently healthy participants in HmtDB or among participants in the reference populations of gnomAD. |

*Filters and results specific to the SSC or BBC are indicated by the respective study label.

## Supplementary Table 24. Information of the extension-ligation Probes and the target regions used in STAMP.

CHR: chromosome; Strand: ligation arm strand /extension arm strand; Barcode: maximum barcode length.

| Probe ID | CHR | Target region (start-end) | Strand | Barcode | Ligation arm sequence / Extension arm sequence |
|---|---|---|---|---|---|
| A1 | chrM | 311-753 | +/- | 15 | CTGTGGCCAGAAGCGGGG / TGTTCCTTTTGATCGTGGTGATTTA |
| A2 | chrM | 701-1141 | -/+ | 15 | AACAAAACTGCTCGCCAGAAC / CATCCCCGTTCCAGTGAGTT |
| A3 | chrM | 1095-1535 | +/- | 15 | TTTAACTGTTGAGGTTTAGGGCT / AGGGGTTTTAGTTAAATGTCCTTTG |
| A4 | chrM | 1474-1911 | -/+ | 12 | CCAAAGCTAAGACCCCCGAAACC / CGTACACACCGCCCGTCAC |
| A5 | chrM | 1854-2283 | +/- | 15 | TTGCAAAGTTATTTCTAGTTAATTCATT / GGTGATAGATTGGTCCAATTGG |
| A6 | chrM | 2206-2646 | -/+ | 15 | TGTATGAATGGCTCCACGAGG / AAGCTCAACACCCACTACCT |
| A7 | chrM | 2538-2980 | +/- | 12 | TGTCACTGGGCAGGCGGT / AAACCCTATTGTTGATATGGACTCT |
| A8 | chrM | 2932-3377 | -/+ | 15 | ATGGCATTCCTAATGCTTACCGA / GGATAACAGCGCAATCCTATTCT |
| A9 | chrM | 3313-3753 | +/- | 15 | AGGAGTAGGAGGTTGGCCA / AATGATGGCTAGGGTGACTTCA |
| A10 | chrM | 3687-4128 | -/+ | 15 | ACCTCCCTGTTCTTATGAATTCGA / GCCCTGATCGGCGCACTG |
| A11 | chrM | 4022-4471 | +/- | 15 | ATATGTTGTTCCTAGGAAGATTGTA / ATTAGTACGGGAAGGGTATAACCAA |
| A12 | chrM | 4348-4797 | -/+ | 15 | GCTATAGCAATAAAACTAGGAATAGCCC / CCCATCCCTGAGAATCCAAAAT |
| B1 | chrM | 4705-5150 | +/- | 12 | TGGTTCATTGTCCGGAGAGT / GGTCGTGGTGCTGGAGTTTA |
| B2 | chrM | 5099-5498 | -/+ | 15 | CTCCTACCTATCTCCCCTTTTATA / CTAACTACTACCGCATTCCTACTAC |
| B3 | chrM | 5453-5896 | +/- | 15 | AGCGTGGTAAGGGCGATGAG / GGTGAGGTAAAATGGCTGAGTG |
| B4 | chrM | 5843-6286 | -/+ | 15 | GCAGGAACAGGTTGAACAGT / CCCCTGTCTTTAGATTTACAGTCC |
| B5 | chrM | 6218-6658 | +/- | 15 | CAGGAGTAGGAGAGAGGGAGG / CCGAAGCCTGGTAGGATAAGAA |
| B6 | chrM | 6514-6960 | -/+ | 15 | TTTCTTTTCACCGTAGGTGGCC / TGGCATCACTATACTACTAACAGAC |
| B7 | chrM | 6914-7357 | +/- | 15 | AATCCTAGGGCTCAGAGCAC / ACTATTAGGACTTTTCGCTTCGAA |
| B8 | chrM | 7232-7676 | -/+ | 12 | TTTCATGATCACGCCCTCATAATCA / CCCGATGCATACACCACATGA |
| B9 | chrM | 7608-8036 | +/- | 15 | GGGGAAGTAGCGTCTTGTAGA / GAATGGGGGCTTCAATCGGG |
| B10 | chrM | 7882-8297 | -/+ | 15 | STACCCCCTCTAGAGCCCAC / ATTGGCCACCAATGGTACTGA |
| B11 | chrM | 8249-8666 | +/- | 15 | TGCTATAGGGTAAATACGGGCC / TGTTGGGTGGTGATTAGTCGG |
| B12 | chrM | 8544-8968 | -/+ | 15 | CCCATACTAGTTATTATCGAAACCATCA / GCTTCATTCATTGCCCCCAC |
| C1 | chrM | 8912-9346 | +/- | 15 | GTGTAGGTGTGCCTTGTGGT / AGGCCTAGTATGAGGAGCGT |

| | | | | | |
|---|---|---|---|---|---|
| C2 | chrM | 9274-9695 | -/+ | 15 | CCGAAACCAAATAATTCAAGCACTG / AGCCCTCCTAATGACCTCCG |
| C3 | chrM | 9500-9940 | +/- | 15 | GCTGGAGTGGTAAAAGGCTCA / ACAAAATGCCAGTATCAGGCG |
| C4 | chrM | 9853-10293 | -/+ | 15 | CTTTTACCCCTACCATGAGCCC / TATCTGCTTCATCCGCCAACTA |
| C5 | chrM | 10209-10648 | +/- | 15 | ATAGCTACTAAGAAGAATTTTATGGAG A / GGCACAATATTGGCTAAGAGGG |
| C6 | chrM | 10600-11022 | -/+ | 15 | CGCCACTTATCCAGTGAACC / TACTCTCATAACCCTCAACACCC |
| C7 | chrM | 10926-11368 | +/- | 12 | GTTAGGGGGTCGGAGGAA / AAAAGCTATTGTGTAAGCTAGTCAT |
| C8 | chrM | 11298-11744 | -/+ | 15 | CTTACATCCTCATTACTATTCTGCC / TCTCACTGCCCAAGAACTATCA |
| C9 | chrM | 11687-12127 | +/- | 15 | ATTATGAGAATGACTGCGCCGG / CCCGGTAATGATGTCGGGG |
| C10 | chrM | 12075-12502 | -/+ | 15 | CTCTTCCCCACAACAATATTCATGT / ACACCTATCCCCCATTCTCCT |
| C11 | chrM | 12448-12894 | +/- | 12 | ATAATAAAGGTGGATGCGACAATGG / TAAGGCGAGGATGAAACCGATA |
| C12 | chrM | 12823-13270 | -/+ | 15 | CTCCACTTCAAGTCAACTAGGAC / ATGCCAACACAGCAGCCATT |
| D1 | chrM | 13214-13658 | +/- | 15 | ACGATTTTTTTGATGTCATTTTGTGTA / GTAAGGGTGGGGAAGCGA |
| D2 | chrM | 13592-14008 | -/+ | 15 | ACTCCTCCTAGACCTAACCTGAC / AAGCGCCTATAGCACTCGAA |
| D3 | chrM | 13955-14396 | +/- | 15 | TTTTGGCTCGTAAGAAGGCCT / TAGTGGGGTTAGCGATGGAGG |
| D4 | chrM | 14348-14773 | -/+ | 15 | CCCAATACGCAAAACTAACCCC / ACTCTTTCACCCACAGCACC |
| D5 | chrM | 14719-15163 | +/- | 15 | GTGTTCTTGTAGTTGAAATACAACG / TGATATTTGGCCTCACGGGA |
| D6 | chrM | 15092-15535 | -/+ | 15 | TATACCCTAGCCAACCCCTTAAAC / GCATTATCCTCCTGCTTGCA |
| D7 | chrM | 15353-15778 | +/- | 15 | TAGGGGGTTGTTTGATCCCG / GCTTACTGGTTGTCCTCCGAT |
| D8 | chrM | 15733-16178 | -/+ | 12 | TAGTACATAAAAACCCAATCCACAT / GCAGACCTCCTCATTCTAACC |
| D9 | chrM | 16112-16561 | +/- | 15 | TTTATGGTACCGTACAATATTCATGGT / TGTCTTATTTAAGGGGAACGTGT |
| D10 | chrM | 16499-358 | -/+ | 15 | CACATCTCTGCCAAACCCCAA / ATCTGGTTCCTACTTCAGGGTC |
| EMC1 | chr1 | 19563534-19563970 | +/- | 15 | GTTTTGGGCCGTCAGAGGAT / AAGGAAAACCGGACTTCGCA |
| WRN | chr8 | 33162768-33163207 | -/+ | 15 | TGTTTGAAGTCTTGGTTTGGTG / GAAGATAATGGGATTCAGAACTCAG |
| SERPINA1 | chr14 | 94439180-94439625 | -/+ | 15 | GTGCAGCCTTCATGGTTTCG / CCACAGAGAGCATCGCAAGA |
| B2M | chr15 | 44723613-44724059 | -/+ | 15 | TTGTCTGTGATGTAGCCATCA / TGTCAACAACCACTTTCACGG |
| AXL | chr19 | 40978400-40978829 | +/- | 15 | GAATAGCACTCCTCCACAGGG / TCTACCTGAATTCTGGAACAGCCG |

The start and end positions of the target regions are shown with those in rCRS (the reference mtDNA) and nuclear genome (assembly GRCh38). The ligation arm of B10 was designed with a degenerate base S (G/C) to match the mtDNA sequence with an 8271-8279 or 8281-8289 deletion (i.e., in the Asian mtDNA haplogroups B2 or B4). Probes were column-synthesized at 25 nanomole scale with standard desalting purification (Integrated DNA Technologies). The relative amount of the 51 probes in the mix was determined empirically[20].

## Supplementary References


1. Li, M. *et al.* The association of maternal obesity and diabetes with autism and other developmental disabilities. *Pediatrics* **137**, e20152206 (2016).

2. Panjwani, A. A. *et al.* Maternal obesity/diabetes, plasma branched-chain amino acids, and autism spectrum disorder risk in urban low-income children: evidence of sex difference. *Autism Res.* **12**, 1562–1573 (2019).

3. Ji, Y. *et al.* Association of cord plasma biomarkers of in utero acetaminophen exposure With risk of attention-deficit/hyperactivity disorder and autism Spectrum disorder in childhood. *JAMA psychiatry* **77**, 180–189 (2020).

4. Hong, X. *et al.* Genome-wide approach identifies a novel gene-maternal pre-pregnancy BMI interaction on preterm birth. *Nat. Commun.* **8**, 15608 (2017).

5. Dayama, G., Emery, S. B., Kidd, J. M. & Mills, R. E. The genomic landscape of polymorphic human nuclear mitochondrial insertions. *Nucleic Acids Res.* **42**, 12640–12649 (2014).

6. Wang, Y., Guo, X., Ye, K., Orth, M. & Gu, Z. Accelerated expansion of pathogenic mitochondrial DNA heteroplasmies in Huntington's disease. *Proc. Natl. Acad. Sci. U. S. A.* **118**, e2014610118 (2021).

7. Just, R. S., Irwin, J. A. & Parson, W. Mitochondrial DNA heteroplasmy in the emerging field of massively parallel sequencing. *Forensic Sci. Int. Genet.* **18**, 131–139 (2015).

8. Wang, Y., Picard, M. & Gu, Z. Genetic evidence for elevated pathogenicity of mitochondrial DNA heteroplasmy in autism spectrum disorder. *PLoS Genet.* **12**, e1006391 (2016).

9. Kircher, M. *et al.* A general framework for estimating the relative pathogenicity of human genetic variants. *Nat. Genet.* **46**, 310–5 (2014).

10. Sonney, S. *et al.* Predicting the pathogenicity of novel variants in mitochondrial tRNA with MitoTIP. *PLoS Comput. Biol.* **13**, 1–8 (2017).

11. Adzhubei, I., Jordan, D. M. & Sunyaev, S. R. Predicting functional effect of human missense mutations using PolyPhen-2. *Curr. Protoc. Hum. Genet.* **76**, 7.20.1-7.20.41 (2013).

12. Li, B. *et al.* Automated inference of molecular mechanisms of disease from amino acid substitutions. *Bioinformatics* **25**, 2744–50 (2009).

13. Lott, M. T. *et al.* mtDNA variation and analysis using Mitomap and Mitomaster. *Curr. Protoc. Bioinforma.* **44**, 1.23.1-26 (2013).

14. Landrum, M. J. *et al.* ClinVar: public archive of relationships among sequence variation and human phenotype. *Nucleic Acids Res.* **42**, D980-5 (2014).

15. Rubino, F. *et al.* HmtDB, a genomic resource for mitochondrion-based human variability studies. *Nucleic Acids Res.* **40**, D1150–D1159 (2012).

16. Karczewski, K. J. *et al.* The mutational constraint spectrum quantified from variation in 141,456 humans. *Nature* **581**, 434–443 (2020).

17. Weissensteiner, H. *et al.* HaploGrep 2: mitochondrial haplogroup classification in the era of high-throughput sequencing. *Nucleic Acids Res.* gkw233 (2016). doi:10.1093/nar/gkw233

18. Rebolledo-Jaramillo, B. *et al.* Maternal age effect and severe germ-line bottleneck in the inheritance of human mitochondrial DNA. *Proc. Natl. Acad. Sci.* **111**, 15474–15479 (2014).

19. Zaidi, A. A. *et al.* Bottleneck and selection in the germline and maternal age influence transmission of mitochondrial DNA in human pedigrees. *Proc. Natl. Acad. Sci. U. S. A.* **116**, 25172–25178 (2019).

20. Guo, X., Wang, Y., Zhang, R. & Gu, Z. STAMP: a multiplex sequencing method for simultaneous evaluation of mitochondrial DNA heteroplasmies and content. *NAR Genomics Bioinforma.* **2**, lqaa065 (2020).
