# Supplementary material for: Association of mitochondrial DNA content, heteroplasmies and inter-generational transmission with autism
Source: Nat Commun. 2022 Jul 1;13:3790. doi: 10.1038/s41467-022-30805-7 (PMC9249801; doi:10.1038/s41467-022-30805-7)
Supplement: Supplementary file 2 — Description of Additional Supplementary Files [file 41467_2022_30805_MOESM2_ESM.pdf]

File name: Supplementary Data 1

Description: mtDNA heteroplasmies identified in the SSC.
