# Supplementary material for: Association of mitochondrial DNA content, heteroplasmies and inter-generational transmission with autism
Source: Nat Commun. 2022 Jul 1;13:3790. doi: 10.1038/s41467-022-30805-7 (PMC9249801; doi:10.1038/s41467-022-30805-7)
Supplement: Supplementary file 4 — Reporting Summary [file 41467_2022_30805_MOESM4_ESM.pdf]

Corresponding author(s): Xiaobin Wang; Zhenglong Gu

Last updated by author(s): Apr 22, 2022

Reporting Summary

Nature Portfolio wishes to improve the reproducibility of the work that we publish. This form provides structure for consistency and transparency in reporting. For further information on Nature Portfolio policies, see our Editorial Policies and the Editorial Policy Checklist.

Statistics

For all statistical analyses, confirm that the following items are present in the figure legend, table legend, main text, or Methods section.

n/a	Confirmed	
☐	☒	The exact sample size (*n*) for each experimental group/condition, given as a discrete number and unit of measurement
☐	☒	A statement on whether measurements were taken from distinct samples or whether the same sample was measured repeatedly
☐	☒	The statistical test(s) used AND whether they are one- or two-sided<br>*Only common tests should be described solely by name; describe more complex techniques in the Methods section.*
☐	☒	A description of all covariates tested
☐	☒	A description of any assumptions or corrections, such as tests of normality and adjustment for multiple comparisons
☐	☒	A full description of the statistical parameters including central tendency (e.g. means) or other basic estimates (e.g. regression coefficient) AND variation (e.g. standard deviation) or associated estimates of uncertainty (e.g. confidence intervals)
☐	☒	For null hypothesis testing, the test statistic (e.g. *F*, *t*, *r*) with confidence intervals, effect sizes, degrees of freedom and *P* value noted<br>*Give P values as exact values whenever suitable.*
☒	☐	For Bayesian analysis, information on the choice of priors and Markov chain Monte Carlo settings
☒	☐	For hierarchical and complex designs, identification of the appropriate level for tests and full reporting of outcomes
☐	☒	Estimates of effect sizes (e.g. Cohen's *d*, Pearson's *r*), indicating how they were calculated

Our web collection on statistics for biologists contains articles on many of the points above.

Software and code

Policy information about availability of computer code

Data collection	No software was used for data collection.
Data analysis	Software used for processing sequencing data:<br>SAMtools v1.6, BWA-mem v0.7.17, Picard v1.138, freebayes v1.1, HaploGrep 2 v2.1.1.<br>stamp (custom code available at https://github.com/mtstamp/stamp; last access on Aug 11, 2021)<br><br>Software used for variant annotation:<br>ANNOVAR (version: $Date: 2015-06-17 21:43:51 -0700 [Wed, 17 Jun 2015] $)<br>CADD v1.3, PolyPhen-2 v2.2.2, MutPred (mtDNA variants extracted from Pereira et al. AJHG. 2011), MitoTIP (available at https://www.mitomap.org/downloads/mitotip_scores.txt; last access on Feb 08, 2020; from Sonney et al. PLoS Comput. Biol. 2017)<br><br>Software used for statistical analysis:<br>R (version 3.5.0; package survival [version 2.41-3] used for conditional logistic regression)

For manuscripts utilizing custom algorithms or software that are central to the research but not yet described in published literature, software must be made available to editors and reviewers. We strongly encourage code deposition in a community repository (e.g. GitHub). See the Nature Portfolio guidelines for submitting code & software for further information.

Data

Policy information about availability of data

All manuscripts must include a data availability statement. This statement should provide the following information, where applicable:

- Accession codes, unique identifiers, or web links for publicly available datasets
- A description of any restrictions on data availability
- For clinical datasets or third party data, please ensure that the statement adheres to our policy

The data on mtDNA heteroplasmies of the SSC generated in this study are provided in the Supplementary Data file (Supplementary Data 1). The raw whole-genome sequencing data and phenotype data of the SSC used in this study are available in the SFARI Base (https://base.sfari.org/) under Resource: SSC Whole-genome 2 and Simons Simplex Collection The related phenotype data of the SSC are also available in NADR database under collections 2042 and 2068 (https://nda.nih.gov/edit_collection.html?id=2042; https://nda.nih.gov/edit_collection.html?id=2068). The data of the BBC are only available under restricted access due to the informed consent and Institutional Review Board (IRB) guidance of the BBC which is an ongoing birth cohort study with study participants still being under follow-up. All the study participants signed informed consent form; and the study protocol and its data and analyses are under the oversight of two IRBs: Johns Hopkins university and Boston Medical Center. Access to the BBC data (including the raw mtDNA sequencing data, the phenotype data used, and the data on mtDNA generated in this study) can be obtained from the corresponding author, Dr. Xiaobin Wang (email: xwang82@jhu.edu), after the data access request and a study proposal related to mtDNA and neurodevelopmental disorders are reviewed and approved by the two IRBs. A download link and access for the BBC data will be provided shortly after approval.

Field-specific reporting

Please select the one below that is the best fit for your research. If you are not sure, read the appropriate sections before making your selection.

☒ Life sciences ☐ Behavioural & social sciences ☐ Ecological, evolutionary & environmental sciences

For a reference copy of the document with all sections, see nature.com/documents/nr-reporting-summary-flat.pdf

Life sciences study design

All studies must disclose on these points even when the disclosure is negative.

Sample size	No statistical methods were used to compute sample size in the SSC. Data from all available families in the SSC with both parents, probands and siblings were used. Sample size in the BBC was determined based on our previous study in the SSC (Wang et al. PLoS Genetics, 2016) and would guarantee 80% power to identify an increase in mtDNA heteroplasmies (one-sided alpha=0.05) at an OR (odds ratio) =1.8 in cases.
Data exclusions	We excluded samples as well as related samples from the same family or mother-child pair using pre-established criteria in the SSC and BBC. Poor quality samples as determined by de novo mutation counts in mtDNA, average read depth in mtDNA and nuclear DNA, and mtDNA haplogroup analyses were excluded. Moreover, families who requested to withdraw from the SSC were excluded. Mother-child pairs in the BBC with incomplete data on sex or disease phenotypes were excluded from analyses as well. The number of samples excluded is described in detail in the manuscript.
Replication	102 pairs of technical replicates in the BBC were measured. The true positive rate was 100% (5,003/5,003) for detecting homoplasmies and was 98.6% (70/71) for detecting heteroplasmies (correlation in variant allele fraction [VAF] of the heteroplasmies: r>0.99 and ρ=0.96). The heteroplasmy that was not replicated to have VAF>0.2% was excluded from analysis.
Randomization	This is an observational study. No randomization was used.
Blinding	In the SSC, analyses were performed using predefined family and subject identifiers of the SSC containing information on sample relatedness and group allocation. In the BBC, all samples were given a pseudo-identifier unique to the current study and investigators were blinded to phenotypic information during sequencing experiments, sequencing data processing and quality filtering.

Reporting for specific materials, systems and methods

We require information from authors about some types of materials, experimental systems and methods used in many studies. Here, indicate whether each material, system or method listed is relevant to your study. If you are not sure if a list item applies to your research, read the appropriate section before selecting a response.

Materials & experimental systems

n/a	Involved in the study
☒	☐ Antibodies
☒	☐ Eukaryotic cell lines
☒	☐ Palaeontology and archaeology
☒	☐ Animals and other organisms
☐	☒ Human research participants
☐	☒ Clinical data
☒	☐ Dual use research of concern

Methods

n/a	Involved in the study
☒	☐ ChIP-seq
☒	☐ Flow cytometry
☒	☐ MRI-based neuroimaging

Human research participants

Policy information about studies involving human research participants

Population characteristics

The Simons Foundation Autism Research Initiative (SFARI) recruited families with unaffected parents and one or more children affected with autism for the SSC. The median age of children with autism in the SSC was 9.0 y (interquartile range [IQR]: 6.7-11.8 y) and 13% were female. The median age of nonautistic siblings in the SSC was 9.4 y (IQR=6.7-12.8 y) and 53% were female. For the BBC, it is a predominantly US, urban, low-income, mother-child dyads with ~60% Blacks and ~25% Hispanics, residing in Boston, MA. The median length of postnatal follow-up of children in the current study was 9.9 y (IQR=6.9-12.9 y) and 45% were female.

Recruitment

Families in the SSC were recruited by self-enrollment or physician referral. Mother-infant pairs in the BBC were enrolled shortly after delivery at the Boston Medical Center by trained research staff after informed consent since 1998 and were followed prospectively up to age 21 years. In both the SSC and BBC, all families included in the current study were "simplex'" families where only one child was affected with autism. Our observation may not apply to families with multiple children affected with autism.

Ethics oversight

The genome sequencing study of the SSC was initially approved by the institutional review board (IRB) at the New York Genome Center (Biomedical Research Alliance of New York [BRANY] IRB File # 17-08-26-385). Our research using only pre-existing de-identified data in the SSC was exempted from IRB review (Protocol ID# 1703007002) for human subject research by the IRB office at Cornell University prior to the current study. For the BBC, it has received initial and annual continuation approval from the Institutional Review Board of the Boston Medical Center (IRB Number: H-23237) and Johns Hopkins Bloomberg School of Public Health (IRB Number: 3966/CR513).

Note that full information on the approval of the study protocol must also be provided in the manuscript.

Clinical data

Policy information about clinical studies

All manuscripts should comply with the ICMJE guidelines for publication of clinical research and a completed CONSORT checklist must be included with all submissions.

Clinical trial registration

Although the BBC is an observational study, we did register at clinicaltrials.gov.

Study protocol

Registration #: NCT03228875
Website: https://clinicaltrials.gov/ct2/show/NCT032288

Data collection

Mother-infant pairs were enrolled shortly after delivery and followed prospectively up to age 21 years.

Outcomes

For the BBC, the primary outcomes were physician diagnosed ASD, ADHD, and other developmental disabilities as documented in the EMR.
